# [^18^F]FDG-PET/CT to prevent futile surgery in indeterminate thyroid nodules: a blinded, randomised controlled multicentre trial

**DOI:** 10.1007/s00259-021-05627-2

**Published:** 2022-01-04

**Authors:** Elizabeth J. de Koster, Lioe-Fee de Geus-Oei, Adrienne H. Brouwers, Eveline W. C. M. van Dam, Lioe-Ting Dijkhorst-Oei, Adriana C. H. van Engen-van Grunsven, Wilbert B. van den Hout, Tamira K. Klooker, Romana T. Netea-Maier, Marieke Snel, Wim J. G. Oyen, Dennis Vriens, for the EfFECTS trial study group

**Affiliations:** 1 Department of Radiology and Nuclear Medicine, Radboud University Medical Centre, Nijmegen, the Netherlands; 2 Department of Radiology, Section of Nuclear Medicine, Leiden University Medical Center, Leiden, the Netherlands; 3 Biomedical Photonic Imaging Group, University of Twente, Enschede, the Netherlands; 4 Department of Nuclear Medicine and Molecular Imaging Groningen, University of Groningen, University Medical Centre Groningen, Groningen, the Netherlands; 5 Department of Internal Medicine, Division of Endocrinology, Amsterdam University Medical Centers, Location VU Medical Center, Amsterdam, the Netherlands; 6 Department of Internal Medicine, Meander Medical Centre, Amersfoort, the Netherlands; 7 Department of Pathology, Radboud University Medical Centre, Nijmegen, the Netherlands; 8 Department of Medical Decision Making, Leiden University Medical Center, Leiden, the Netherlands; 9 Department of Endocrinology and Metabolism, Amsterdam Gastroenterology Endocrinology and Metabolism, Amsterdam University Medical Centers, Location Academic Medical Center, Amsterdam, the Netherlands; 10 Department of Internal Medicine, Flevo Hospital, Almere, the Netherlands; 11 Department of Internal Medicine, Division of Endocrinology, Radboud University Medical Centre, Nijmegen, the Netherlands; 12 Department of Medicine, Division of Endocrinology, Leiden University Medical Center, Leiden, the Netherlands; 13 Department of Radiology and Nuclear Medicine, Rijnstate Hospital, Arnhem, the Netherlands; 14 Department of Biomedical Sciences and Humanitas Clinical and Research Centre, Department of Nuclear Medicine, Humanitas University, Milan, Italy

**Keywords:** [^18^F]FDG-PET/CT, Indeterminate, Thyroid nodule, Thyroid carcinoma, Thyroid cytology, Thyroid surgery

## Abstract

**Purpose:**

To assess the impact of an [^18^F]FDG-PET/CT-driven diagnostic workup to rule out malignancy, avoid futile diagnostic surgeries, and improve patient outcomes in thyroid nodules with indeterminate cytology.

**Methods:**

In this double-blinded, randomised controlled multicentre trial, 132 adult euthyroid patients with scheduled diagnostic surgery for a Bethesda III or IV thyroid nodule underwent [^18^F]FDG-PET/CT and were randomised to an [^18^F]FDG-PET/CT-driven or diagnostic surgery group. In the [^18^F]FDG-PET/CT-driven group, management was based on the [^18^F]FDG-PET/CT result: when the index nodule was visually [^18^F]FDG-positive, diagnostic surgery was advised; when [^18^F]FDG-negative, active surveillance was recommended. The nodule was presumed benign when it remained unchanged on ultrasound surveillance. In the diagnostic surgery group, all patients were advised to proceed to the scheduled surgery, according to current guidelines. The primary outcome was the fraction of unbeneficial patient management in one year, i.e., diagnostic surgery for benign nodules and active surveillance for malignant/borderline nodules. Intention-to-treat analysis was performed. Subgroup analyses were performed for non-Hürthle cell and Hürthle cell nodules.

**Results:**

Patient management was unbeneficial in 42% (38/91 [95% confidence interval [CI], 32–53%]) of patients in the [^18^F]FDG-PET/CT-driven group, as compared to 83% (34/41 [95% CI, 68–93%]) in the diagnostic surgery group (*p* < 0.001). [^18^F]FDG-PET/CT-driven management avoided 40% (25/63 [95% CI, 28–53%]) diagnostic surgeries for benign nodules: 48% (23/48 [95% CI, 33–63%]) in non-Hürthle cell and 13% (2/15 [95% CI, 2–40%]) in Hürthle cell nodules (*p* = 0.02). No malignant or borderline tumours were observed in patients under surveillance. Sensitivity, specificity, negative and positive predictive value, and benign call rate (95% CI) of [^18^F]FDG-PET/CT were 94.1% (80.3–99.3%), 39.8% (30.0–50.2%), 95.1% (83.5–99.4%), 35.2% (25.4–45.9%), and 31.1% (23.3–39.7%), respectively.

**Conclusion:**

An [^18^F]FDG-PET/CT-driven diagnostic workup of indeterminate thyroid nodules leads to practice changing management, accurately and oncologically safely reducing futile surgeries by 40%. For optimal therapeutic yield, application should be limited to non-Hürthle cell nodules.

**Trial registration number:**

This trial is registered with ClinicalTrials.gov: NCT02208544 (5 August 2014), https://clinicaltrials.gov/ct2/show/NCT02208544.

**Supplementary Information:**

The online version contains supplementary material available at 10.1007/s00259-021-05627-2.

## Introduction

Thyroid nodules are common, but seldom harbour malignancy [1, 2]. Ultrasonography and fine needle aspiration cytology (FNAC) adequately differentiate benign from malignant thyroid nodules in approximately 70% of patients, but diagnostic dilemmas remain for nodules with indeterminate cytology, including atypia of undetermined significance or follicular lesion of undetermined significance (Bethesda III, AUS/FLUS) and (suspicious for a) follicular neoplasm (Bethesda IV, FN/SFN) or Hürthle cell neoplasm (Bethesda IV, HCN/SHCN) [2, 3]. The follicular lesions of which this group largely consists require histopathological assessment of capsular and vascular invasion to obtain a conclusive benign or malignant diagnosis [3]. Current international guidelines recommend repeat FNAC in Bethesda III nodules and consideration of clinical and ultrasound characteristics and patient preference in both Bethesda III and IV nodules, before deciding to proceed with either active surveillance or diagnostic surgery [3, 4]. When diagnostic surgery is performed, a mere one in four indeterminate thyroid nodules harbours malignancy. Thus, the surgery is futile in approximately 75% of these patients, with associated morbidity, risk of surgical complications, higher health care costs, and possible negative influence on the patients’ health-related quality of life (HRQoL) [3, 5–7]. A more accurate preoperative differentiation is needed to avoid futile diagnostic surgeries for benign nodules.

Positron emission tomography/computed tomography (PET/CT) using 2-[^18^F]fluoro-2-deoxy-D-glucose ([^18^F]FDG) visualises metabolic activity in tissues. A meta-analysis of the earlier small, non-randomised studies demonstrated that [^18^F]FDG-PET/CT reliably ruled out malignancy with 95% sensitivity in indeterminate thyroid nodules, increasing to 100% for nodules above 15 mm in diameter [5]. Consequently, [^18^F]FDG-PET/CT-driven management may cost-effectively reduce the fraction of futile surgeries from ~ 75% to ~ 40%, with an expected reduction in direct healthcare costs while maintaining HRQoL [5, 7]. More recent studies reported sensitivities ranging from 71% to 100%, with most trials confirming the safety of [^18^F]FDG-PET/CT-driven management [8, 9]. International guidelines acknowledged the potential but stopped short of recommending the routine use of [^18^F]FDG-PET/CT for indeterminate thyroid nodules, as randomised controlled trials underpinning the impact of [^18^F]FDG-PET/CT on improved patient outcomes are lacking [4].

Here, we report the first randomised controlled trial evaluating the implementation of [^18^F]FDG-PET/CT as a rule-out test in the diagnostic workup of indeterminate thyroid nodules. The primary objective was to accurately reduce unbeneficial patient management, i.e., avoid diagnostic surgery for benign nodules and avoid surveillance for malignant and borderline nodules requiring surgical resection. Secondary objectives were to determine the impact of [^18^F]FDG-PET/CT-driven management on the surgical complication rate, HRQoL, societal costs, and consequences of incidental PET/CT findings and to assess the implementability of [^18^F]FDG-PET/CT.

## Material and methods

### Study design and participants

The *Efficacy of [*^*18*^*F]FDG-PET in Evaluation of Cytological indeterminate Thyroid nodules prior to Surgery* (*EfFECTS) trial* was a blinded, randomised controlled multicentre trial performed in all eight academic and seven large community hospitals in the Netherlands (Supplementary Data p3). At all study sites, local investigators and physicians were highly experienced in the multidisciplinary diagnosis and treatment of thyroid nodules and thyroid carcinoma and worked in accordance with national and international guidelines [4, 10]. Adult euthyroid patients in whom diagnostic surgery was scheduled for an indeterminate thyroid nodule, defined as Bethesda III (confirmed on two subsequent FNAC procedures) or Bethesda IV cytology, were eligible for study participation [3]. Bethesda III or IV diagnosis was established by blinded central review by two dedicated thyroid pathologists (AE and BK). Prior to inclusion in the trial, clinical and ultrasound characteristics of the index nodule were considered in a multidisciplinary setting by the local physicians to establish the indication for diagnostic surgery, in accordance with current guidelines [4]. Patients were excluded from study participation if they had contraindications for [^18^F]FDG-PET/CT or a higher a priori risk of thyroid malignancy based on their presentation or history (i.e., unexplained stridor, vocal cord paralysis or radiation exposure to the thyroid), if they already underwent any non-routine preoperative diagnostic stratification (i.e., [^18^F]FDG-PET/CT or molecular diagnostics) or were unable to undergo randomisation (e.g., patient preference for surgery) [11]. Full eligibility criteria are listed in the study protocol (Supplementary Data). Written informed consent was obtained from all participants prior to any study activity. The study protocol was approved by the Medical Research Ethics Committee on Research Involving Human Subjects region Arnhem-Nijmegen, Nijmegen, the Netherlands. The trial was overseen by a trial steering committee and an independent study safety committee. The funder of the study had no role in its design, data collection and analysis, or writing of this report.

### Randomisation

Patients were individually randomly assigned to the [^18^F]FDG-PET/CT-driven group or diagnostic surgery group in a 2:1 ratio. To pursue an even distribution of risk factors for differentiated thyroid carcinoma across both arms, stratification was applied by patient sex, age (dichotomised at 45 years), ultrasonographic thyroid nodule size (0–10, 11–20, 21–40, or > 40 mm), Bethesda classification (III or IV), and study site. Randomisation was performed in the trial management system, Castor Electronic Data Capture (Castor EDC, Amsterdam, the Netherlands), which uses a validated variable block randomisation model.

### Procedures

All patients underwent an [^18^F]FDG-PET/CT of the neck, acquired by 20 PET/CT scanners at 12 EARL-accredited study sites (Supplementary Table 1) using a standard acquisition and reconstruction protocol in accordance with European Association of Nuclear Medicine (EANM) guidelines [11, 12]. In summary, patients fasted for at least 6 h prior to the injection of the radiopharmaceutical (activity adjusted for patient body weight, time per bed position, and PET/CT scanner sensitivity). Approximately 60 min (range 55–70 min) after intravenous [^18^F]FDG administration, patients were scanned from the external acoustic meatus to the aortic arch in a supine position, with at least 2 min per bed position. A low-dose non-contrast–enhanced CT scan was performed. EARL-reconstructed, pseudonymised scans were stored in a central, password-protected online database within the National Biomedical Imaging Archive environment (NBIA, National Cancer Institute, Bethesda, MD, USA). Next, scans were centrally assessed by two independent, experienced nuclear medicine physicians (LG and DV). Any focal [^18^F]FDG-uptake within the thyroid that was visually higher than the background [^18^F]FDG-uptake of the surrounding normal thyroid tissue and that corresponded to the index thyroid nodule in size and location was considered positive. To support the visual assessment, [^18^F]FDG-uptake was quantified using maximum and peak (ø1-cm sphere) standardised uptake values (SUV_max_, SUV_peak_), using body weight for normalization. In case of a discordant assessment, a third reviewer (WO) was consulted for a consensus meeting. All image analyses were performed using OsiriX Lite DICOM-viewer (Pixmeo SARL, Bernex, Switzerland).

One project team member (EK) combined patient allocation, and the [^18^F]FDG-PET/CT result to a preformulated treatment advice. A written report containing only this advice was presented to the patient’s local physician; the patient’s allocation and [^18^F]FDG-PET/CT result remained concealed. If present, incidental [^18^F]FDG-PET/CT-findings outside the index nodule and with potential diagnostic and/or therapeutic consequences were also reported in an appendix to the report, to be evaluated by the local physician in the context of the patients’ medical history. In the [^18^F]FDG-PET/CT-driven group, the treatment advice was based on the [^18^F]FDG-PET/CT results. When the index nodule was [^18^F]FDG-positive, patients were advised to proceed to the scheduled diagnostic surgery. When the nodule was [^18^F]FDG-negative, patients were advised to refrain from surgery and undergo active surveillance of the nodule, which was defined as at least one follow-up ultrasound exam of the neck and outpatient clinic visit after one year. Any additional follow-up visits during study participation were permitted at the discretion of the local physician. The nodule was presumed benign when it remained unchanged in size and appearance on the one-year ultrasound. In case of significant growth (> 50% volume change or > 20% increase in at least two dimensions, excluding cystic components) or changed ultrasound appearance including newly observed suspicious characteristics, further evaluation by repeat FNAC was recommended. Suspicious ultrasound characteristics were defined as a marked hypoechoic solid nodule, irregular shape (i.e., taller-than-wide), irregular margins (i.e., lobulated, infiltrative), and/or presence of microcalcifications.

In the diagnostic surgery group, the treatment advice for all patients was to proceed to the scheduled diagnostic surgery, in accordance with the current international guidelines [4, 10]. In both study groups, the patient and his/her physician were free to deviate from the study treatment advice at any time.

All postoperative patient management was based on the local histopathological diagnosis and current international guidelines [4]. After completion of all study procedures and data collection, all histopathology was centrally reviewed by a dedicated thyroid pathologist (AE). In case of a discordant review, a second central pathologist was consulted for a consensus meeting. Incidentally detected (micro)carcinomas located outside the index nodule were not considered for the main outcome measures.

HRQoL and societal costs were assessed during one year, calculated from the date of the [^18^F]FDG-PET/CT scan. Patients were asked to complete the EuroQol 5-dimension 5-level questionnaire (EQ-5D-5L), the iMTA Medical Consumption Questionnaire (iMCQ), and the iMTA Productivity Costs Questionnaire (iPCQ) at 0 (baseline), 3, 6, and 12 months (Supplementary Data p9) [13–16]. Societal costs (in €) included all direct medical costs for thyroid-related and other healthcare consumption, patient costs (i.e., informal care, travel expenses), and productivity losses. Volumes of healthcare consumption were extracted from individual patient files and the iMCQ. Costs were valued using reference prices and the 2019 reimbursement rates of the Dutch System of Diagnosis-Treatment Combinations, where appropriate and available (Supplementary Data p10). The estimated cost of one partial-body [^18^F]FDG-PET/CT scan was €754 [17, 18]. All [^18^F]FDG-PET/CT-related costs, including the costs of the scan itself and any additional healthcare consumption for incidental [^18^F]FDG-PET/CT findings, were only taken into account for the [^18^F]FDG-PET/CT-driven group.

### Blinding

Patients, all local study site personnel, and all pathologists were blinded to [^18^F]FDG-PET/CT results and allocation. Central pathologists were additionally blinded to the local cyto- and histopathological diagnoses. Central nuclear medicine physicians were blinded to allocation and all clinicopathological data except for the ultrasonographic size and location of the index nodule. Other study investigators assessing outcomes were blinded to allocation. Patients allocated to the [^18^F]FDG-PET/CT-driven group with an [^18^F]FDG-negative nodule could inevitably deduce their allocation and [^18^F]FDG-PET/CT result from the surveillance advice.

### Outcomes

The primary outcome was the fraction of patient management that was considered unbeneficial one year after the [^18^F]FDG-PET/CT scan. Unbeneficial management was defined as futile diagnostic surgery for histopathologically benign nodules (including hyperplastic nodules, follicular adenoma, and Hürthle cell adenoma) and/or unjustified surveillance for histopathological malignant or borderline tumours. According to novel insights arising during the trial, nodules diagnosed as noninvasive follicular thyroid neoplasm with papillary-like nuclear features (NIFTP) or follicular tumour of uncertain malignant potential (FT-UMP) are considered benign yet potentially premalignant (i.e., borderline) lesions, for which surgery is considered justified [19, 20]. Following broad acceptance of these insights, we added a refinement concerning these borderline tumours to the study protocol during the trial. As histopathology was not reviewed until trial completion, this modification did not in any way influence trial execution and primary endpoints. The results for the primary outcome for a strictly benign-malignant differentiation are reported in the Supplementary Data (p16).

Secondary outcomes of the trial included the differences in surgical complication rates, one-year HRQoL expressed in quality-adjusted life years (QALYs), and societal costs (in €) between both strategies. The diagnostic accuracy of [^18^F]FDG-PET/CT (whole-group analysis) was estimated: [^18^F]FDG-positive or [^18^F]FDG-negative nodules confirmed as malignant or borderline tumours on histopathology were considered true-positive or false-negative, respectively; [^18^F]FDG-positive or [^18^F]FDG-negative nodules confirmed as benign on histopathology and those that remained unchanged on the one-year ultrasound were considered false-positive or true-negative, respectively. Finally, the number of incidental [^18^F]FDG-PET/CT findings with diagnostic and/or therapeutic consequences in the scanned area (descriptive whole-group analysis), implementability of [^18^F]FDG-PET/CT (i.e., diagnostic confidence) defined as the fraction of patients not reassured by a negative [^18^F]FDG-PET/CT result, and survival were assessed. Per protocol, the follow-up for all endpoints was set at one year after [^18^F]FDG-PET/CT. Whenever available and relevant, data beyond one year of follow-up (censored 1 October 2021) are presented.

### Statistical analysis

The trial was designed to have 80% power to detect a reduction in unbeneficial management from ~ 75% to ~ 40% at a significance level of 0.05. At least 90 evaluable patients with nodules > 10 mm were required (2:1 allocation). After correction for 82.7% expected nodule size > 10 mm and 15% estimated data-attrition, the sample size was set at 132 patients [5, 7].

After half of the anticipated patients of the diagnostic surgery group were recruited, the study safety committee conducted a predetermined interim analysis and reported no objections to safe continuation of the trial.

The applied descriptive statistics were mean ± standard deviation or median and interquartile range for continuous variables and absolute numbers and relative frequencies (%) for categorical variables. Intention-to-treat analysis was performed. Categorical primary and secondary outcomes were compared between allocated groups using Pearson’s chi-squared or Fisher’s exact tests, where appropriate. We adjusted for the stratifying variables using binary logistic regression; the corrected *p* values are reported together with an adjusted odds ratio and their 95% confidence intervals (CI) [21].

Sensitivity, specificity, and negative and positive predictive value (NPV, PPV) were calculated using the traditional formulas. 95% CIs were calculated using the β-distribution (Clopper-Pearson interval). For EQ-5D-5L, iMCQ, and iPCQ questionnaires, we used multiple imputation to account for possible selectively missing values. To estimate HRQoL, we calculated Dutch utility scores from the EQ-5D-5L and estimated the mean one-year QALYs as the area under the utility curves (Supplementary Data p8). One-year societal costs were estimated as the mean sum of [volume x costs] for all components. QALYs and costs are presented as mean and 95% CI and compared using independent samples T-tests with unequal variances. In the analysis of QALYs and costs, we adjusted for the stratifying variables and additionally adjusted for possible influences of the unevenly distributed malignancy/borderline rate (see Results, and Supplementary Tables 4 and 5), using a generalized linear model (GLM). The *local* benign/malignant histopathological diagnosis was included in the GLM as a covariate, as this diagnosis determined the patients’ postoperative course of treatment and thus contributed to the costs and perceived HRQoL. The adjusted means, *p* values, and mean differences are presented.

Two prespecified subgroup analyses for the primary outcome were performed: one for nodules > 10 mm (ultrasonographic largest diameter) and one for Hürthle cell nodules (defined as Bethesda IV HCN/SHCN cytology) and non-Hürthle cell nodules (defined as Bethesda III AUS/FLUS and Bethesda IV FN/SFN cytology).

Data collection was performed using Castor EDC (Castor EDC, Amsterdam, the Netherlands). Statistical analysis was performed using SPSS Statistics version 26 (IBM Corp, Armonk, NY, USA). This trial is registered with ClinicalTrials.gov NCT02208544 (5 August 2014).

## Results

**Fig. 1:**
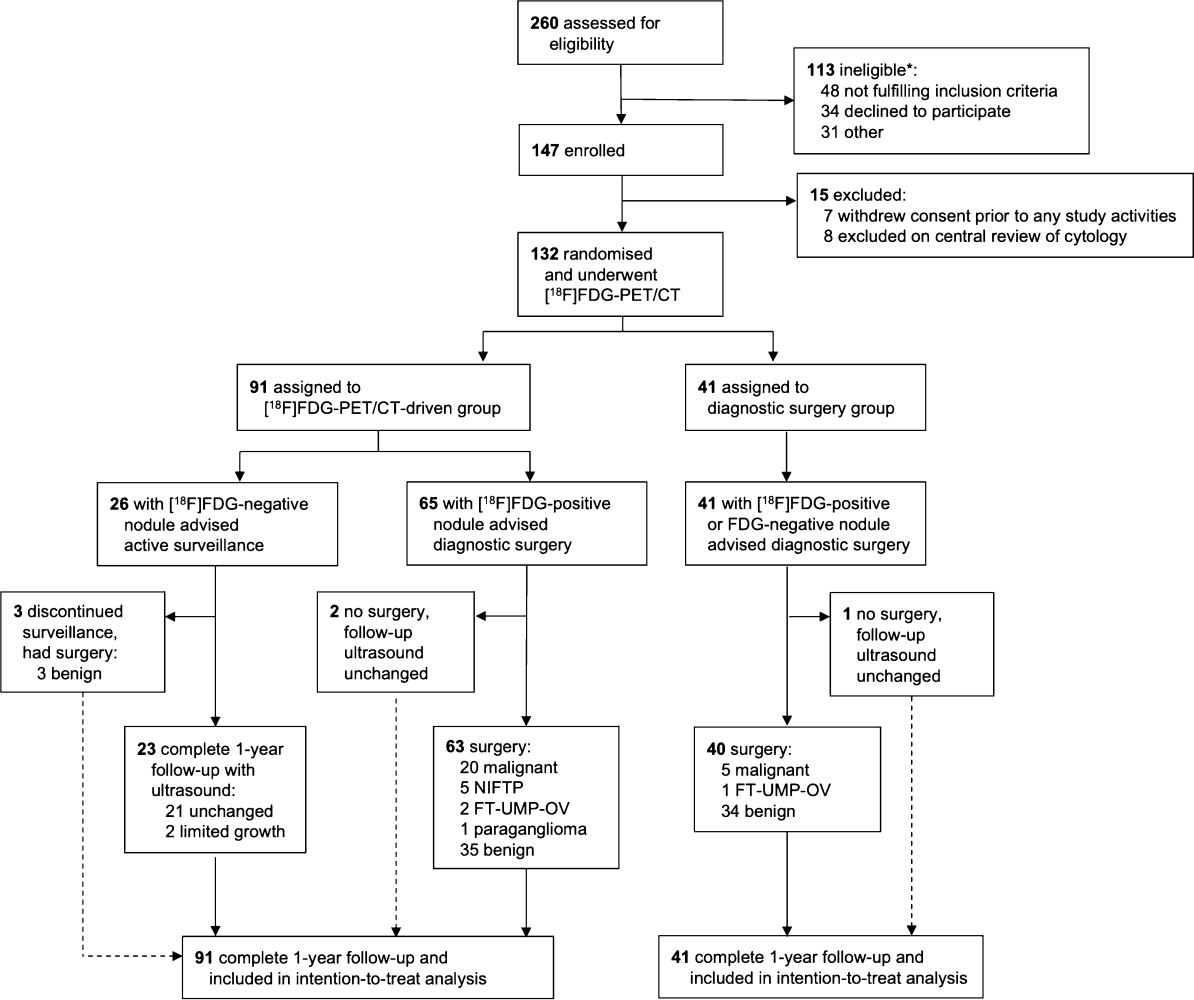
Trial profile. The dashed line indicates the patients who deviated from the treatment advise per protocol. NIFTP, non-invasive follicular thyroid neoplasm with papillary-like nuclear features. FT-UMP-OV, follicular tumour of uncertain malignant potential, Hürthle cell type. *: a specification of reasons for ineligibility is provided in Supplementary Table 2

After screening 260 patients for eligibility, we finally enrolled 132 patients with a cytologically indeterminate thyroid nodule and scheduled diagnostic thyroid surgery between 1 July 2015 and 16 October 2018 (Fig. 1). Their mean age was 54.5 ± 13.6 years; 107 (81.1%) patients were female. A total of 91 (69%) patients were randomly allocated to the [^18^F]FDG-PET/CT-driven group and 41 (31%) to the diagnostic surgery group. Baseline characteristics, including stratifying variables and PET/CT parameters, were balanced across both allocation groups, except for two patient-reported complaints upon the first presentation: subjectively increased size of a known thyroid nodule (*p* = 0.01) and dysphagia (*p* = 0.02) (Table 1). Suspicious ultrasound characteristics were present at baseline in 40% (36/91) patients in the [^18^F]FDG-PET/CT-driven group and 46% (19/41) patients in the diagnostic surgery group (*p* = 0.47).

**Table 1:** Baseline characteristics of the study population and [^18^F]FDG-PET/CT parameters

		[^18^F]FDG-PET/CT-driven group	diagnostic surgery group
	n	***n*** = 91	***n*** = 41
Female^a^	132	73 (80%)	34 (83%)
Mean age in years ± SD^a^	132	54.3 ± 14.6	54.5 ± 11.6
**Complaints of thyroid nodule**			
Painless swelling in the neck	132	55 (60%)	27 (66%)
Incidental finding on imaging	132	24 (26%)	6 (15%)
Growth of known nodule	132	3 (3%)	7 (17%)
Hoarseness	132	3 (3%)	5 (12%)
Dyspnoea or pressure on trachea	132	11 (12%)	8 (20%)
Dysphagia	132	11 (12%)	12 (29%)
Fatigue	132	2 (2%)	1 (2%)
Fear of malignancy	132	2 (2%)	1 (2%)
Cosmetic complaints	132	2 (2%)	0 (0%)
No complaints	132	11 (12%)	4 (10%)
**Physical examination**			
Palpable thyroid nodule	127	72 (81%)	32 (84%)
**Thyroid function**	**125**		
Median TSH, mU/L (IQR)^b^	125	1.65 (1.20–2.35)	1.54 (0.94–2.40)
Median fT4, pmol/L (IQR)^c^	94	15.0 (13.3–16.7)	14.3 (13.0–15.7)
**Ultrasound characteristics**			
Solitary nodule	132	64 (70%)	29 (71%)
Multinodular disease	132	27 (30%)	12 (29%)
Median size, mm (IQR)^a^	132	36 (23–45)	31 (22–39)
Suspicious characteristics^d^	132	36 (40%)	19 (46%)
Solid hypoechoic nodule		23 (25%)	15 (37%)
Taller-than-wide shape		0 (0%)	1 (2%)
Irregular margins		7 (8%)	2 (5%)
Microcalcifications		12 (13%)	4 (10%)
**Cytology**^a^	**132**		
Bethesda III	60	40 (44%)	20 (49%)
Bethesda IV	72	51 (56%)	21 (51%)
FN/SFN	41	28 (31%)	13 (32%)
HCN/SHCN	31	23 (25%)	8 (20%)
**[**^**18**^**F]FDG-PET/CT**			
[^18^F]FDG-positive	132	65 (71%)	26 (63%)
Median SUV_max_ of the nodule, g/cm^3^ (IQR)	132	4.0 (2.8–10.6)	3.7 (2.3–8.2)
Median SUV_peak_ of the nodule, g/cm^3^ (IQR)	132	3.4 (2.3–8.4)	2.9 (1.9–5.9)
Median SUV_max_ of thyroid background, g/cm^3^ (IQR)	132	1.9 (1.7–2.5)	2.0 (1.7–2.5)
Median SUV_max_ ratio (IQR)	132	2.3 (1.3–6.2)	1.8 (1.1–3.9)
Median SUV_peak_ ratio (IQR)	132	1.9 (1.1–4.8)	1.4 (0.9–2.7)

FN/SFN, (suspicious for a) follicular neoplasm. fT4, free thyroxine. HCN/SHCN, (suspicious for a) Hürthle cell neoplasm. IQR, interquartile range. SD, standard deviation. SUV_max_, maximum standardised uptake value. SUV_max_ ratio, ratio between SUV_max_ of the index nodule and background SUV_max_ of the surrounding normal thyroid tissue. SUV_peak_, peak (ø1-cm sphere) standardised uptake value. SUV_peak_ ratio, ratio between SUV_peak_ of the index nodule and background SUV_max_ of the surrounding normal thyroid tissue. TSH, thyroid-stimulating hormone

^a^Included as stratifying variable in the stratified randomisation

^b^The reference range for TSH is 0.4–4.0 mU/L

^c^The reference range for fT4 is approximately 10–25 pmol/L (sex and age dependent)

^d^Suspicious ultrasound characteristics were defined as presence of at least one of the following characteristics: marked hypoechogenicity (in a solid nodule), irregular shape (i.e., taller-than-wide), irregular margins, and/or presence of microcalcifications

[^18^F]FDG-PET/CT results showed a visually [^18^F]FDG-negative index nodule in 41 of 132 (31%) patients: 26 of 91 (29%) in the [^18^F]FDG-PET/CT-driven group and 15 of 41 (37%) in the diagnostic surgery group (*p* = 0.36). All 26 patients with an [^18^F]FDG-negative index nodule in the [^18^F]FDG-PET/CT-driven group were advised active surveillance. After one year, 23 had not undergone surgery. On the one-year ultrasound, 21 of 23 nodules (91%) were unchanged in size and appearance; they were considered benign. Two of 23 (9%) nodules had increased by 28–37% in largest diameter on the one-year ultrasound. To date, after a median follow-up of 29 months (IQR 24–45) until their latest ultrasound exam, 20 of 23 (87%) nodules have remained unchanged. Three patients, including the two with an apparently growing nodule on the one-year ultrasound, experienced local discomfort attributed to local compression of the nodule and underwent diagnostic surgery outside the study follow-up (20, 35, and 41 months after [^18^F]FDG-PET/CT, respectively). Histopathology was benign, showing two follicular adenomas and one hyperplastic nodule.

All 41 patients in the diagnostic surgery group and the 65 patients with an [^18^F]FDG-positive index nodule in the [^18^F]FDG-PET/CT-driven group were advised to proceed to the scheduled diagnostic surgery. One patient in the diagnostic surgery group and two patients in the [^18^F]FDG-PET/CT-driven group, all with [^18^F]FDG-positive nodules, waived surgery. To date, after 28–42 months of follow-up and repeated ultrasound exams, none of these nodules have changed; they are considered benign (false-positive).

In total, 106 of 132 (80.3%) patients underwent diagnostic surgery during study follow-up (Supplementary Table 4). The central review of the histopathology was discordant with the local diagnosis in six cases (6%) (Supplementary Table 5). A total of 34 (26%) nodules had a histopathological diagnosis that justified surgery, including 25 malignancies, five NIFTP, three FT-UMP, and one paraganglioma. A total of 72 (55%) nodules had benign histopathology. In addition to the 26 nodules that were presumed benign during active surveillance, in total, 98 of 132 (74.2%) nodules were considered benign: 63 of 91 (69%) in the [^18^F]FDG-PET/CT-driven group and 35 of 41 (85%) in the diagnostic surgery group. Despite successful stratified randomisation, the rate of malignant/borderline nodules appeared higher in the [^18^F]FDG-PET/CT-driven group (28/91, 31%) than in the diagnostic surgery group (6/41, 15%). After adjusting for the stratifying variables, the difference was not statistically significant (*p* = 0.08). All patients completed all study-related procedures and one year of follow-up. There were no adverse events.

### Primary outcomes

After one year of follow-up, patient management had been unbeneficial in 38 of 91 (42% [95% CI, 32–53%]) patients in the [^18^F]FDG-PET/CT-driven group, compared to 34 of 41 (83% [95% CI, 68–93%]) patients in the diagnostic surgery group (*p* < 0.001, OR 0.1 [95% CI, 0.1–0.4]). These were all futile diagnostic surgeries for histopathologically benign nodules. There was no unjustified surveillance: no malignancies or borderline tumours were observed in patients under surveillance. [^18^F]FDG-PET/CT-driven management avoided surgery for 25 of 63 (40% [95% CI, 28–53%]) benign nodules. In comparison, only one of 35 (3% [95% CI, 0–15%]) patients in the diagnostic surgery group did not undergo the recommended surgery and was considered benign on ultrasound follow-up (*p* = 0.002, OR 26.9 [95% CI, 3.3–219.0]) (Table 2).

**Table 2:** Therapeutic yield after one year of follow-up

	[^18^F]FDG-PET/CT-driven group ***n*** = 91		diagnostic surgery group ***n*** = 41				
	*n*	% (95% CI)	*n*	% (95% CI)	*p*^a^	Adjusted *p*^b^	Adjusted OR (95% CI)^b^
**Beneficial management**	53 / 91	58% (47-68%)	7 / 41	17% (7-32%)	* <* *0.001*	* < 0.001*	*6.9 (2.7–17.7)*
Surgery for malignant/borderline nodule	28 / 91	31% (22-41%)	6 / 41	15% (6-29%)	*0.05*	0.08	2.5 (0.9–7.0)
Surveillance for benign nodule	25 / 91	27% (19-38%)	1 / 41	2% (0-13%)	*0.001*	*0.007*	*17.3 (2.2–135.2)*
**Unbeneficial management**	38 / 91	42% (32-53%)	34 / 41	83% (68-93%)	* < 0.001*	* < 0.001*	*0.1 (0.1–0.4)*
Surgery for benign nodule	38 / 91	42% (32-53%)	34 / 41	83% (68-93%)	* < 0.001*	* < 0.001*	*0.1 (0.1–0.4)*
Surveillance for malignant/borderline nodule	0 / 91	0% (0-4%)	0 / 41	0% (0-9%)	n.a.	n.a.	n.a.
**Avoided surgery in benign nodules**	25 / 63	40% (28-53%)	1 / 35	3% (0-15%)	*0.001*	*0.002*	*26.9 (3.3–219.0)*

CI, confidence interval. n.a., not applicable. OR, odds ratio

^a^Pearson’s chi-squared test

^b^Binary logistic regression to adjust for stratifying variables

### Secondary outcomes

Sensitivity, specificity, NPV, PPV, and benign call rate of [^18^F]FDG-PET/CT were 94.1% (95% CI, 80.3–99.3%), 39.8% (95% CI, 30.0–50.2%), 95.1% (95% CI, 83.5–99.4%), 35.2% (95% CI, 25.4–45.9%), and 31.1% (95% CI, 23.3–39.7%), respectively (Table 3). Two of 132 (1.5%) [^18^F]FDG-PET/CT scans were false-negative (both in the diagnostic surgery group). In both cases, the corresponding index nodules had caused extensive debate during the blinded interpretation of the histopathology (i.e., benign or malignant diagnosis). One was a 15 mm, RAS-mutated, non-invasive neoplasm with uncommon spindle cell metaplasia, which was ultimately classified as a papillary thyroid carcinoma (PTC, TNM pT1b). The other was a 32 mm, predominantly cystic, non-invasive neoplasm with a solid component of 8 mm. It was only considered malignant (follicular variant of PTC, TNM pT2) after detection of an ETV6-NTRK3 fusion during the central review of the histopathology (details provided in the Supplementary Data p18).

**Table 3:** Diagnostic accuracy parameters, including results for non-Hürthle and Hürthle cell subgroups^a^

	*n*	TP	FP	TN	FN	Sensitivity		Specificity		NPV		PPV		Benign call rate	
							(95% CI)		(95% CI)		(95% CI)		(95% CI)		(95% CI)
**All**	132	32	59	39	2	94.1%	(80.3–99.3%)	39.8%	(30.0–50.2%)	95.1%	(83.5–99.4%)	35.2%	(25.4–45.9%)	31.1%	(23.3–39.7%)
**Non-Hürthle cell nodules**															
AUS/FLUS + FN/SFN	101	23	38	38	2	92.0%	(74.0–99.0%)	50.0%	(38.3–61.7%)	95.0%	(83.1–99.4%)	37.7%	(25.6–51.0%)	39.6%	(30.0–49.8%)
AUS/FLUS	60	9	25	25	1	90.0%	(55.5–99.7%)	50.0%	(35.5–64.5%)	96.2%	(80.4–99.9%)	26.5%	(12.9–44.4%)	43.3%	(30.6–56.8%)
FN/SFN	41	14	13	13	1	93.3%	(68.1–99.8%)	50.0%	(29.9–70.1%)	92.9%	(66.1–99.8%)	51.9%	(31.9–71.3%)	34.1%	(20.1–50.6%)
**Hürthle cell nodules**															
HCN/SHCN	31	9	21	1	0	100%	(66.4–100%)	4.5%	(0.1–22.8%)	100%	(2.5–100%)	30.0%	(14.7–49.4%)	3.2%	(0.1–16.7%)

AUS/FLUS, atypia of undetermined significance or follicular lesion of undetermined significance. CI, confidence interval. FN, false-negative. FN/SFN, (suspicious for a) follicular neoplasm. FP, false-positive. HCN/SHCN, (suspicious for a) Hürthle cell neoplasm. PPV, positive predictive value. NPV, negative predictive value. TN, true-negative. TP, true-positive

^a^Whole-group analysis was performed to estimate diagnostic accuracy parameters

No difference in the surgical complication rate was observed between both groups. Still, following the reduction in diagnostic surgeries, the rate of new levothyroxine suppletion-dependent hypothyroidism due to partial thyroidectomy procedures was only 6% (5/86) in the [^18^F]FDG-PET/CT-driven group as compared to 17% (7/41) in the diagnostic surgery group (*p* = 0.07, OR 0.3 [95% CI, 0.1–1.1]). Other surgical complications infrequently occurred (Table 4).

**Table 4:** Secondary outcomes

	[^18^F]FDG-PET/CT-driven group (***n*** = 91)	Diagnostic surgery group (***n*** = 41)	*p*	Adjusted *p*	Adjusted OR (95% CI)
**Surgical complications following diagnostic surgery**	13 (14%)	10 (24%)^a^	0.16^e^	0.17^g^	0.5 (0.2–1.3)^g^
In benign nodules (n = 98)	9 (14%)	9 (26%)	0.16^e^	0.23^g^	0.5 (0.2–1.5)^g^
In malignant and borderline nodules (n = 34)	4 (14%)	1 (17%)	1^f^	0.75^g^	1.6 (0.1–30.7)^g^
Type of complication					
Wound infection	1 (1%)	1 (2%)	0.53^f^	0.62^g^	0.5 (0.0–8.4)^g^
Hematoma with re-exploration surgery	1 (1%)	1 (2%)^a^	0.53^f^	0.53^g^	0.4 (0.0–7.8)^g^
Seroma	1 (1%)	1 (2%)	0.53^f^	0.63^g^	0.5 (0.0–8.7)^g^
Recurrent nerve paralysis	2 (2%)	0 (0%)	1^f^	1^g^	2.4E+7 (0-∞)^g^
Hypothyroidism following partial thyroidectomy^b^	5 (6%)^c^	7 (17%)^a^	0.06^f^	0.07^g^	0.3 (0.1–1.1)^g^
Hypoparathyroidism, transient	3 (3%)^d^	1 (2%)	1^f^	0.85^g^	1.2 (0.1–13.2)^g^
**Incidental findings on [**^**18**^**F]FDG-PET/CT**					
With diagnostic or therapeutic consequence	12 (13%)	10 (24%)	0.11^e^	0.09^g^	0.4 (0.2–1.1)^g^
[^18^F]FDG-positive thyroid incidentaloma	10 (11%)	9 (22%)	0.10^e^	0.10^g^	0.4 (0.2–1.2)^g^
Benign	7 (8%)	7 (17%)	0.32^e^	n.s.	
Malignant	2 (2%)	0 (0%)			
No definite diagnosis	1 (1%)	2 (5%)			
**Survival after one year**	91 (100%)	41 (100%)	n.a.	n.a.	n.a.

CI, confidence interval. n.a., not applicable. n.s., not specified. OR, odds ratio

^a^Two complications (hematoma and hypothyroidism) occurred in one patient

^b^Hypothyroidism due to partial thyroidectomy included patients who had new levothyroxine-dependent hypothyroidism following a partial thyroidectomy procedure (i.e., hemithyroidectomy and/or isthmus resection)

^c^Initial total thyroidectomies (*n* = 5) are excluded from the denominator

^d^Transient hypoparathyroidism only occurred following initial total thyroidectomy

^e^Pearson’s chi-squared test

^f^Fisher’s exact test

^g^Binary logistic regression to adjust for stratifying variables

EQ-5D-5L questionnaires were completed by 69 of 91 (76%) patients in the [^18^F]FDG-PET/CT-driven and 29 of 41 (71%) patients in the diagnostic surgery group (*p* = 0.54). Perceived HRQoL during the first year after the [^18^F]FDG-PET/CT scan was similar in both groups. Adjusted for the stratifying variables and malignancy/borderline rate, a mean of 0.793 (95% CI, 0.753–0.833) QALYs was estimated in the [^18^F]FDG-PET/CT-driven group, as compared to 0.725 (0.651–0.799) QALYs in the diagnostic surgery group (*p* = 0.11). The adjusted mean societal costs during the first year were significantly lower in the [^18^F]FDG-PET/CT-driven group than the diagnostic surgery group: €14,800 (95% CI, + €12,600– + €17,000) as compared to €21,700 (+ €16,800– + €26,600) per patient, respectively, with an adjusted mean difference of − €6,900 (-€12,100– − €1,600, *p* = 0.01) (Table 5 and Supplementary Data p8-10).

**Table 5:** Secondary outcomes: HRQoL and societal costs

	[^18^F]FDG-PET/CT-driven group (***n*** = 91)	Diagnostic surgery group (***n*** = 41)	*p*	**Mean difference (95% CI)**
**HRQoL**				
Mean one-year QALYs from EQ-5D-5L (95% CI)	0.792 (0.749–0.836)	0.727 (0.663–0.791)	0.13^a^	0.065 (− 0.018– + 0.159)^a^
* Adjusted* mean one-year QALYs from EQ-5D-5L (95% CI)	0.793 (0.753–0.833)	0.725 (0.651–0.799)	0.11^b^	0.068 (− 0.015– + 0.151)^b^
**Societal costs**				
Mean one-year societal costs (95% CI)	€15,500 (+ €12,600– + €18,500)	€20,100 (+ €15,500– + €24,700)	0.13^a^	− €4,600 (− €10,500– + €1,300)^a^
* Adjusted* mean one-year societal costs (95% CI)	€14,800 (+ €12,600– + €17,000)	€21,700 (+ €16,800– + €26,600)	*0.01*^*b*^	− €6,900 (− €12,100– − €1,600)^b^

CI, confidence interval. HRQoL, health-related quality of life. QALYs, quality-adjusted life years

^a^Independent samples t-test with unequal variances

^b^Generalized linear model, adjusted analysis for stratifying variables and malignancy/borderline rate based on the local histopathological diagnosis

Incidental [^18^F]FDG-PET/CT findings with diagnostic or therapeutic consequences were reported for 22 of 132 (17%) [^18^F]FDG-PET/CT scans (Table 4, Supplementary Data p26-27). These included 21 [^18^F]FDG-positive thyroid incidentalomas in 19 (14%) patients, for which 13 additional FNAC procedures were performed. Eleven of 21 (52%) incidentalomas were surgically resected. Two ipsilateral incidentalomas were malignant. In four patients, their initially scheduled hemithyroidectomy was extended to a total thyroidectomy to include a contralateral incidentaloma; all were histopathologically benign: one follicular adenoma and three hyperplastic nodules. These total thyroidectomy procedures in 4 of 132 (3%) of patients are considered overtreatment due to the [^18^F]FDG-PET/CT.

Diagnostic confidence in [^18^F]FDG-PET/CT was high: only one of six patients who underwent surgery (three during and three after study follow-up) despite advised surveillance (Fig. 1) was not fully reassured by the negative [^18^F]FDG-PET/CT result. The main reason for surgery in all six patients, however, was not the fear or suspicion of cancer but increasing compressive symptoms causing discomfort. Noncompliance to the surveillance advice did not change the one-year therapeutic yield in the [^18^F]FDG-PET/CT-driven group, as patient crossover between surgical and non-surgical management occurred in both directions (Fig. 1). Based on theoretical full compliance to the given treatment advice, a maximum 41% reduction in futile diagnostic surgeries for benign nodules (i.e., 26 [^18^F]FDG-negative nodules of 63 benign nodules) was estimated following full implementation of [^18^F]FDG-PET/CT (*p* = 0.86).

Subgroup analysis of nodules > 10 mm (128/132 patients, excluding two 10 mm nodules from each group) demonstrated similar therapeutic yield and diagnostic accuracy as compared to the main results (Supplementary Data p12).

Subgroup analysis of the 101 non-Hürthle cell nodules (60 AUS/FLUS and 41 FN/SFN) and 31 Hürthle cell (HCN/SHCN) nodules was performed. The malignant/borderline rate was 17% (10/60) in Bethesda III as compared to 33% (24/72) in Bethesda IV nodules (*p* = 0.03), of which 37% (15/41) in FN/SFN and 29% (9/31) in HCN/SHCN nodules (*p* = 0.50).

In non-Hürthle cell nodules, the fractions of unbeneficial management and prevented surgeries for benign nodules after one year were 37% (95% CI, 25–49%) and 48% (95% CI, 33–63%) in the [^18^F]FDG-PET/CT-driven group, as compared to 85% (95% CI, 68–95%) (*p* < 0.001) and 0% (95% CI, 0–18%) (*p* < 0.001) in the diagnostic surgery group (Table 6). Sensitivity, specificity, NPV, PPV, and benign call rate in non-Hürthle cell nodules were 92.0% (95% CI, 74.0–99.0%), 50.0% (95% CI, 38.3–61.7%), 95.0% (95% CI, 83.1–99.4%), 37.7% (95% CI, 25.6–51.0%), and 39.6% (95% CI, 30.0–49.8%), respectively (Table 3). Therapeutic yield and diagnostic accuracy were similar in AUS/FLUS and FN/SFN nodules. In Hürthle cell nodules, [^18^F]FDG-PET/CT showed a benign call rate of only 3.2% (1/31). Consequently, [^18^F]FDG-PET/CT-driven management was not contributory to improve the diagnostic workup: the fractions of unbeneficial management and prevented surgeries for benign Hürthle cell nodules were low and similar in both study groups (*p* = 1) and included one patient in each group who declined the advised surgery (Table 6). [^18^F]FDG-PET/CT-driven management avoided significantly more futile surgeries in non-Hürthle cell nodules (48% [95% CI, 33–63%]) than in Hürthle cell nodules (13% [95% CI, 2–40%]) (*p* = 0.02).

**Table 6:** Subgroup analysis: therapeutic yield after one year of follow-up in AUS/FLUS, FN/SFN, and HCN/SHCN nodules

	[^18^F]FDG-PET/CT-driven group		Diagnostic surgery group		
	n	% (95% CI)	n	% (95% CI)	*p*
**Non-Hürthle cell nodules, AUS/FLUS + FN/SFN (n = 101)**	**n = 68**		**n = 33**		
** Beneficial management**	43/68	63% (51–75%)	5 / 33	15% (5–32%)	* < 0.001*^*a*^
Surgery for malignant/borderline nodule	20/68	29% (19–42%)	5 / 33	15% (5–32%)	0.12^a^
Surveillance for benign nodule	23/68	34% (23–46%)	0 / 33	0% (0–11%)	< *0.001*^*a*^
** Unbeneficial management**	25/68	37% (25–49%)	28 / 33	85% (68–95%)	* < 0.001*^*a*^
Surgery for benign nodule	25/68	37% (25–49%)	28 / 33	85% (68–95%)	< *0.001*^*a*^
Surveillance for malignant/borderline nodule	0/68	0% (0–5%)	0 / 33	0% (0–11%)	n.a.
** Avoided surgery in benign nodules**	23/48	48% (33–63%)	0 / 28	0% (0–12%)	* < 0.001*^*a*^
**AUS/FLUS (n = 60)**	**n = 40**		**n = 20**		
** Beneficial management**	24/40	60% (43–75%)	1 / 20	5% (0–25%)	* < 0.001*^*a*^
Surgery for malignant/borderline nodule	9/40	23% (11–38%)	1 / 20	5% (0–25%)	0.14^b^
Surveillance for benign nodule	15/40	38% (23–54%)	0 / 20	0% (0–17%)	*0.002*^*a*^
** Unbeneficial management**	16/40	40% (25–57%)	19 / 20	95% (75–100%)	* < 0.001*^*a*^
Surgery for benign nodule	16/40	40% (25–57%)	19 / 20	95% (75–100%)	< *0.001*^*a*^
Surveillance for malignant/borderline nodule	0/40	0% (0–9%)	0 / 20	0% (0–17%)	n.a.
** Avoided surgery in benign nodules**	15/31	48% (30–67%)	0 / 19	0% (0–18%)	* < 0.001*^*a*^
**FN/SFN (n = 41)**	**n = 28**		**n = 13**		
** Beneficial management**	19/28	68% (48–84%)	4 / 13	31% (9–61%)	*0.03*^***a***^
Surgery for malignant/borderline nodule	11/28	39% (22–59%)	4 / 13	31% (9–61%)	0.73^b^
Surveillance for benign nodule	8/28	29% (13–49%)	0 / 13	0% (0–25%)	*0.04*^*b*^
** Unbeneficial management**	9/28	32% (16–52%)	9 / 13	69% (39–91%)	*0.03*^***a***^
Surgery for benign nodule	9/28	32% (16–52%)	9 / 13	69% (39–91%)	*0.03*^*a*^
Surveillance for malignant/borderline nodule	0/28	0% (0–12%)	0 / 13	0% (0–25%)	n.a.
** Avoided surgery in benign nodules**	8/17	47% (23–72%)	0 / 9	0% (0–34%)	*0.02*^***b***^
**Hürthle cell nodules, HCN/SHCN (n = 31)**	**n = 23**		**n = 8**		
** Beneficial management**	10/23	43% (23–66%)	2 / 8	25% (3–65%)	0.43^b^
Surgery for malignant/borderline nodule	8/23	35% (16–57%)	1 / 8	13% (0–53%)	0.38^b^
Surveillance for benign nodule	2/23	9% (1–28%)	1 / 8	13% (0–53%)	1^b^
** Unbeneficial management**	13/23	57% (34–77%)	6 / 8	75% (35–97%)	0.43^b^
Surgery for benign nodule	13/23	57% (34–77%)	6 / 8	75% (35–97%)	0.43^b^
Surveillance for malignant/borderline nodule	0/23	0% (0–15%)	0 / 8	0% (0–37%)	n.a.
** Avoided surgery in benign nodules**	2/15	13% (2–40%)	1 / 7	14% (0–58%)	1^b^

AUS/FLUS, atypia of undetermined significance or follicular lesion of undetermined significance FN/SFN, cytology (suspicious for a) follicular neoplasm. HCN/SHCN, (suspicious for a) Hürthle cell neoplasm n.a., not applicable

^a^Pearson’s chi-squared test

^b^Fisher’s exact test

## Discussion

The *EfFECTS trial* demonstrated that [^18^F]FDG-PET/CT-driven management resulted in 40% avoided futile surgeries for benign nodules after one year. The high 94.1% sensitivity of [^18^F]FDG-PET/CT ensures that omitting diagnostic surgery does not compromise oncological safety. Despite patient cross-over between surgical and non-surgical management strategies, these results are in line with our previous meta-analysis, in which we estimated that [^18^F]FDG-PET/CT-driven management could accomplish a reliable maximum 47% reduction in diagnostic surgeries for benign nodules [5, 7]. The secondary outcomes of the trial showed significantly lower one-year societal costs of [^18^F]FDG-PET/CT-driven management, amply compensating the additional costs of the [^18^F]FDG-PET/CT (€754) by other cost-savings. Combined with similar HRQoL in both groups, a high likelihood of cost-effectiveness of an [^18^F]FDG-PET/CT-driven diagnostic workup is suggested. Finally, a trend towards fewer cases of postoperative medication-dependent hypothyroidism after hemithyroidectomy was demonstrated.

The Hürthle cell nodules in our population were nearly all [^18^F]FDG-positive, irrespective of malignant or benign histopathology. Visual assessment of [^18^F]FDG-PET/CT did not contribute to any reduction of futile surgeries in this subgroup. To prevent the unbeneficial application of [^18^F]FDG-PET/CT and optimise its therapeutic yield, it should only be offered to patients with non-Hürthle cell AUS/FLUS or FN/SFN cytology. Nodules with Hürthle cell cytology should be excluded from visual analysis with [^18^F]FDG-PET/CT. Any benefits of quantitative [^18^F]FDG-PET/CT assessment methods, such as SUV-derived analysis, texture analysis, and radiomics, have shown potential in indeterminate thyroid nodules and [^18^F]FDG-positive thyroid incidentaloma, although the current evidence is limited and further studies are required [22–24]. Other diagnostics, such as molecular analysis for specific driver mutations, mitochondrial DNA mutations, and copy number variations, should be considered for Hürthle cell nodules [25–27].

The earlier [^18^F]FDG-PET/CT studies repeatedly demonstrated sensitivities up to 100%, while more recent studies did report some missed cancer diagnoses [5, 8, 9, 28]. A recent meta-analysis hypothesised that the progress from stand-alone PET to hybrid PET/CT techniques likely increased the false-negative rate because PET/CT provides a better anatomical correlation [29]. Although [^18^F]FDG uptake in ipsilateral multinodular disease could complicate exact anatomical correlation, we consider this a highly unlikely explanation. The improved spatial resolution and decreased detection limit (now ~ 10 mm diameter to reliably exclude [^18^F]FDG-uptake) of newer PET/CT scanners likely results in fewer false-negative as well as more false-positive readings. Rather than the impact of improved technology, between-study heterogeneity may result from varying thresholds for the definition of an [^18^F]FDG-negative nodule, in combination with global variations in case-mix, ranging from variable malignancy rates to differences in histopathological subtypes, genomic patterns, and altered protein expression levels related to the glycolysis pathway [25, 30].

Other diagnostics can be considered for indeterminate nodules [25]. Even though they were initially developed for pre-FNAC risk assessment of thyroid nodules, various ultrasound classification systems, such as the American Thyroid Association (ATA) and Thyroid Imaging Reporting and Data System (TIRADS) classifications, have increasingly demonstrated their added diagnostic value in nodules with indeterminate cytology [31]. TIRADS assessment may also improve the diagnostic accuracy of [^18^F]FDG-PET/CT [8, 9]. The current study focussed purely on [^18^F]FDG-PET/CT: patients were only included after their indication for diagnostic surgery was established based on cytology, clinical and ultrasound parameters (in accordance with international guidelines) to prevent undesirable interference of considerations regarding ultrasound characteristics when aiming to assess the impact of [^18^F]FDG-PET/CT-driven management, our primary objective. At the time when the current study was initiated in 2015, TIRADS was less established and it was only implemented very limitedly in the Netherlands. Its prospective assessment was not part of the study procedures. We considered it inappropriate to retrospectively reassess baseline stored ultrasound captures as ultrasound is a dynamic technique.

Molecular diagnostics are undeniably gaining traction in clinical practice and are increasingly applied in the preoperative workup of thyroid nodules. Besides aiding the differentiation between benign and malignant, these have an added advantage of risk stratification based on the type of genetic alteration found [32]. However, few tests meet the rule-out and/or rule-in requirements for a safe implementation of an ancillary test [4, 25]. [^18^F]FDG-PET/CT meets this rule-out criterium (i.e., false-negative rate lower than or equal to a benign (Bethesda II) cytological diagnosis), as do some commercial gene mutation classifiers with similar sensitivity. These panels appear to outperform [^18^F]FDG-PET/CT on specificity and benign call rate but have major downsides with regard to their limited global availability and very high costs per patient (a Medicare reimbursement rate of $3,600 = €3,109; €1 = $1.18 on 01–10-2021), in addition to practical challenges concerning the required quality, quantity, and storage of the cytological material [33, 34]. In a European setting, with relatively limited costs for surgery and hospital admission, cost-effectiveness of these commercial molecular tests seems unattainable [7]. Locally developed and less comprehensive European molecular panels are available, but their diagnostic accuracy appears too limited for routine application in daily practice [35–37]. Due to large global variations in local healthcare expenses, case-mix, and availability of techniques, cost-utility, and convenience of any diagnostic workup will greatly vary among different healthcare systems [7, 25, 38, 39]. Following previous model-based assumptions and the significant difference in costs that is demonstrated in the current study, life-long real-world cost-effectiveness of [^18^F]FDG-PET/CT is currently being modelled using the results of the *EfFECTS* trial [7].

Implementability of [^18^F]FDG-PET/CT was assessed in patients with an [^18^F]FDG-negative nodule in the [^18^F]FDG-PET/CT-driven group, who could deduce their allocation and [^18^F]FDG-PET/CT result from the surveillance advice. In spite of the suspense of participating in a clinical trial, the observed high therapy compliance reflects the patients’ and physicians’ diagnostic confidence and adoption of [^18^F]FDG-PET/CT as a trustworthy diagnostic tool. During study participation as well as afterwards, compressive symptoms were the principal reason for surgery in patients with a negative [^18^F]FDG-PET/CT result and surveillance advice. This demonstrated that shared decision-making remains crucial to select patients for [^18^F]FDG-PET/CT who would not prefer surgery for discomfort from compressive symptoms, fear of malignancy or other reasons, optimize the (long-term) therapeutic yield of [^18^F]FDG-PET/CT, and limit unbeneficial use of resources.

A potential limitation of our study is its per protocol one-year follow-up period for [^18^F]FDG-negative nodules. Concerns about missed cancer diagnoses were ameliorated by the extended median ultrasound follow-up of 29 months. Whether any very slow-growing malignant or borderline thyroid tumours in these nodules will lower diagnostic accuracy of [^18^F]FDG-PET/CT and limit the [^18^F]FDG-PET/CT-driven reduction in futile diagnostic surgeries remains to be established. Similarly, additional long-term false-negative results were recently reported for a well-known molecular classifier [40]. It seems clinically unlikely that a delayed diagnosis of any missed slow-growing malignancies or borderline tumours following [^18^F]FDG-PET/CT-driven management will alter the patients’ prognosis, as such tumours are likely indolent in nature. False-negative results in previous [^18^F]FDG-PET/CT studies in indeterminate nodules mostly concerned low-risk (T1) cancers [8, 9, 28]. In differentiated thyroid carcinoma, [^18^F]FDG-uptake is inversely related to prognosis, and [^18^F]FDG-negative carcinomas showed fewer aggressive characteristics on histopathology [41, 42]. In the current study, the two reported false-negative nodules were difficult to establish and only classified as malignant after extensive assessments including molecular analysis by multiple expert thyroid pathologists.

Limitations of the routine use of [^18^F]FDG-PET/CT include the limited worldwide availability of PET/CT scanners and adherence to standardised international scanning protocols, the use of low levels of ionizing radiation (~ 4 mSv), and the diagnostic and therapeutic consequences (including costs) of incidental findings. Our study showed that incidental findings caused overtreatment in 4 of 132 (3%) patients, even though treatment was compliant with the current guidelines (Supplementary Data p26). These individual cases underpin that careful exploration of further diagnostic options should be considered, especially when drastic management changes are the consequence.

In conclusion, this randomised controlled trial shows that an [^18^F]FDG-PET/CT-driven diagnostic workup of indeterminate thyroid nodules leads to practice changing patient management, accurately and oncologically safely ruling out malignancy, reducing futile surgeries by 40%, and saving approximately €6,900 per patient. Its use should be limited to nodules with non-Hürthle cell cytology only to further optimise its therapeutic yield to 48%, as [^18^F]FDG-PET/CT does not contribute to the management of patients with Hürthle cell nodules.

## Supplementary Information

Below is the link to the electronic supplementary material.

Supplementary file1 (PDF 12085 KB)

Supplementary file2 (PDF 2827 KB)

## Data Availability

The study protocol and datasets generated during and/or analysed during the current study are available from the corresponding author on reasonable request. Data requestors will need to sign a data access agreement and in keeping with patient consent for secondary use and obtain ethical approval for any new analyses.
