# Supplementary material for: [18F]FDG-PET/CT to prevent futile surgery in indeterminate thyroid nodules: a blinded, randomised controlled multicentre trial
Source: Eur J Nucl Med Mol Imaging. 2022 Jan 4;49(6):1970–84. doi: 10.1007/s00259-021-05627-2 (PMC9016050; doi:10.1007/s00259-021-05627-2)
Supplement: Supplementary file 1 — Supplementary file1 (PDF 12085 KB) [file 259_2021_5627_MOESM1_ESM.pdf]

Supplementary Data 1.

[^{18}F]FDG-PET/CT to prevent futile surgery in indeterminate thyroid nodules: a blinded, randomised controlled multicentre trial

Elizabeth J. de Koster, Lioe-Fee de Geus-Oei, Adrienne H. Brouwers, Eveline W.C.M. van Dam, Lioe-Ting Dijkhorst-Oei, Adriana C.H. van Engen-van Grunsven, Wilbert B. van den Hout, Tamira K. Klooker, Romana T. Netea-Maier, Marieke Snel, Wim J.G. Oyen, and Dennis Vriens,

for the *EfFECTS trial* study group.

Table of contents

EfFECTS trial study group **3.**

Trial steering committee 3.

Local principal investigators 3.

Study safety committee 4.

Supplementary Data **5.**

Supplementary Table 1: Included patients per study site and PET/CT scanners 5.

Supplementary Figure 1: Included patients per study site and allocated group 6.

Supplementary Table 2: Reasons for patient ineligibility for study participation 7.

HRQoL assessments and analysis 8.
Supplementary Table 3: HRQoL estimates from EQ-5D-5L and VAS 8.
Supplementary Figure 2: HRQoL estimates from EQ-5D-5L and VAS 9.

Cost assessment and analysis 10.

Supplementary Table 4: Types of diagnostic surgery and histopathological diagnoses 11.

Supplementary Table 5: Discordant histopathology diagnoses 11.

Subgroup analysis for nodules >10 mm 12.
Supplementary Table 6: FDG-PET/CT parameters 12.
Supplementary Table 7: Therapeutic yield after one year of follow-up in nodules 12.
Supplementary Table 8: Diagnostic accuracy parameters for nodules >10 mm 13.
Supplementary Table 9: Secondary outcomes for nodules >10 mm 13.
Supplementary Table 10: Secondary outcomes for nodules >10 mm: HRQoL and societal costs 14.
Supplementary Table 11: Therapeutic yield after one year of follow-up in AUS/FLUS, FN/SFN and HCN/SHCN nodules 15.
Subgroup analysis: Differentiation between strictly benign and malignant nodules 16.
Supplementary Table 12: Benign and malignant diagnosis per group 16.
Supplementary Table 13: Therapeutic yield after one year of follow-up 16.
Supplementary Table 14: Therapeutic yield after one year of follow-up in AUS/FLUS, FN/SFN and HCN/SHCN 17.

Nodules with false-negative [^{18}F]FDG-PET/CT 18.

Incidental [^{18}F]FDG-PET/CT findings 26.

Supplementary Table 15: Incidental [^{18}F]FDG-PET/CT findings 27.

References of the appendix 28.

EfFECTS trial study group

Trail steering committee

- Prof. dr. L.F. de Geus-Oei, MD PhD (**project leader**), *Leiden University Medical Center, Department of Radiology, Section of Nuclear Medicine, Leiden, the Netherlands; Radboud University Medical Centre, Department of Radiology and Nuclear Medicine, Nijmegen, the Netherlands*
- Prof. dr. W.J.G. Oyen, MD PhD (**principal investigator**), *Radboud University Medical Centre, Department of Radiology and Nuclear Medicine, Nijmegen, the Netherlands; Rijnstate Hospital, Department of Radiology and Nuclear Medicine, Arnhem, the Netherlands; Department of Biomedical Sciences and Humanitas Clinical and Research Centre, Department of Nuclear Medicine, Humanitas University, Milan, Italy*
- Dr. D. Vriens, MD PhD (**principal investigator**), *Leiden University Medical Center, Department of Radiology, Section of Nuclear Medicine, Leiden, the Netherland.*
- E.J. de Koster, MD (**junior investigator**), *Radboud University Medical Centre, Department of Radiology and Nuclear Medicine, Nijmegen, the Netherlands*

Local principal investigators

Radboud university medical centre, Nijmegen, the Netherlands
- Dr. A.C.H. van Engen-van Grunsven, MD PhD, *Department of Pathology*
- E.J. de Koster, MD, *Department of Radiology and Nuclear Medicine*
- Dr. B. Küsters, MD PhD, *Department of Pathology*
- Prof. dr. R.T. Netea-Maier, MD PhD, *Department of Internal Medicine, Division of Endocrinology*
- Prof. dr. J.W.A. Smit, MD PhD, *Department of Internal Medicine, Division of Endocrinology*
- Prof. dr. J.H.W. de Wilt, MD PhD, *Department of Surgical Oncology*

Amsterdam University Medical Centers, Amsterdam, the Netherlands
Location Academic Medical Center
- Prof. dr. J. Booij, MD PhD, *Department of Radiology and Nuclear Medicine*
- Prof. dr. E. Fliers, MD PhD, *Department of Endocrinology and Metabolism*
- Dr. T.K. Klooker, MD PhD, *Department of Endocrinology and Metabolism*

Location VU University Medical Center
- Dr. E.W.C.M. van Dam, MD PhD, *Department of Internal Medicine, Division of Endocrinology*
- Dr. K.M.A. Dreijerink, MD PhD, *Department of Internal Medicine, Division of Endocrinology*
- Dr. P.G.H.M. Raijmakers, MD PhD, *Department of Radiology and Nuclear Medicine*

Erasmus University Medical Centre, Rotterdam, the Netherlands
- Dr. B.L.R. Kam, MD PhD, *Department of Nuclear Medicine*
- Prof. dr. R.P. Peeters, MD PhD, *Department of Internal Medicine*
- Prof. dr. J. Verzijlbergen, MD PhD, *Department of Nuclear Medicine*

Haga Hospital, The Hague, the Netherlands
- Dr. M.O. van Aken, MD PhD, *Department of Internal Medicine*

Isala Hospital, Zwolle, the Netherlands
- Prof. dr. P.L. Jager, MD PhD, *Department of Nuclear Medicine*
- Dr. G.S. Mijnhout, MD PhD, *Department of Internal Medicine*

Leiden University Medical Center, Leiden, the Netherlands
- Prof. dr. L.F. de Geus-Oei, MD PhD, *Department of Radiology, Section of Nuclear Medicine*
- Dr. W.B. van den Hout, PhD, *Department of Biomedical Data Sciences-Medical Decision Making*
- Prof. dr. A.M. Pereira Arias, MD PhD, *Department of Internal Medicine, Division of Endocrinology*
- Prof. dr. J. Morreau, MD PhD, *Department of Pathology*
- Dr. M. Snel, MD PhD, *Department of Internal Medicine, Division of Endocrinology*
- Dr. D. Vriens, MD PhD, *Department of Radiology, Section of Nuclear Medicine*

Meander Medical Centre, Amersfoort, the Netherlands
- Dr. L.T. Dijkhorst-Oei, MD PhD, *Department of Internal Medicine*
- Dr. J.M.H. de Klerk, MD PhD, *Department of Nuclear Medicine*

Maastricht University Medical Centre, Maastricht, the Netherlands
- Dr. B. Havekes, MD PhD, *Department of Internal Medicine, Division of Endocrinology*
- Dr. D.C. Mitea, MD PhD, *Department of Radiology and Nuclear Medicine*
- Dr. S. Vöö, MD PhD, *Department of Radiology and Nuclear Medicine*

OLVG Hospital, Amsterdam, the Netherlands
- Dr. C.B. Brouwer, MD PhD, *Department of Internal Medicine*
- Dr. P.S. van Dam, MD PhD, *Department of Internal Medicine*
- Dr. F. Sivro, MD PhD, *Department of Nuclear Medicine*

Reinier de Graaf Hospital, Delft, the Netherlands
- Dr. E.T. te Beek, MD PhD, *Department of Nuclear Medicine*
- Dr. M.C.W. Jebbink, MD PhD, *Department of Internal Medicine*

Rijnstate Hospital, Arnhem, the Netherlands
- Dr. G.S. Bleumink, MD PhD, *Department of Internal Medicine*
- Prof. dr. W.J.G. Oyen, MD PhD, Department of Radiology and Nuclear Medicine
- Dr. V.J.R. Schelfhout, MD PhD, Department of Radiology and Nuclear Medicine

St. Antonius Hospital, Nieuwegein, the Netherlands
- Dr. R.G.M. Keijsers, MD PhD, *Department of Nuclear Medicine*
- Dr. I.M.M.J. Wakelkamp, MD PhD, *Department of Internal Medicine*

University Medical Centre Groningen, Groningen, the Netherlands
- Dr. A.H. Brouwers, MD PhD, *Department of Nuclear Medicine and Molecular Imaging*
- Prof. dr. T.P. Links, MD PhD, *Division of Endocrinology, Department of Internal Medicine*

University Medical Centre Utrecht, Utrecht, the Netherlands
- Dr. B. de Keizer, MD PhD, *Department of Radiology and Nuclear Medicine*
- Dr. R. van Leeuwaarde, MD PhD, *Department of Endocrine Oncology*

Study safety committee

- Dr. J.J. Bonenkamp, MD PhD, *Department of Surgical Oncology, Radboud University Medical Centre, Nijmegen, The Netherlands.*
- Dr. A.R.T. Donders, PhD, *Department for Health Evidence, Radboud University Medical Centre, Nijmegen, the Netherlands*
- Prof. dr. J.J. Fütterer, Phd, *Department of Radiology and Nuclear Medicine, Radboud University Medical Centre, Nijmegen, The Netherlands.*

Supplementary Table 1: Included patients per study site and PET/CT scanners

Study site	Number of included patients	PET/CT scanners
Radboud university medical centre, Nijmegen, the Netherlands	13	Siemens SOMATOM Definition AS<br>Siemens Biograph mCT 40<br>Philips Gemini TF 64
Amsterdam University Medical Centre, Amsterdam, the Netherlands<br>Location AMC	11	Philips Gemini TF 16<br>Philips Gemini GXL<br>Siemens Biograph mCT 128
Amsterdam University Medical Centre, Amsterdam, the Netherlands<br>Location VUMC	23	Philips Ingenuity TF<br>Philips Gemini TF 64
Erasmus University Medical Centre, Rotterdam, the Netherlands	7	Siemens Biograph mCT 40
Haga Hospital, The Hague, the Netherlands	4	Patients were scanned at Leiden University Medical Center
Isala Hospital, Zwolle, the Netherlands	5	Philips Ingenuity TF
Leiden University Medical Center, Leiden, the Netherlands	19	Philips Gemini TF 64<br>Siemens Biograph Horizon<br>Philips Vereos
Meander Medical Centre, Amersfoort, the Netherlands	18	Siemens Biograph mCT 40
Maastricht University Medical Centre, Maastricht, the Netherlands	2	Philips Gemini TF 64
OLVG Hospital, Amsterdam, the Netherlands	6	Patients were scanned at Amsterdam University Medical Center, location AMC
Reinier de Graaf Hospital, Delft, the Netherlands	0	Not applicable.
Rijnstate Hospital, Arnhem, the Netherlands	2	Philips Gemini TF 64
St. Antonius Hospital, Nieuwegein, the Netherlands	3	Philips Gemini TF 64
University Medical Centre Groningen, Groningen, the Netherlands	14	Siemens Biograph mCT 40<br>Siemens Biograph mCT 64
University Medical Centre Utrecht, Utrecht, the Netherlands	5	Siemens Biograph mCT 40
**TOTAL**	**132**	

*: Philips Medical Systems, Philips Healthcare, Best, the Netherlands
**: Siemens Healthineers, Erlangen, Germany

Supplemental Figure 1: Included patients per study site and allocated group

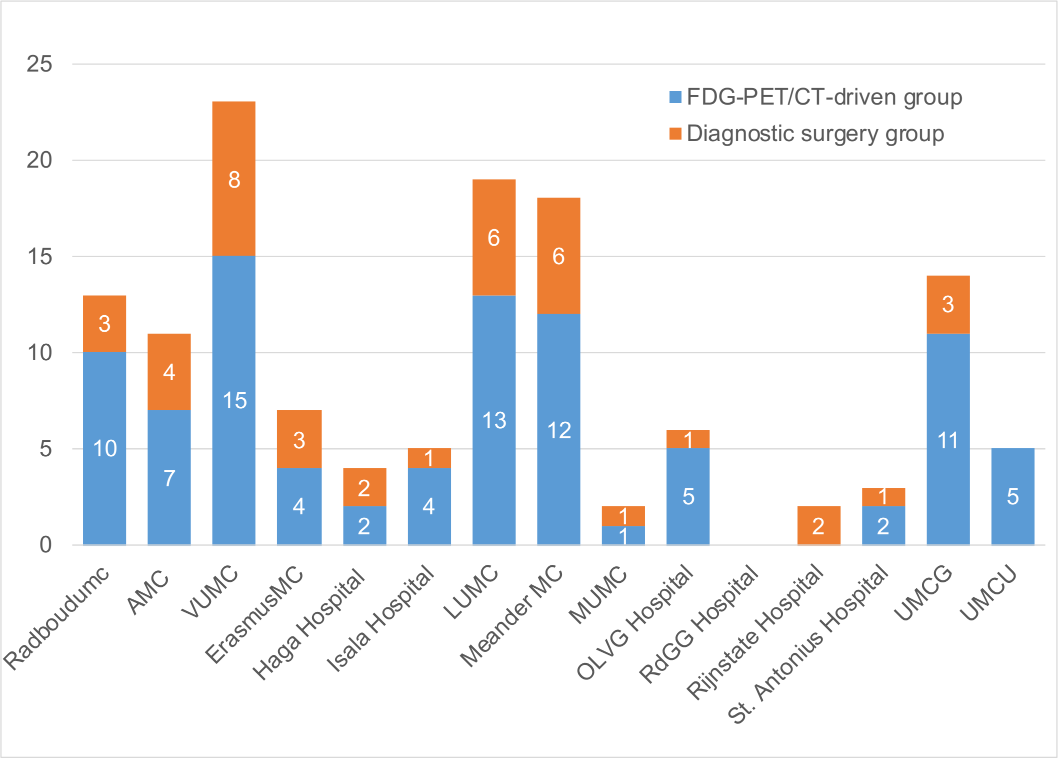

Supplementary Table 2: Reasons for patient ineligibility for study participation.

**Patients not fulfilling inclusion criteria**	**48**
Diagnostic surgery not scheduled/recommended yet*	13
Repeat FNAC Bethesda II	5
Repeat FNAC Bethesda VI	1
Unknown follow-up, no study inclusion	7
Patient preferred diagnostic surgery to study participation	11
Patient preferred surveillance to study participation	1
First presentation with suspicious cervical lymphadenopathy	3
Initial diagnosis of the thyroid nodule as an [^{18}F]FDG-positive thyroid incidentaloma on [^{18}F]FDG-PET/CT	1
Patient underwent any non-routine diagnostic test (e.g., mutation analysis, [^{18}F]FDG-PET/CT outside study)	5
Language barrier	3
Patient under 18 years old	1
Comorbidities / medical history	9
Pregnant	1
**Patient did not want to participate in (all aspects of) the study**	**34**
**Reason unknown to researchers, not reported by local physician**	**31**
**TOTAL ineligible patients**	**113**

*: In most of these cases, diagnostic surgery was not scheduled/recommended yet because patients only had one Bethesda III result. According to current guidelines, repeat FNAC is recommended before considering diagnostic surgery.

HRQoL assessments and analysis

Health-related quality of life (HRQoL) was assessed during one year, counted from the date of the [^{18}F]FDG-PET/CT scan. To estimate the HRQoL, patients were asked to complete the EuroQol 5-dimension 5-level questionnaire (EQ-5D-5L) at 0 (baseline), 3, 6, and 12 months, counted from the date of the [^{18}F]FDG-PET/CT scan [1]. Patients were given the option to complete either web-based questionnaires with email invitations, or paper questionnaires sent to their home address with stamped return envelopes included.

From filled questionnaires, we calculated utility scores using the EQ-5D-5L domain scores and the appropriate Dutch tariff [2]. Visual analogues scale (VAS) scores were transformed to utilities using the formula Utility = $1 - (1 - (VAS/100))^{1.61}$ [3].
We used multiple imputation to account for possible selectively missing values, using age (calculated as age at baseline), sex, allocation, EQ-5D-5L utility scores and time-dependent variables for thyroid surgery and benign or malignant local histopathological diagnosis as predictor variables.
Quality adjusted life years (QALYs) for the first year were estimated as the area under the utility curves [4]. Differences between randomisation groups were statistically analysed using independent samples t-tests with unequal variances. A generalized linear model (GLM) was used to adjust for the stratifying factors and malignancy rate; the adjusted p-value, corrected mean difference and 95% confidence interval are presented. As the postoperative treatment of the individual patients was based on the *local* histopathological diagnosis and potentially influences the perceived HRQoL, this diagnosis was included in the GLM.

EQ-5D-5L questionnaires were fully completed at all four measurements by 69 of 91 (76%) patients in the [^{18}F]FDG-PET/CT-driven and 29 of 41 (71%) patients in the diagnostic surgery group (p=0.54).
According to the EQ-5D-5L domain scores, the valuation of quality of life was similar in the [^{18}F]FDG-PET/CT-driven and diagnostic surgery groups at all four measurements (Supplementary Table 3).
QALYs estimated from both the EQ-5D-5L and the VAS were similar in the [^{18}F]FDG-PET/CT-driven and diagnostic surgery groups. Other results are presented in the main paper.

Supplementary Table 3: HRQoL estimates from EQ-5D-5L and VAS, crude data

	**[^{18}F]FDG-PET/CT driven group** (n=91)	**diagnostic surgery group** (n=41)	difference	p value
**EQ-5D-5L utilities:**				
Baseline	0.8522	0.7910	-0.0612	0.14[a]
3 months	0.8316	0.7621	-0.0695	0.11[a]
6 months	0.7489	0.6744	-0.0745	0.23[a]
12 months	0.7876	0.7385	-0.0491	0.33[a]
**QALYs**	**0.7922**	**0.7269**	**-0.0653**	**0.13[a]**
**Utilities from VAS:**				
Baseline	0.8771	0.8862	0.0091	0.80[a]
3 months	0.8823	0.8822	-0.0001	1[a]
6 months	0.8995	0.8368	-0.0627	0.04[a]
12 months	0.9044	0.8509	-0.0535	0.06[a]
**QALYs from VAS**	**0.8936**	**0.8579**	**-0.0357**	**0.08[a]**

QALYs=quality-adjusted life years. VAS=visual analogue scale.
[a]: independent samples t-test.

Supplementary Figure 2: HRQoL estimates from EQ-5D-5L

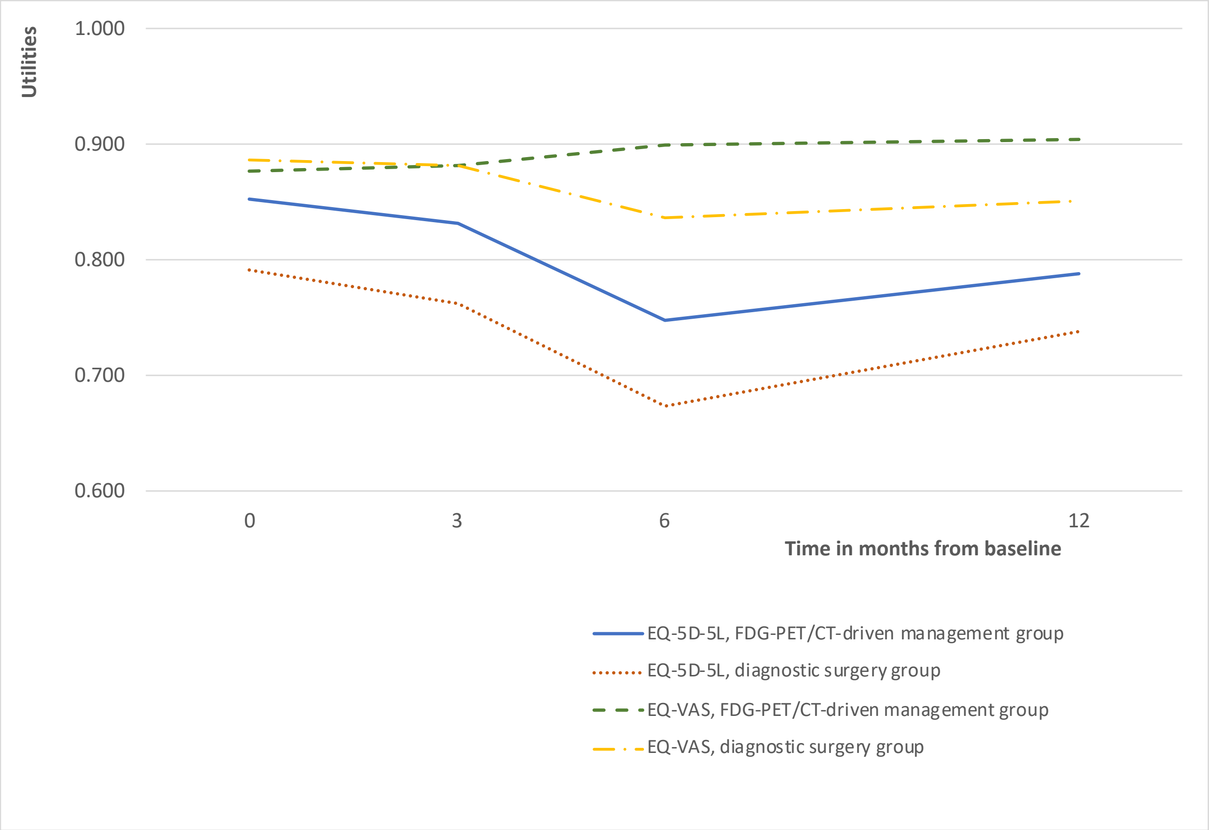

VAS=visual analogue scale.

Cost assessment and analysis

Societal costs (in €) were assessed during one year, calculated from the date of the [18F]FDG-PET/CT scan. Societal costs included all direct medical costs for thyroid-related and other health care consumption, patient costs (i.e., informal care, travel expenses to any health care-related appointments) and productivity losses.

For the thyroid-related health care consumption, the real volumes of health care consumption were extracted from individual patient files. The extracted data included all thyroid surgeries and associated hospital admission days, additional diagnostics, surgeries, and hospital admission days following surgical complications, all outpatient clinic visits and diagnostics related to the diagnosis and treatment of the index nodule, additional diagnostics and consultations with other physicians related to [18F]FDG-PET/CT incidental findings, and use of thyroid-related medication.

Data concerning other, non-thyroid-related health care consumption and productivity losses were patient-reported at 0 (baseline), 3, 6 and 12 months, using the iMTA Medical Consumption Questionnaire (iMCQ), the iMTA Productivity Costs Questionnaire (iPCQ) questionnaire, respectively [5, 6]. To estimate health care consumption and productivity for the periods that were not covered by a cost questionnaire (by design of the trial and by the respective 3-month and 4-week recall periods of the iMCQ and iPCQ questionnaires), missing data were interpolated from the closest available questionnaires from the same patient.

Health care was valued using reference prices and the 2019 reimbursement rates of the Dutch System of Diagnosis-Treatment Combinations, where appropriate and available [7, 8]. Costs for any complications of the diagnostic surgeries or completion thyroidectomies (i.e., admission days for prolonged hospitalization or re-admission, diagnostics and/or additional surgeries) were estimated using the Dutch reimbursement rates. Costs of productivity losses were assessed using the friction cost method and reference prices for productivity [8]. Travel expenses were estimated at €0.19 per kilometer [8]. We estimated all costs from a Dutch societal perspective in Euros. All prices were indexed to 1 December 2019 using the Dutch consumer price index [9].
All costs related to the [18F]FDG-PET/CT, including procedure costs, costs for additional healthcare consumption for incidental [18F]FDG-PET/CT findings, travel expenses and other reported patient costs, were only taken into account for patients in the [18F]FDG-PET/CT-driven group.
The total societal costs were estimated as the sum of medical costs for all thyroid nodule-related and all other health care consumption, patient costs (i.e., travel expenses and informal care), and costs from productivity losses.
The differences in societal costs between the [18F]FDG-PET/CT-driven and diagnostic surgery group over the first year were calculated using independent two-sample t-tests with unequal variances. A generalized linear model (GLM) was used to adjust for the stratifying factors and malignancy rate; the adjusted p-value, corrected mean difference and 95% confidence interval are presented. As the postoperative treatment of the individual patients was based on the *local* histopathological diagnosis and likely influenced the costs, this diagnosis was included in the GLM.

Supplementary Table 4: Types of diagnostic surgery and histopathological diagnoses in patients who underwent diagnostic surgery during study follow-up.

	[18F]FDG-PET/CT-driven group (n = 91)	diagnostic surgery group (n = 41)	p
Diagnostic surgery	**n = 66**	**n = 40**	
Hemithyroidectomy	58 (88%)	38 (95%)	0.31[b]
Other	8 (12%)	2 (5%)	
Hemithyroidectomy + nodulectomy	0 (0%)	2 (5%)	
Isthmus resection	3 (5%)	0 (0%)	
Total thyroidectomy	5 (8%)	0 (0%)	
Histopathological diagnosis			
Malignant*	20 (22%)	5 (12%)	0.18[a]
PTC	4	2	
FVPTC	2	2	
FTC	5	1	
HCC	5		
DTC not otherwise specified	1		
PDTC	1		
MTC	2		
Borderline*	8 (9%)	1 (2%)	0.27[b]
NIFTP	5		
FT-UMP, Hürthle cell type	2	1	
Paraganglioma	1	0	
Benign*	38 (42%)	34 (83%)	<0.001[a]
Follicular adenoma	11	18	
Hürthle cell adenoma	9	5	
Hyperplastic nodule	18	11	
Additional incidental microcarcinoma	9 (14%)	3 (8%)	0.53[b]
in patients with benign histopathology	6	3	

DTC=differentiated thyroid carcinoma. FTC=follicular thyroid carcinoma. FT-UMP=follicular tumour of uncertain malignant potential. FVPTC=follicular variant PTC. HCC=Hürthle cell carcinoma. MTC=medullary thyroid carcinoma. PDTC=poorly differentiated thyroid carcinoma. PTC=papillary thyroid carcinoma. NIFTP=non-invasive follicular thyroid neoplasm with papillary-like nuclear features.
*: percentages and between-group comparisons are calculated as the ratio between patients with a malignant, borderline or benign histopathological diagnosis and all patients in the respective study group (n=91 in the [18F]FDG-PET/CT-driven group and n=41 in the diagnostic surgery group).
[a]: Pearson's chi-squared test. [b]: Fisher's exact test.

Supplementary Table 5: Discordant histopathology diagnoses

Blinded central review of the histopathology was discordant and changed the final classification in six of 132 index nodules (6%).

no.	[18F]FDG-PET/CT result	Local histopathology diagnosis	Initial classification	Central review of histopathology	Final classification
1	positive	Malignant (eFVPTC)*	True-positive	Borderline (NIFTP)*	True-positive
2	positive	Malignant (PTC)	True-positive	Benign (follicular adenoma)	False-positive
3	negative	Borderline (FT-UMP)	True-negative	Malignant (FVPTC)	False-negative
4	negative	Malignant (FVPTC)	False-negative	Benign (follicular adenoma)	True-negative
5	positive	Borderline (NIFTP)	True-positive	Benign (follicular adenoma)	False-positive
6	positive	Malignant (Hürthle cell carcinoma)	True-positive	Borderline (FT-UMP, oncocytic type)	False-positive

*: initial histopathological diagnosis was made prior to the global introduction and acceptance of the NIFTP diagnosis. eFVPTC=encapsulated FVPTC. FT-UMP=follicular tumour of uncertain malignant potential. FVPTC=follicular variant PTC. PTC=papillary thyroid carcinoma. NIFTP=non-invasive follicular thyroid neoplasm with papillary-like nuclear features.

Subgroup analysis for nodules >10 mm

Supplementary Table 6: FDG-PET/CT parameters in nodules >10 mm (n=128).

	n	[18F]FDG-PET/CT-driven group (n = 89)	diagnostic surgery group (n = 39)	p
**[18F]FDG-PET/CT**				
[18F]FDG-positive	128	65 (73%)	26 (67%)	0.47[a]
Median SUV_{max} of the nodule, g/cm^3 (IQR)	128	4.0 (2.8-10.7)	4.0 (2.5-8.3)	0.43[b]
Median SUV_{peak} of the nodule, g/cm^3 (IQR)	128	3.5 (2.3-8.4)	3.3 (2.1-6.1)	0.35[b]
Median SUV_{max} of thyroid background, g/cm^3 (IQR)	128	1.9 (1.7-2.4)	2.0 (1.7-2.5)	0.19[b]
Median SUV_{max} ratio (IQR)	128	2.4 (1.4-6.2)	1.8 (1.1-4.1)	0.16[b]
Median SUV_{peak} ratio (IQR)	128	1.9 (1.1-4.8)	1.5 (0.9-2.8)	0.11[b]

IQR=interquartile range. SUV=standardized uptake value. [a]: Pearson's chi-squared test. [b]: Mann-Whitney U test.

Supplementary Table 7: Therapeutic yield after one year of follow-up in nodules >10 mm (n=128).

	[18F]FDG-PET/CT-driven group (n=89)		diagnostic surgery group (n=39)				
	**n**	**% (95% CI)**	**n**	**% (95% CI)**	p[a]	adjusted p[b]	adjusted OR (95% CI)[b]
**Beneficial management**	**51 / 89**	**57% (46%-68%)**	**7 / 39**	**18% (8%-34%)**	***<0.001[a]***	***<0.001[b]***	***6.2 (2.4-16.1)[b]***
Surgery for malignant/borderline nodule	28 / 89	31% (22%-42%)	6 / 39	15% (6%-31%)	0.06[a]	0.09[b]	2.4 (0.9-6.8)[b]
Surveillance for benign nodule	23 / 89	26% (17%-36%)	1 / 39	3% (0%-13%)	*0.002[a]*	*<0.001[b]*	*15.2 (1.9-120.2)[b]*
**Unbeneficial management**	**38 / 89**	**43% (32%-54%)**	**32 / 39**	**82% (66%-92%)**	***<0.001[a]***	***<0.001[b]***	***0.2 (0.1-0.4)[b]***
Surgery for benign nodule	38 / 89	43% (32%-54%)	32 / 39	82% (66%-92%)	*<0.001[a]*	*<0.001[b]*	*0.2 (0.1-0.4)[b]*
Surveillance for malignant/borderline nodule	0 / 89	0% (0%-4%)	0 / 39	0% (0%-9%)	n.a.	n.a.	n.a.
**Avoided surgery in benign nodules**	**23 / 61**	**38% (26%-51%)**	**1 / 33**	**3% (0%-16%)**	***<0.001[a]***	***0.003[b]***	***25.1 (3.0-211.6)[b]***

CI=confidence interval. n.a.=not applicable. OR=odds ratio.

[a]: Pearson's chi-squared test. [b]: binary logistic regression to adjust for stratifying variables.

Supplementary Table 8: Diagnostic accuracy parameters for nodules >10 mm (n=128)[a].

n	TP	FP	TN	FN	sensitivity (95% CI)	specificity (95% CI)	NPV (95% CI)	PPV (95% CI)	benign call rate (95% CI)
128	32	59	35	2	94.1% (80.3%-99.3%)	37.2% (27.5%-47.8%)	94.6% (81.8%–99.3%)	35.2% (25.4%–45.9%)	28.9% (21.2%–37.6%)

CI=confidence interval. FN=false-negative. FP=false-positive. PPV=positive predictive value. NPV=negative predictive value. TN=true-negative. TP=true-positive.
[a]: whole-group analysis was performed to estimate diagnostic accuracy parameters.

Supplementary Table 9: Secondary outcomes for nodules >10 mm (n=128).

	[^{18}F]FDG-PET/CT-driven group (n = 89)	diagnostic surgery group (n = 39)	p	adjusted p[c]	adjusted OR (95% CI)[c]
**Surgical complications**	13 (15%)	10 (26%)*	0.13[a]	0.17[c]	0.5 (0.2-1.3)[c]
in benign nodules (n=94)	9 (15%)	9 (27%)	0.14[a]	0.24[c]	0.5 (0.2-1.5)[c]
in malignant/borderline nodules (n=34)	4 (14%)	1 (17%)	1[b]	0.75[c]	1.6 (0.1-30.7)[c]
Type of complication following diagnostic surgery					
Wound infection	1 (1%)	1 (3%)	0.52[b]	0.59[c]	0.5 (0.0-8.0)[c]
Hematoma with re-exploration surgery	1 (1%)	1 (3%)*	0.52[b]	0.55[c]	0.4 (0.0-8.1)[c]
Seroma	1 (1%)	1 (3%)	0.52[b]	0.62[c]	0.5 (0.0-8.7)[c]
Recurrent nerve paralysis	2 (2%)	0 (0%)	1[b]	1[c]	2.5E+7 (0-∞)[c]
Hypothyroidism following partial thyroidectomy**	5 (6%)***	7 (18%)*	0.051[b]	0.07[c]	0.3 (0.1-1.1)[c]
Hypoparathyroidism, transient	3 (3%)****	1 (3%)	1[b]	0.85[c]	1.3 (0.1-13.2)[c]
**Survival after 1 year**	89 (100%)	39 (100%)	n.a.	n.a.	n.a.

CI=confidence interval. N.a.=not applicable. N.s.=not specified. OR=odds ratio.
*: two complications (hematoma and hypothyroidism) occurred in one patient.
**: Hypothyroidism due to partial thyroidectomy included patients who had new levothyroxine-dependent hypothyroidism following a partial thyroidectomy procedure (i.e., hemithyroidectomy and/or isthmus resection).
***: initial total thyroidectomies (n=5) are excluded from the denominator.
****: transient hypoparathyroidism only occurred following initial total thyroidectomy.
[a]: Pearson's chi-squared test. [b]: Fisher's exact test. [c]: binary logistic regression to adjust for stratifying variables.

Supplementary Table 10: Secondary outcomes for nodules >10 mm (n=128): HRQoL and societal costs.

	**[18F]FDG-PET/CT-driven group (n=91)**	**diagnostic surgery group (n=41)**	p	mean difference (95% CI)
**HRQoL**				
Mean one-year QALYs from EQ-5D-5L (95% CI)	0.791 (0.746-0.835)	0.718 (0.652-0.785)	0.10[a]	0.072 (-0.014-+0.158)[a]
Adjusted mean one-year QALYs from EQ-5D-5L (95% CI)	0.791 (0.751-0.832)	0.717 (0.639-0.794)	0.09[b]	0.075 (-0.012-+0.161)[b]
**Societal costs**				
Mean one-year societal costs (95% CI)	€15,800 (+€12,900-+€18,800)	€19,600 (+€14,900-+€24,200)	0.22[a]	-€3,700 (-€9,700-+€2,200)[a]
Adjusted mean one-year societal costs (95% CI)	€15,100 (+€12,800-€17,300)	€21,300 (+€16,300-€26,300)	*0.02[b]*	-€6,200 (-€11,500--€900)[b]

CI=confidence interval. HRQoL=health-related quality of life. QALYs=quality-adjusted life years.

[a]: independent samples t-test with unequal variances. [b]: generalized linear model, adjusted analysis for stratifying variables and malignancy/borderline rate based on the local histopathological diagnosis.

Supplementary Table 11: Therapeutic yield after one year of follow-up in AUS/FLUS, FN/SFN and HCN/SHCN nodules >10 mm.

	[^{18}F]FDG-PET/CT-driven group		diagnostic surgery group		
**non-Hürthle cell nodules, AUS/FLUS + FN/SFN (n=97)**	**n=66**	**% (95% CI)**	**n=31**	**% (95% CI)**	p
**Beneficial management**	**41 / 66**	**62% (49%-74%)**	**5 / 31**	**16% (5%-34%)**	***<0.001***[a]
Surgery for malignant/borderline nodule	20 / 66	30% (20%-43%)	5 / 31	16% (5%-34%)	0.14[a]
Surveillance for benign nodule	21 / 66	32% (21%-44%)	0 / 31	0% (0%-11%)	*<0.001*[a]
**Unbeneficial management**	**25 / 66**	**38% (26%-51%)**	**26 / 31**	**84% (66%-95%)**	***<0.001***[a]
Surgery for benign nodule	25 / 66	38% (26%-51%)	26 / 31	84% (66%-95%)	*<0.001*[a]
Surveillance for malignant/borderline nodule	0 / 66	0 % (0%-5%)	0 / 31	0% (0%-11%)	n.a.
**Avoided surgery in benign nodules**	**21 / 46**	**46% (31%-61%)**	**0 / 26**	**0% (0%-13%)**	***<0.001***[a]
**AUS/FLUS (n=58)**	**n=38**	**% (95% CI)**	**n=20**	**% (95% CI)**	p
**Beneficial management**	**22 / 38**	**58% (41%-74%)**	**1 / 20**	**5% (0%-25%)**	***<0.001***[a]
Surgery for malignant/borderline nodule	9 / 38	24% (11%-40%)	1 / 20	5% (0%-25%)	0.14[b]
Surveillance for benign nodule	13 / 38	34% (20%-51%)	0 / 20	0% (0%-17%)	*0.002*[b]
**Unbeneficial management**	**16 / 38**	**42% (26%-59%)**	**19 / 20**	**95% (75%-100%)**	***<0.001***[a]
Surgery for benign nodule	16 / 38	42% (26%-59%)	19 / 20	95% (75%-100%)	*<0.001*[a]
Surveillance for malignant/borderline nodule	0 / 38	0% (0%-9%)	0 / 20	0% (0%-17%)	n.a.
**Avoided surgery in benign nodules**	**13 / 29**	**45% (26%-64%)**	**0 / 19**	**0% (0%-18%)**	***<0.001***[a]
**FN/SFN (n=39)**	**n = 28**	**% (95% CI)**	**n = 11**	**% (95% CI)**	p
**Beneficial management**	**19 / 28**	**68% (48%-84%)**	**4 / 11**	**36% (11%-69%)**	**0.15**[b]
Surgery for malignant/borderline nodule	11 / 28	39% (22%-59%)	4 / 11	36% (11%-69%)	1b
Surveillance for benign nodule	8 / 28	29% (13%-49%)	0 / 11	0% (0%-28%)	0.08[b]
**Unbeneficial management**	**9 / 28**	**32% (16%-52%)**	**7 / 11**	**64% (31%-89%)**	**0.15**[b]
Surgery for benign nodule	9 / 28	32% (16%-52%)	7 / 11	64% (31%-89%)	0.15[b]
Surveillance for malignant/borderline nodule	0 / 28	0% (0%-12%)	0 / 11	0% (0%-28%)	n.a.
**Avoided surgery in benign nodules**	**8 / 17**	**47% (23%-72%)**	**0 / 7**	**0% (0%-41%)**	***0.05***[b]
**Hürthle cell nodules, HCN/SHCN (n=31)**	**n=23**	**% (95% CI)**	**n=8**	**% (95% CI)**	p
**Beneficial management**	**10 / 23**	**43% (23%-66%)**	**2 / 8**	**25% (3%-65%)**	**0.43**[b]
Surgery for malignant/borderline nodule	8 / 23	35% (16%-57%)	1 / 8	13% (0%-53%)	0.38[b]
Surveillance for benign nodule	2 / 23	9% (1%-28%)	1 / 8	13% (0%-53%)	1[b]
**Unbeneficial management**	**13 / 23**	**57% (34%-77%)**	**6 / 8**	**75% (35%-97%)**	**0.43**[b]
Surgery for benign nodule	13 / 23	57% (34%-77%)	6 / 8	75% (35%-97%)	0.43[b]
Surveillance for malignant/borderline nodule	0 / 23	0% (0%-15%)	0 / 8	0% (0%-37%)	n.a.
**Avoided surgery in benign nodules**	**2 / 15**	**13% (2%-40%)**	**1 / 7**	**14% (0%-58%)**	**1**[b]

AUS/FLUS=atypia of undetermined significance or follicular lesions of undetermined significance. CI=confidence interval. FN/SFN=(suspicious for a) follicular neoplasm. HCN/SHCN=(suspicious for a) Hürthle cell neoplasm. N.a.=not applicable. [a]: Pearson's chi-squared test. [b]: Fisher's exact test.

Subgroup analysis: Differentiation between strictly benign and malignant nodules

Supplementary Table 12: Benign and malignant diagnosis per group

	n	[18F]FDG-PET/CT-driven group n = 91	diagnostic surgery group n = 41	p
**Diagnosis**	132			
Malignant		20 (22%)	5 (12%)	0.18
Benign		71 (78%)	36 (88%)	

Supplementary Table 13: therapeutic yield after one year of follow-up for differentiation between strictly benign and malignant nodules.

	[18F]FDG-PET/CT-driven group n=91		diagnostic surgery group n=41		
	**n**	**% (95% CI)**	**n**	**% (95% CI)**	p
**Beneficial management**	**45 / 91**	**49% (39%-60%)**	**6 / 41**	**15% (6%-29%)**	***<0.001[a]***
Surgery for malignant nodule	20 / 91	22% (14%-32%)	5 / 41	12% (4%-26%)	0.18[a]
Surveillance for benign nodule	25 / 91	27% (19%-38%)	1 / 41	2% (0%-13%)	*0.001[a]*
**Unbeneficial management**	**46 / 91**	**51% (40%-61%**	**35 / 41**	**85% (71%-94%)**	***<0.001[a]***
Surgery for benign nodule	46 / 91	51% (40%-61%	35 / 41	85% (71%-94%)	*<0.001[a]*
Surveillance for malignant nodule	0 / 91	0% (0%-4%)	0 / 41	0% (0%-9%)	n.a.
**Avoided surgery in benign nodules**	**25 / 71**	**35% (24%-47%)**	**1 / 36**	**3% (0%-15%)**	***<0.001[a]***

CI=confidence interval. n.a.=not applicable.
[a]: Pearson's chi-squared test.

Supplementary Table 14: therapeutic yield after one year of follow-up in AUS/FLUS, FN/SFN and HCN/SHCN for differentiation between strictly benign and malignant nodules.

	**[^{18}F]FDG-PET/CT-driven group (n=91)**		**diagnostic surgery group (n=41)**		
**non-Hürthle cell nodules, AUS/FLUS + FN/SFN (n=101)**	**n=68**	**% (95% CI)**	**n=33**	**% (95% CI)**	p
**Beneficial management**	**37 / 68**	**54% (42%-67%)**	**5 / 33**	**15% (5%-32%)**	***<0.001***[a]
Surgery for malignant nodule	14 / 68	21% (12%-32%)	5 / 33	15% (5%-32%)	0.51[a]
Surveillance for benign nodule	23 / 68	34% (23%-46%)	0 / 33	0% (0%-11%)	*<0.001*[a]
**Unbeneficial management**	**31 / 68**	**46% (33%-58%)**	**28 / 33**	**85% (68%-95%)**	***<0.001***[a]
Surgery for benign nodule	31 / 68	46% (33%-58%)	28 / 33	85% (68%-95%)	*<0.001*[a]
Surveillance for malignant nodule	0 / 68	0 % (0%-5%)	0 / 33	0% (0%-11%)	n.a.
**Avoided surgery in benign nodules**	**23 / 54**	**43% (29%-57%)**	**0 / 28**	**0% (0%-12%)**	***<0.001***[a]
**AUS/FLUS (n=60)**	**n=40**	**% (95% CI)**	**n=20**	**% (95% CI)**	p
**Beneficial management**	**20 / 40**	**50% (34%-66%)**	**1 / 20**	**5% (0%-25%)**	***<0.001***[a]
Surgery for malignant nodule	5 / 40	13% (4%-27%)	1 / 20	5% (0%-25%)	0.65[b]
Surveillance for benign nodule	15 / 40	38% (23%-54%)	0 / 20	0% (0%-17%)	*0.002*[a]
**Unbeneficial management**	**20 / 40**	**50% (34%-66%)**	**19 / 20**	**95% (75%-100%)**	***0.001***[a]
Surgery for benign nodule	20 / 40	50% (34%-66%)	19 / 20	95% (75%-100%)	*0.001*[a]
Surveillance for malignant nodule	0 / 40	0% (0%-9%)	0 / 20	0% (0%-17%)	n.a.
**Avoided surgery in benign nodules**	**15 / 35**	**43% (26%-61%)**	**0 / 19**	**0% (0%-18%)**	***<0.001***[a]
**FN/SFN (n=41)**	**n = 28**	**% (95% CI)**	**n = 13**	**% (95% CI)**	p
**Beneficial management**	**17 / 28**	**61% (41%-78%)**	**4 / 13**	**31% (9%-61%)**	**0.07**[a]
Surgery for malignant nodule	9 / 28	32% (16%-52%)	4 / 13	31% (9%-61%)	1[b]
Surveillance for benign nodule	8 / 28	29% (13%-49%)	0 / 13	0% (0%-25%)	*0.04*[b]
**Unbeneficial management**	**11 / 28**	**39% (22%-59%)**	**9 / 13**	**69% (39%-91%)**	**0.07**[a]
Surgery for benign nodule	11 / 28	39% (22%-59%)	9 / 13	69% (39%-91%)	0.07[a]
Surveillance for malignant nodule	0 / 28	0% (0%-12%)	0 / 13	0% (0%-25%)	n.a.
**Avoided surgery in benign nodules**	**8 / 19**	**42% (20%-67%)**	**0 / 9**	**0% (0%-34%)**	***0.03***[b]
**Hürthle cell nodules, HCN/SHCN (n=31)**	**n=23**	**% (95% CI)**	**n=8**	**% (95% CI)**	p
**Beneficial management**	**8 / 23**	**35% (16%-57%)**	**1 / 8**	**13% (0%-53%)**	**0.38**
Surgery for malignant nodule	6 / 23	26% (10%-48%)	0 / 8	0% (0%-37%)	0.30[b]
Surveillance for benign nodule	2 / 23	9% (1%-28%)	1 / 8	13% (0%-53%)	1[b]
**Unbeneficial management**	**15 / 23**	**65% (43%-84%)**	**7 / 8**	**88% (47%-100%)**	**0.38**[b]
Surgery for benign nodule	15 / 23	65% (43%-84%)	7 / 8	88% (47%-100%)	0.38[b]
Surveillance for malignant nodule	0 / 23	0% (0%-15%)	0 / 8	0% (0%-37%)	n.a.
**Avoided surgery in benign nodules**	**2 / 17**	**12% (1%-36%)**	**1 / 8**	**13% (0%-53%)**	**1**[b]

AUS/FLUS=atypia of undetermined significance or follicular lesions of undetermined significance. CI=confidence interval. FN/SFN=(suspicious for a) follicular neoplasm. HCN/SHCN=(suspicious for a) Hürthle cell neoplasm. N.a.=not applicable. [a]: Pearson's chi-squared test. [b]: Fisher's exact test.

Nodules with false-negative [^{18}F]FDG-PET/CT

Two of 132 (1.5%) [^{18}F]FDG-PET/CT scans were considered false negative.

Case 1 was a 15 mm left-sided solitary nodule. On [^{18}F]FDG-PET/CT, it was a well-defined, smooth, and hypodense nodule with [^{18}F]FDG-uptake that was similar to its background upon visual assessment (Supplementary Figure 3). As such, it was defined as [^{18}F]FDG-negative. SUV_{max} and SUV_{peak} values for this nodule were 2.5 g/cm^3 and 2.0 g/cm^3, respectively, as compared to a SUV_{max} of 2.4 g/cm^3 for the background of normal thyroid tissue.
On histopathology, the neoplasm composed mainly of spindle cells and small areas with morphological characteristics of PTC or follicular adenoma. It showed focal positive staining for Galectin-3 and HBME-1 and diffuse strong immunoreactivity for TTF-1 and PAX-8. On next-generation sequencing, a point mutation in NRAS was detected. Differential diagnosis of this nodule included PTC or follicular adenoma with uncommon spindle cell metaplasia. This nodule was ultimately classified as a spindle cell PTC (TNM pT1b), after extensive assessment of the histopathology by dedicated thyroid pathologists from the University Medical Centre Groningen (Groningen, the Netherlands) and Radboud university medical centre (Nijmegen, the Netherlands), and including consultation with a pathologist of the University of Pittsburgh Medical Centre (Pittsburgh, PA, USA) (Supplementary Figures 4 and 5).

Case 2 was a 32 mm right-sided solitary nodule with a large cystic component. On [^{18}F]FDG-PET/CT, it was a well-defined, hypodense nodule surrounded by a relatively thick rim of solid tissue. The solid parts of the nodule, best assessed on the caudal side, were [^{18}F]FDG-negative compared to the background (Supplementary Figure 6). SUV_{max} and SUV_{peak} values for this nodule were 2.5 g/cm^3 and 2.0 g/cm^3, respectively, as compared to a SUV_{max} of 1.7 g/cm^3 for the background of normal thyroid tissue.
On histopathology, it was a predominantly cystic, non-invasive lesion with a follicular growth pattern (Supplementary Figures 7 and 8). The solid parts of this lesion surrounding the cyst had a maximum diameter of 8 mm. On microscopy, the lesion had heterogeneous follicular aspects (Supplementary figure 8b) but also areas with inconclusive papillary nuclear features (Supplementary figure 8c). The follicular epithelial cells had slightly enlarged nuclei, most of which were round but some of which were oval with some nuclear overlap and nuclear grooves. The lesion showed positive staining for Galectin-3 and CK-19 but also thyroid peroxidase (TPO). Based on these observations, the differential diagnosis included a well differentiated tumour of uncertain malignant potential (WDT-UMP) or a non-invasive follicular neoplasm with papillary-like nuclear features (NIFTP). Additional next generation sequencing was ultimately performed during central histopathological review, showing an ETV6-NTRK3 fusion. Based on this finding, the nodule was classified as an FVPTC (TNM pT2).

Most of the extensive assessments for both lesions were already performed as a part of the local histopathological assessment, as both lesions were morphologically difficult to diagnose. Only additional molecular diagnostics were performed in the context of the central histopathological review, to substantiate a differential diagnosis. Molecular diagnostics was performed in several difficult cases at the discretion of the central pathologists, who were unaware of the patients' [^{18}F]FDG-PET/CT results.

Supplementary Figure 3: [^{18}F]FDG-PET/CT images of case 1.
Transverse [^{18}F]FDG-PET/CT (**a**), coronal low-dose CT image (**b**), transverse PET image (**c**), and anterior PET maximum intensity projection (MIP) (**d**) of the 15 mm nodule.

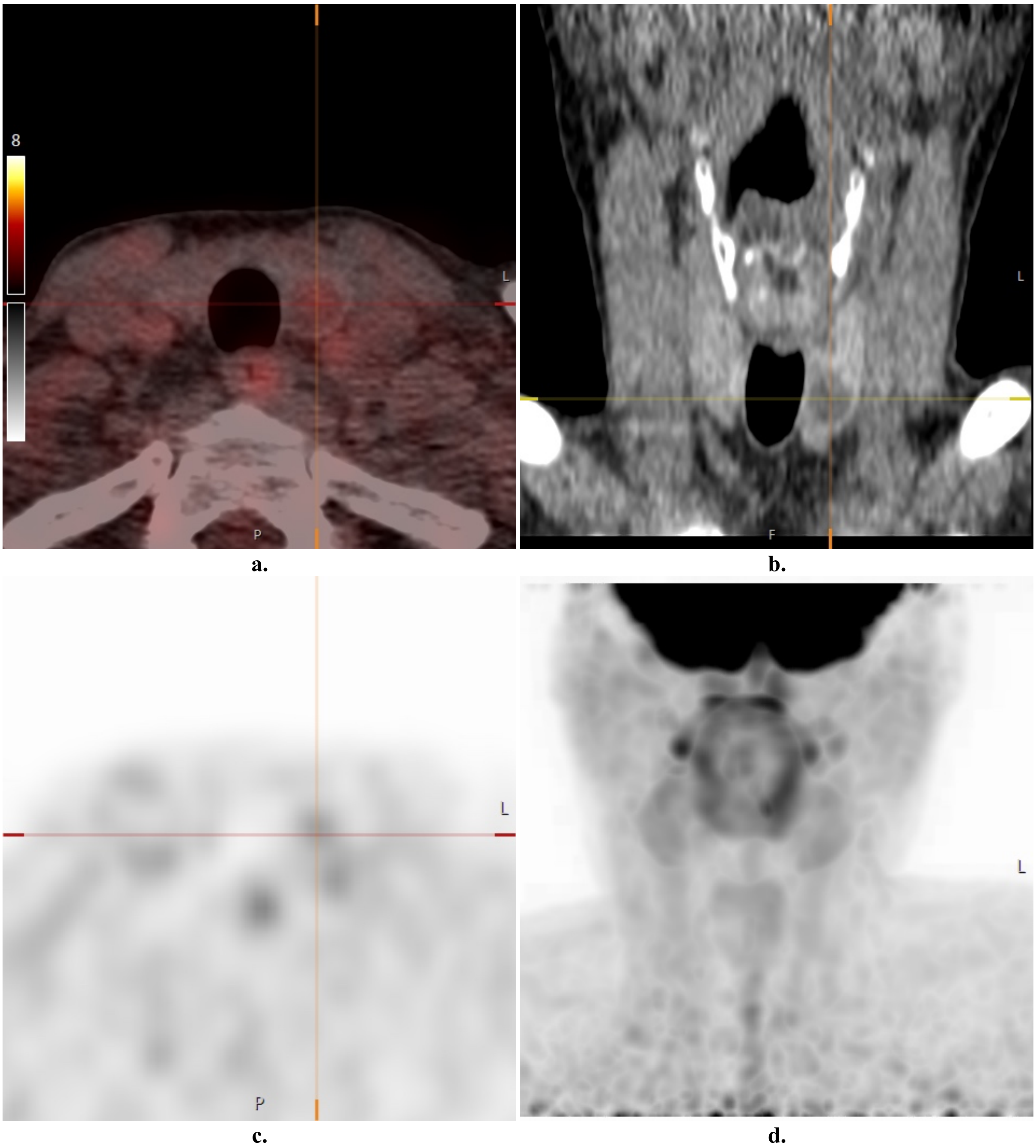

Supplementary Figure 4: Macroscopy images of case 1.

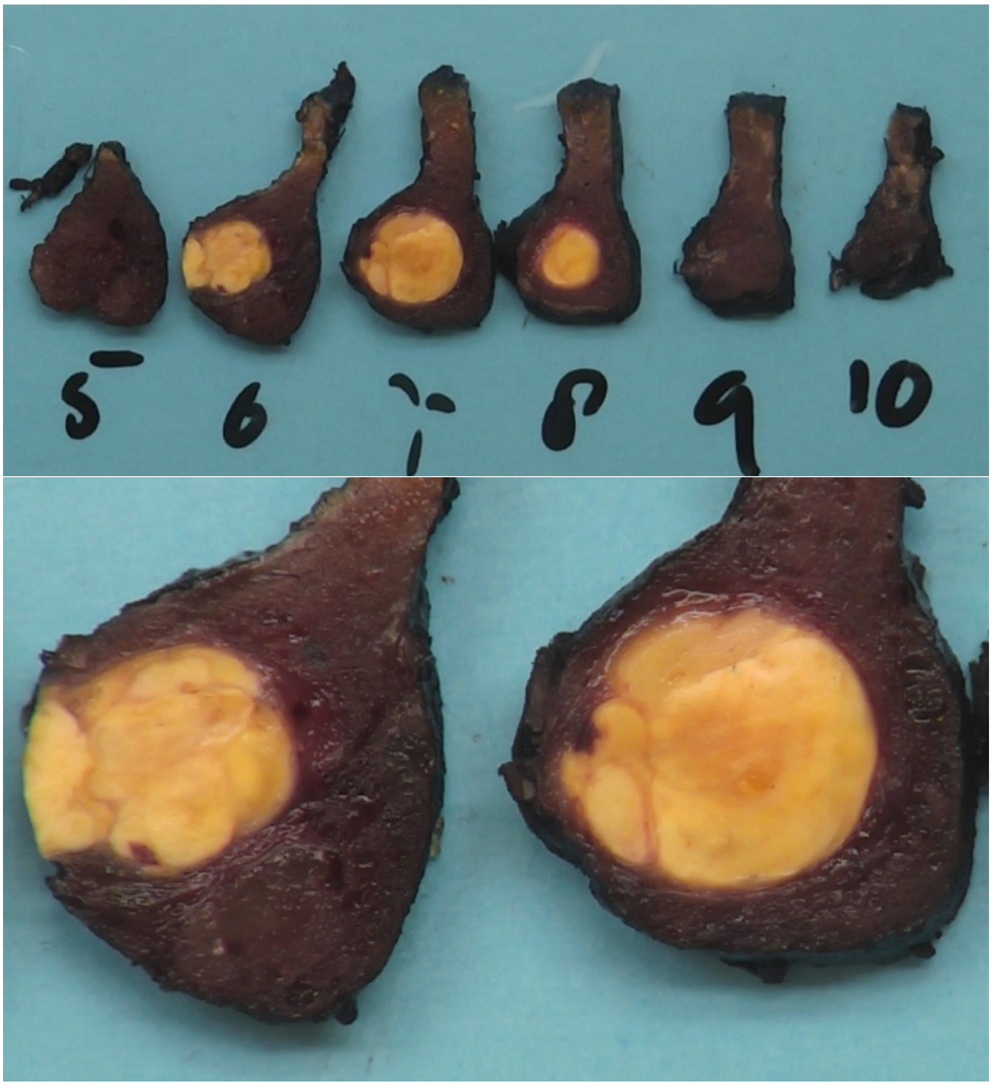

Supplementary Figure 5: Microscopy images of case 1.
Overview (**a**) and more detailed (**b** and **c**) images of H&E stained slides of the lesion.

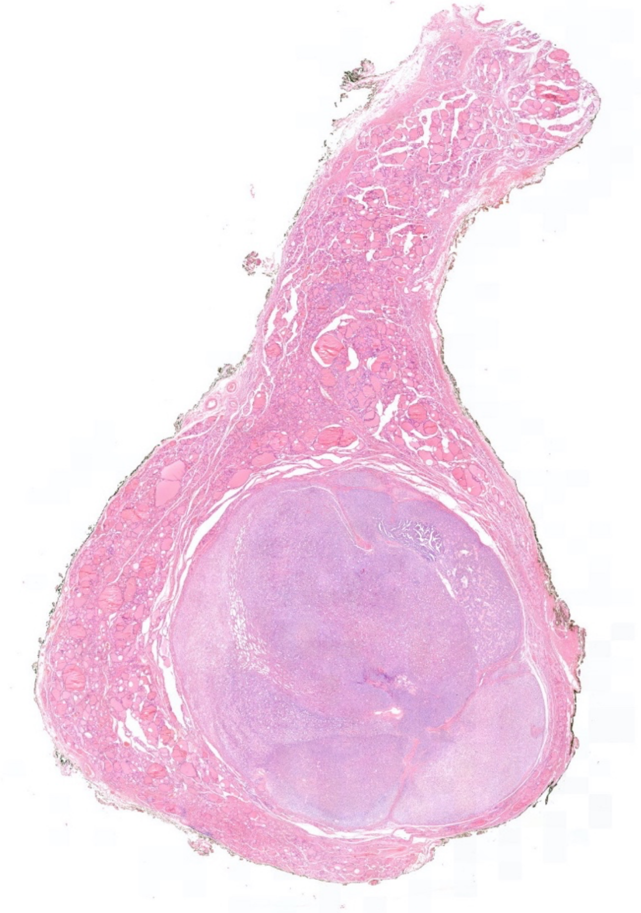

a. 0.4/40x

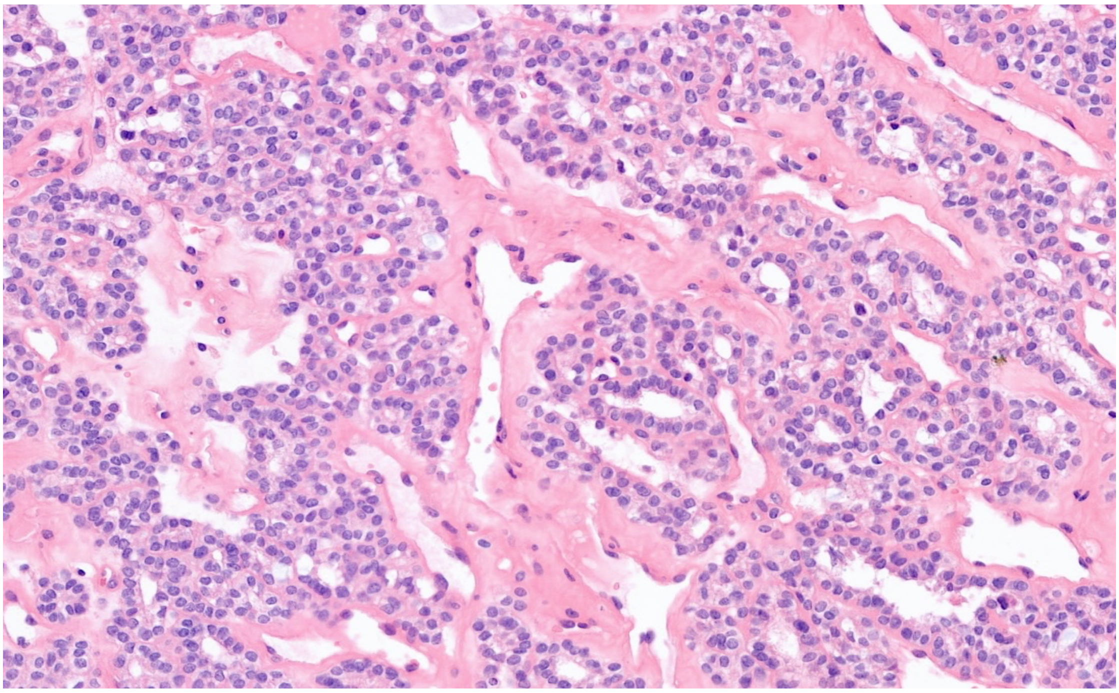

b. 20x

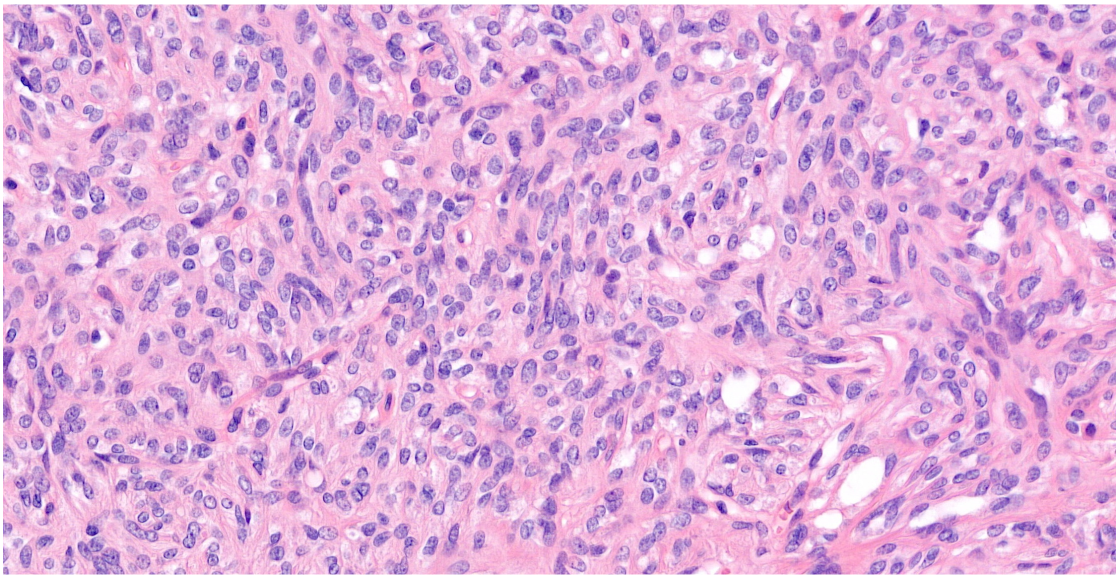
c. 40x

Supplementary Figure 6: [^{18}F]FDG-PET/CT images of case 2.
Coronal [^{18}F]FDG-PET/CT image (**a**), sagittal [^{18}F]FDG-PET/CT image (**b**), coronal PET image (**c**), and anterior PET maximum intensity projection (MIP) (**d**) of the 32 mm nodule.

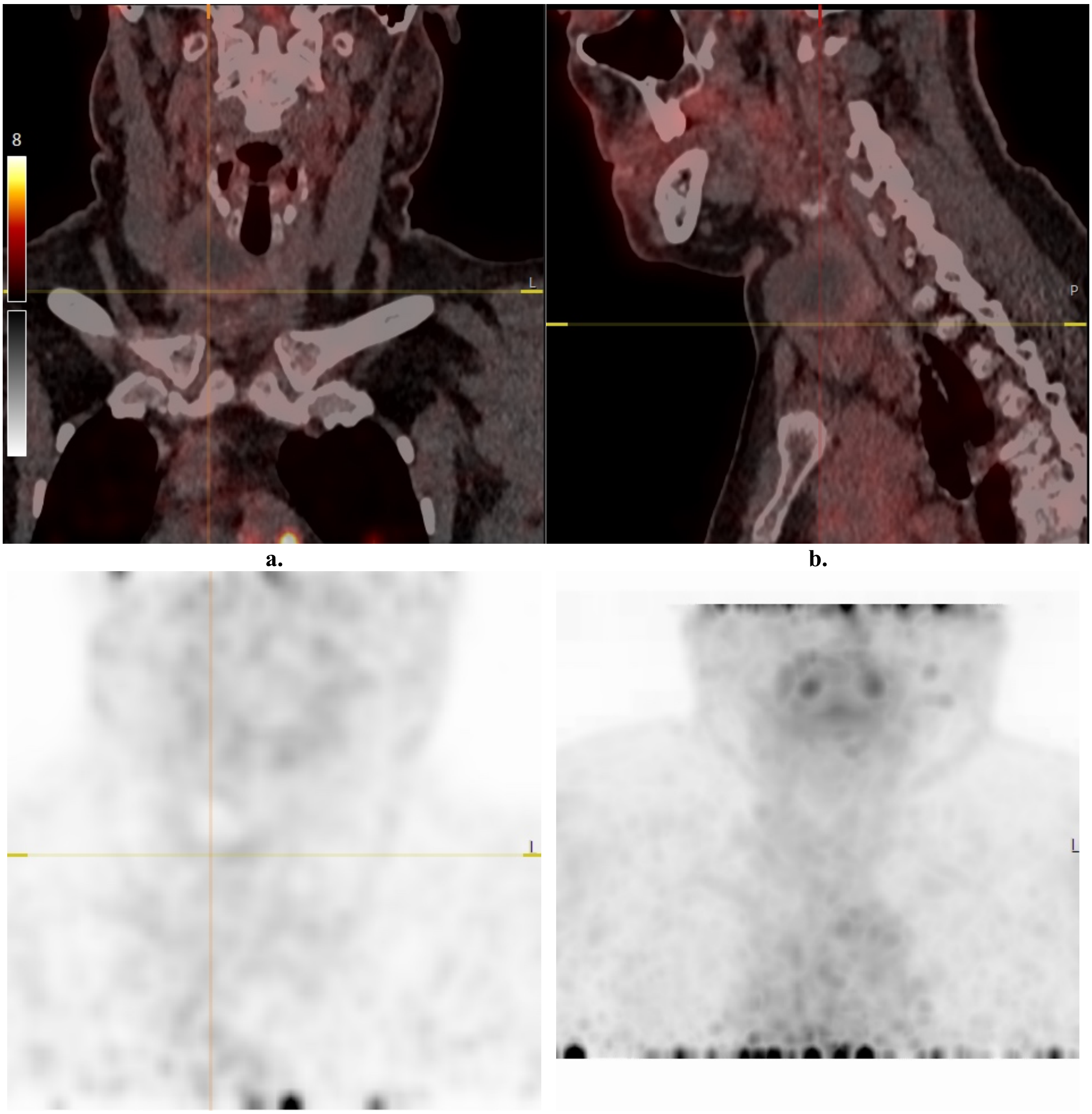

a. **b.** **c.** **d.**

Supplementary Figure 7: Macroscopy image of case 2.

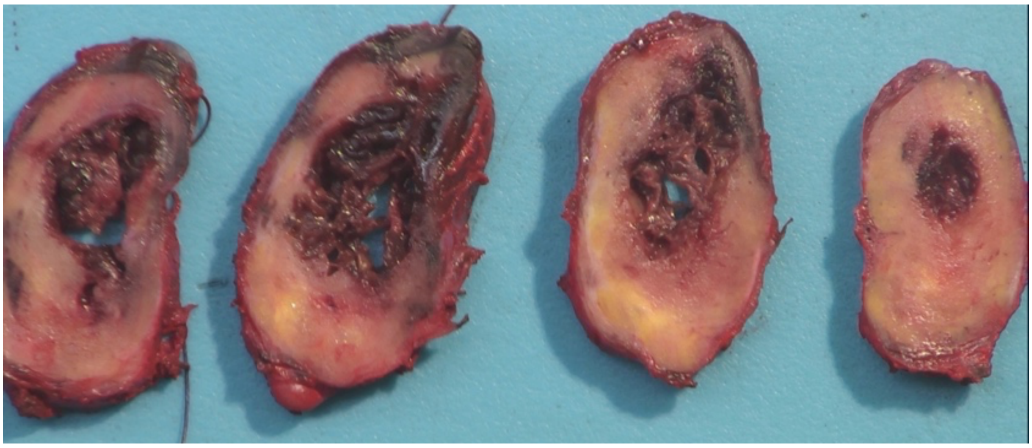

Supplementary Figure 8: Microscopy images of case 2.
Overview (**a**) and more detailed (**b** and **c**) images of H&E stained slides of the lesion.

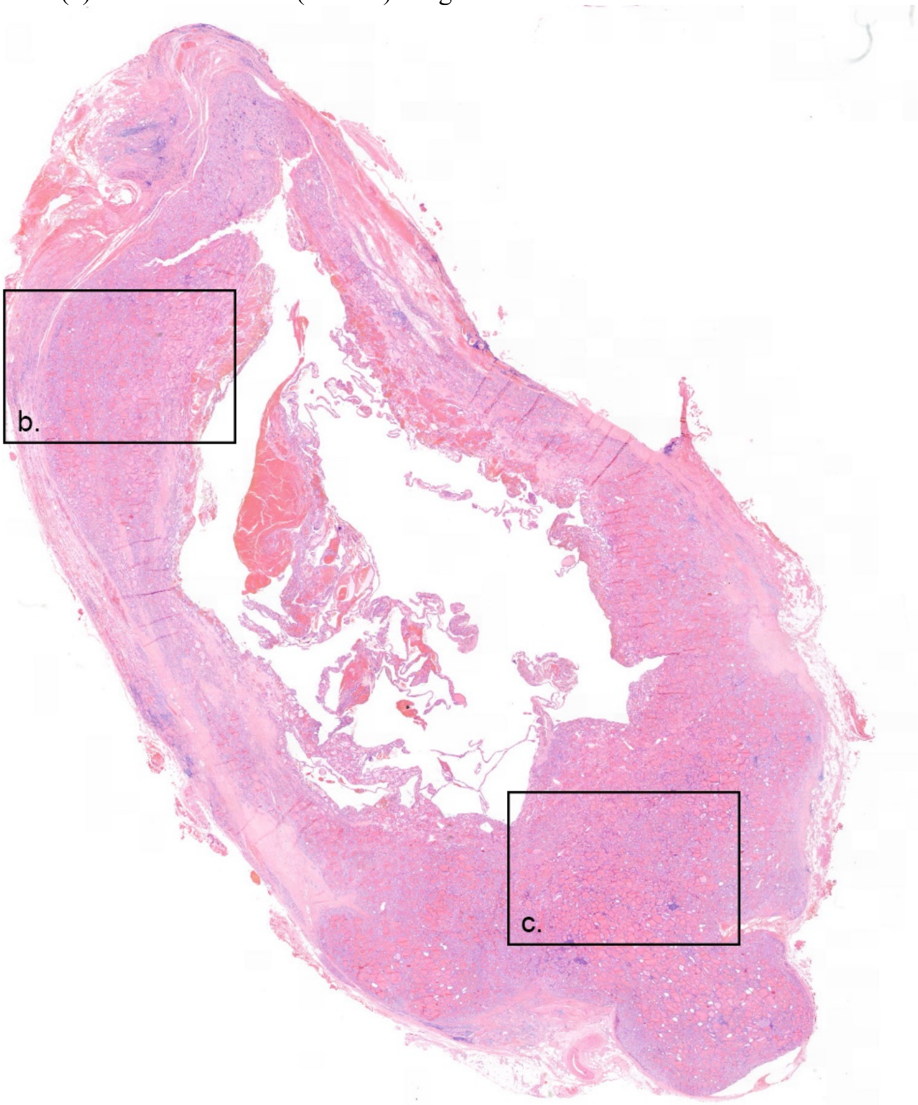

a. 0.4/40x

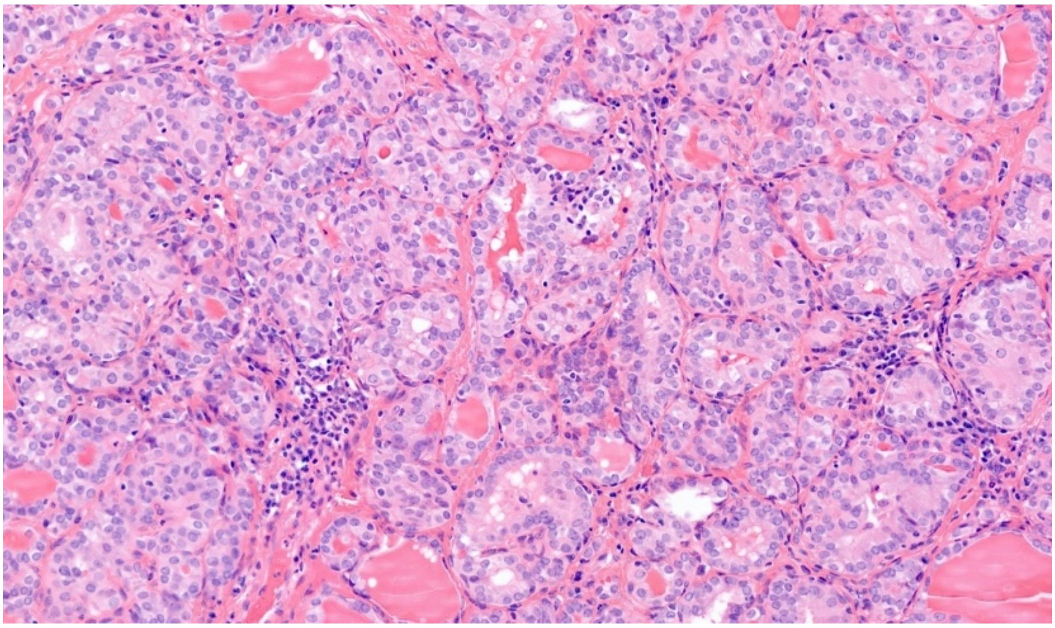
b. 13/40x.

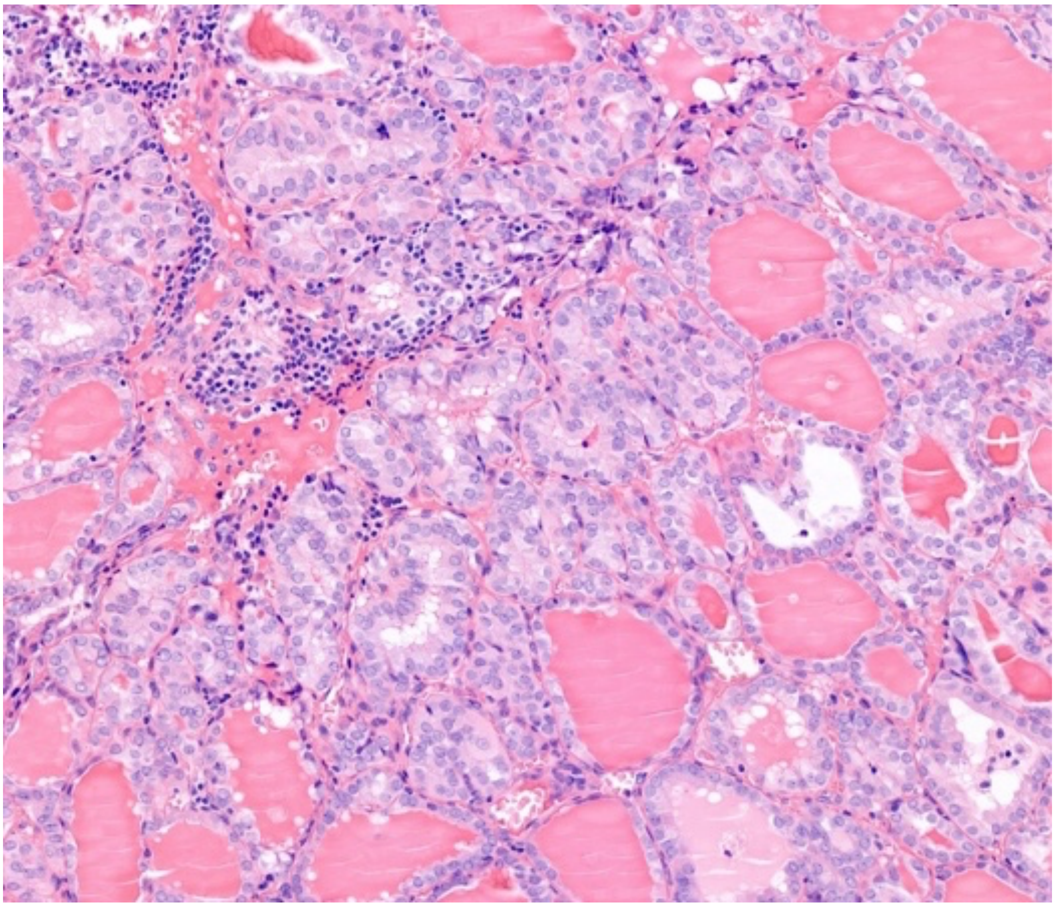
c. 13/40x.

Incidental [^{18}F]FDG-PET/CT findings

On the partial-body [^{18}F]FDG-PET/CT of the neck, 21 [^{18}F]FDG-positive thyroid incidentalomas were discovered in 19 (14%) patients, for which additional workup through FNAC was advised. FNAC was performed of 14 nodules (74%): cytology was nondiagnostic (Bethesda I) in three, benign (Bethesda II) in six, AUS/FLUS in three and FN/SFN in two nodules. Of these, six incidentalomas were surgically resected. One patient with repeated AUS/FLUS cytology and one patient with repeated FN/SFN cytology of the incidentaloma refused surgery. Altogether, 12 incidentalomas in 11 patients were resected: seven ipsilateral incidentalomas were removed through the scheduled hemithyroidectomy, of which two procedures were extended with a nodulectomy of the incidentaloma located in the isthmus; total thyroidectomy was performed in four patients. Ten incidentalomas were benign on histopathology (one follicular adenoma and nine hyperplastic nodules) and two were malignant (9.5%, 2/21): in one patient, the incidentaloma was part of the multifocal poorly differentiated carcinoma; in the other, the incidentaloma was a 6 mm follicular variant of PTC (FVPTC). Altogether, in three of 22 (14%) patients the diagnostic and/or therapeutic consequences of the incidental [^{18}F]FDG-PET/CT findings were justified and disease was detected: besides the two thyroid malignancies, this included one patient who was diagnosed with potential diabetes mellitus based on an increased fasting plasma glucose prior to the [^{18}F]FDG-PET/CT scan. In four (17%) patients, major therapeutic consequences were considered unbeneficial overtreatment. Although compliant with current guidelines, total thyroidectomy was performed instead of diagnostic hemithyroidectomy because Bethesda III, Bethesda IV, Bethesda VI, and Bethesda II cytology with an NRAS point mutation were respectively obtained the contralateral PET incidentaloma. On histopathology, all four contralateral nodules were benign.
In 15 (65%) patients, diagnostic and/or therapeutic consequences were considered unbeneficial yet minor (Supplementary Table 13).

Supplementary Table 15: Incidental [^{18}F]FDG-PET/CT findings (whole-group analysis).

	n = 132
**incidental [^{18}F]FDG-avid findings on [^{18}F]FDG-PET**	
with diagnostic and/or therapeutic consequence	20 (15%)
Thyroid incidentaloma	19 (14%)
ipsilateral	6 (5%)
contralateral	12 (9%)
bilateral	1 (1%)
FNAC*	13 (10%)
Bethesda I	2 (2%)
Bethesda II	6 (5%)
Bethesda III	3 (2%)
Bethesda IV	2 (2%)
Histopathology	11 (8%)
Benign	9 (7%)
unbeneficial extension of planned diagnostic surgery with isthmus nodulectomy	2 (25)
unbeneficial extension of planned diagnostic surgery to total thyroidectomy	4 (3%)*
Malignant	2 (2%)
extension of planned diagnostic surgery to total thyroidectomy	0 (0%)**
Ultrasound follow-up unchanged	2 (2%)
Cervical lymph node 9 mm, reactive on FNAC	1 (1%)
Axillary lymph nodes <10 mm, reactive on FNAC	1 (1%)
without diagnostic and/or therapeutic consequence	
Thyroiditis	9 (7%)
Cervical lymph nodes <6 mm, reactive based on history of sinusitis/pharyngitis	8 (6%)
Occipital lymph nodes 5 mm, reactive based on medical history	1 (1%)
Axillary lymph nodes <5 mm, reactive based on medical history	1 (1%)
Supraclavicular lymph nodes, non-enlarged, reactive based on medical history	1 (1%)
Paratracheal lymph nodes, non-enlarged, reactive based on history of respiratory tract infection	1 (1%)
Posttraumatic focal uptake in rib	1 (1%)
Posttraumatic uptake in humerus	1 (1%)
Synovitis in sternoclavicular joint	1 (1%)
Accessory parotid tissue	1 (1%)
**incidental findings on (low-dose) CT**	
with diagnostic and/or therapeutic consequence	
Lung nodule 4.5 mm, CT evaluation nonspecific, most likely benign	1 (1%)
without diagnostic and/or therapeutic consequence	
Lung nodule <5mm in low-risk patient	3 (2%)
Skeletal cyst dd intraosseous ganglion	1 (1%)
**Other incidental findings upon conduction of [^{18}F]FDG-PET/CT**	
Increased serum glucose, possible diabetes mellitus	1 (1%)
**Total incidental findings**	**41 (31%)**
Total no. of patients with findings with diagnostic and/or therapeutic consequences	22 (17%)
Justified diagnostic and/or therapeutic consequences for disease	3 (2%)
Unbeneficial major consequences***	4 (3%)
Unbeneficial minor consequences****	15 (11%)

FNAC=fine needle aspiration cytology.
*: in one patient, TT was performed because Bethesda VI cytology was obtained from a third nodule during ultrasound and FNAC evaluation of the PET incidentaloma. On histology, this was a mere 3 mm PTC. The [^{18}F]FDG-avid incidentaloma was benign. Therefore, this surgery is considered futile.
**: two malignancies were located ipsilateral to the investigated thyroid nodule.
***: unbeneficial major consequences are defined as substantial therapeutic consequences, i.e., extension of diagnostic surgery to TT for a nodule with final benign histopathology.
****: Unbeneficial minor consequences include minor therapeutic consequences (i.e., nodulectomy in addition to hemithyroidectomy) and overdiagnosis for benign disease that without the [^{18}F]FDG-PET/CT had likely never been detected nor evaluated.

References of the Supplementary Data

1. Herdman M, Gudex C, Lloyd A, Janssen M, Kind P, Parkin D, et al. Development and preliminary testing of the new five-level version of EQ-5D (EQ-5D-5L). Quality of life research : an international journal of quality of life aspects of treatment, care and rehabilitation. 2011;20:1727-36. https://https://doi.org/.org/10.1007/s11136-011-9903-x.
2. Versteegh MM, Vermeulen KM, Evers SMAA, de Wit GA, Prenger R, Stolk EA. Dutch Tariff for the Five-Level Version of EQ-5D. Value in health : the journal of the International Society for Pharmacoeconomics and Outcomes Research. 2016;19:343–52. https://doi.org/10.1016/j.jval.2016.01.003.
3. Stiggelbout AM, Eijkemans MJ, Kiebert GM, Kievit J, Leer JW, De Haes HJ. The 'utility' of the visual analog scale in medical decision making and technology assessment. Is it an alternative to the time trade-off? International journal of technology assessment in health care. 1996;12:291-8. https://doi.org/10.1017/s0266462300009648.
4. EQ-5D-5L User Guide. Rotterdam, the Netherlands: EuroQol Research Foundation; 2019.
5. Bouwmans C, Krol M, Severens H, Koopmanschap M, Brouwer W, Hakkaart-van Roijen L. The iMTA Productivity Cost Questionnaire: A Standardized Instrument for Measuring and Valuing Health-Related Productivity Losses. Value in health : the journal of the International Society for Pharmacoeconomics and Outcomes Research. 2015;18:753-8. https://doi.org/10.1016/j.jval.2015.05.009.
6. iMTA Productivity and Health Research Group. Manual iMTA Medical Cost Questionnaire (iMCQ). Rotterdam: iMTA, Erasmus University Rotterdam. Rotterdam.
7. Dutch Healthcare Authority (NZa). Open data of the Dutch Healthcare Authority (NZa). Utrecht, the Netherlands: Dutch Healthcare Authority (NZa); 2020.
8. Hakkaart-van Roijen L, Tan SS, Bouwmans CAM. Manual for Cost Research.Dutch manual, update appeared in 2004 and 2010. Published by the Dutch Government. Diemen: College voor Zorgverzekeringen; 2010.
9. Dutch Consumer Price index. https://opendata.cbs.nl/statline/#/CBS/nl/. Accessed 14 April 2021.
