# Supplementary material for: [18F]FDG-PET/CT to prevent futile surgery in indeterminate thyroid nodules: a blinded, randomised controlled multicentre trial
Source: Eur J Nucl Med Mol Imaging. 2022 Jan 4;49(6):1970–84. doi: 10.1007/s00259-021-05627-2 (PMC9016050; doi:10.1007/s00259-021-05627-2)
Supplement: Supplementary file 2 — Supplementary file2 (PDF 2827 KB) [file 259_2021_5627_MOESM2_ESM.pdf]

Supplementary Data 2.
Trial Protocol

[^{18}F]FDG-PET/CT to prevent futile surgery in indeterminate thyroid nodules: a blinded, randomised controlled multicentre trial

Elizabeth J. de Koster, Lioe-Fee de Geus-Oei, Adrienne H. Brouwers, Eveline W.C.M. van Dam, Lioe-Ting Dijkhorst-Oei, Adriana C.H. van Engen-van Grunsven, Wilbert B. van den Hout, Tamira K. Klooker, Romana T. Netea-Maier, Marieke Snel, Wim J.G. Oyen, and Dennis Vriens,

for the *EfFECTS trial* study group.

Research Protocol:

Efficacy of [^{18}F]-2-fluoro-2-deoxy-D-glucose Positron Emission Tomography (FDG-PET) in Evaluation of Cytological indeterminate Thyroid nodules prior to Surgery: a multicentre cost-effectiveness study

EfFECTS

RESEARCH PROTOCOL

Version 2.1, February 19, 2021

PROTOCOL TITLE 'Efficacy of [^{18}F]-2-fluoro-2-deoxy-D-glucose Positron Emission Tomography (FDG-PET) in Evaluation of Cytological indeterminate Thyroid nodules prior to Surgery: a multicentre cost-effectiveness study'

Protocol ID	KWF project: KUN 2014-6514<br>CMO ID: NL50166.091.14<br>NCT ID: NCT02208544 (ClinicalTrials.gov)
Short title	EfFECTS
EudraCT number	N/A
Version	2.1
Date	September 23, 2021
Coordinating investigator/project leader	Prof. dr. L.F. de Geus-Oei, MD, PhD<br>Dept. of Radiology and Nuclear Medicine<br>RadboudUMC, Nijmegen<br>Lioe-Fee.deGeus-Oei@radboudumc.nl
Principal investigator(s)	Prof. dr. L.F. de Geus-Oei, MD, PhD<br><br>See Appendix I: Participating Centres and Site Managers (§15.1, p89) and Appendix II: Signed Document of Cooperating Centres (§15.2, p90)
Principal Project Designer	Dr. D. Vriens, MD<br>Dept. of Radiology, section of Nuclear Medicine<br>LUMC, Leiden<br>D.Vriens@lumc.nl
Sponsor	N/a
Subsidising party	Dutch Cancer Society (KWF)
Independent expert(s)	Prof. dr. C.M.L. van Herpen, MD, PhD<br>Dept. of Medical Oncology<br>RadboudUMC, Nijmegen<br>Carla.vanHerpen@radboudumc.nl
Laboratory sites	All analyses of study procedures (FDG-PET/CT, histopathology, genetics) will be performed or reviewed at the RadboudUMC, Nijmegen.<br>Supplementary genetic and tissue analyses will be performed by dept. of pathology (Prof. dr. J. Morreau,

	j.morreau@lumc.nl) at the Leiden University Medical Center (LUMC).
Pharmacy	N/A

PROTOCOL SIGNATURE SHEET

Name	Signature	Date
Head of Department:<br><br>Prof. dr. W.M. Prokop, MD, PhD<br>Dept. of Radiology and Nuclear Medicine<br>RadboudUMC	Research statement signed	08-10-2014
Principal Investigator:<br><br>Prof. dr. L.F. de Geus-Oei, MD, PhD<br>Dept. of Radiology and Nuclear Medicine<br>RadboudUMC		09-04-2015
Coordinating investigator/project leader:<br><br>Prof. dr. L.F. de Geus-Oei, MD, PhD<br>Dept. of Radiology and Nuclear Medicine<br>RadboudUMC		09-04-2015

VERSION HISTORY

Version:	Date:	Remarks:
0.1	October, 2012	First version
0.2	November, 2012	Approval by Project Committee. First administrative changes.
0.3	July, 2014	Issues raised during review of KWF grant processed. Adjustment of protocol to awarded DCS/KWF grant.<br>Version submitted for first review to METC (2014-07-22).
1.0	September, 2014	First operational version, protocol adjusted to remarks of METC and project group.
1.1	October, 2014	Statistical analyses adjusted to single-sided tests. METC approved version.
1.1.1	03-03-2015	LUMC as participating centre added. Added some clarifications with respect to the confirmative US. Reference to the 2015 EANM guidelines. Removed typographical errors.
1.2	09-04-2015	Drawing of whole blood removed from study methods. Slingeland Hospital removed as participating centre. Updated QoL questionnaires. Corrected typographical errors.
1.3	30-12-2015	Participating centres added (HAGA, Rijnstate, OLVG, St. Antonius). Added two new exclusion criteria. Added exception to informed consent procedure.
1.4	05-04-2016	Participating centres added (Isala, Reinier de Graaf).
2.0	20-02-2019	Protocol amendment. Methodology for tissue molecular analysis specified. GDPR.
2.1 (current version)	19-02-2021	Protocol amendment. Recognition of benign histology entities that justify surgery. Changes in methodology for tissue molecular analysis specified.

PROJECT COMMITTEE

Department:	Member:
Nuclear Medicine<br>RadboudUMC	Prof. dr. L.F. de Geus-Oei, MD, PhD (Project Leader)<br>Lioe-Fee.deGeus-Oei@radboudumc.nl<br>Nuclear Medicine physician<br><br>Prof. dr. W.J.G. Oyen, MD, PhD (Project Co-chairman)<br>WOyen@rijnstate.nl<br>Nuclear Medicine physician<br><br>Mr. J.J.C.W. Bosch (Financial Portfolio Manager)<br>Jeroen.Bosch@radboudumc.nl<br>Department Manager<br><br>D. Vriens, MD, PhD (Principal Project Designer)<br>D.Vriens@lumc.nl<br>Nuclear Medicine Physician<br><br>Drs. L. de Koster, MD (Project Executer)<br>Lisanne.deKoster@radboudumc.nl<br>PhD Student
Endocrinology<br>RadboudUMC	Prof. dr. J.W.A. Smit, MD, PhD (Member)<br>Jan.Smit@radboudumc.nl<br>Physician Internal Medicine / Endocrinology<br><br>Dr. R.T. Netea-Maier, MD, PhD (Member)<br>Romana.Netea-Maier@radboudumc.nl<br>Physician Internal Medicine / Endocrinology
Surgical Oncology<br>RadboudUMC	Prof. dr. J.H.W. de Wilt, MD, PhD (Member)<br>Hans.deWilt@radboudumc.nl<br>Oncological/endocrinological surgeon
Health Evidence<br>RadboudUMC / LUMC	Dr. W.B. van den Hout, PhD (Member)<br>W.B.van_den_hout@lumc.nl<br>Health Technology Economist<br><br>Dr. E.M.M. Adang, MD, PhD (Member)<br>Eddy.Adang@radboudumc.nl<br>Epidemiologists, Biostatician and Health Technology Economist
Pathology<br>RadboudUMC	A.C.H. van Engen-van Grunsven, MD (Member)<br>Ilse.vanEngen-vanGrunsven@radboudumc.nl<br>Pathologist<br><br>Dr. B. Kusters, MD, PhD (Member)<br>Benno.Kusters@radboudumc.nl<br>Pathologist
Tissue Genetics<br>LUMC	Prof. dr. J. Morreau, MD, PhD (Member)<br>J.Morreau@lumc.nl<br>Pathologist

Safety Board (§9.5, p62)	Dr. A.R.T. Donders, PhD<br>Rogier.Donders@radboudumc.nl<br>Biostatician<br><br>Dr. J.J. Bonenkamp, PhD<br>Han.Bonenkamp@radboudumc.nl<br>Surgeon<br><br>Prof. dr. J.J. Fütterer, PhD<br>Jurgen.Futterer@radboudumc.nl<br>Interventional Radiologist

TABLE OF CONTENTS

PROTOCOL SIGNATURE SHEET 4
VERSION HISTORY 5
PROJECT COMMITTEE 6
TABLE OF CONTENTS 8
LIST OF ABBREVIATIONS AND RELEVANT DEFINITIONS 12
LIST OF FIGURES AND TABLES 15
Figures 15
Tables 16
SUMMARY 17
1. INTRODUCTION AND RATIONALE 18
1.1 Introduction and problem description 18
1.2 FDG-PET/CT 18
1.3 Role of FDG-PET/CT in FNAC-indeterminate thyroid nodules 19
1.4 Anticipated cost-effectiveness 20
1.4.1 Estimated (direct) Costs 21
1.4.2 Estimated Utility 21
1.4.3 Cost-Effectiveness and sensitivity analysis 22
1.5 Concerns and shortcomings 23
1.6 Underlying molecular biology and phenotypic observations 23
1.7 Alternative diagnostic tests 24
1.8 National relevance 26
1.9 Relevance for practice, implementability & duplications 26
1.10 Laboratory Infrastructure and available techniques 27
1.10.1 FDG-PET/CT 27
1.10.2 Cyto- and histopathology 27
1.10.3 Genetic tissue analysis 27
1.11 Concluding remarks 28
2. OBJECTIVES 29
2.1 Primary (Main) Objective & Hypothesis 29
2.2 Secondary Objectives 30
2.2.1 Secondary Objective 1: Treatment Outcome 30
2.2.2 Secondary Objective 2: Reported Health Related Quality of Life 30
2.2.3 Secondary Objective 3: Costs and Cost-Effectiveness 31
2.2.4 Secondary Objective 4: Cyto-, Histopathological and Tumorgenetic markers 31
3. STUDY DESIGN 32

4. STUDY POPULATION 36
4.1 Population (base) 36
4.2 Inclusion criteria 36
4.3 Exclusion criteria 37
4.4 Sample size calculation 39
4.5 Feasibility 40
5. TREATMENT OF SUBJECTS 41
5.1 Investigational product/treatment 42
5.2 Use of co-intervention 42
5.3 Escape medication 42
6. INVESTIGATIONAL PRODUCT 43
6.1 Name and description of investigational product(s) 43
6.2 Summary of findings from non-clinical studies 43
6.3 Summary of findings from clinical studies 43
6.4 Summary of known and potential risks and benefits 43
6.5 Description and justification of route of administration and dosage 43
6.6 Dosages, dosage modifications and method of administration 43
6.7 Preparation and labelling of Investigational Medicinal Product 43
6.8 Drug accountability 43
7. NON-INVESTIGATIONAL PRODUCT 44
7.1 Name and description of non-investigational product(s) 44
7.2 Summary of findings from non-clinical studies 44
7.3 Summary of findings from clinical studies 44
7.4 Summary of known and potential risks and benefits 44
7.5 Description and justification of route of administration and dosage 44
7.6 Dosages, dosage modifications and method of administration 44
7.7 Preparation and labelling of Non Investigational Medicinal Product 44
7.8 Drug accountability 44
8. METHODS 45
8.1 Study parameters/endpoints 45
8.1.1 Main study parameter/endpoint 45
8.1.2 Secondary study parameters/endpoints 45
8.1.3 Other study parameters 46
8.2 Pre-inclusion study procedures 46
8.2.1 Central Cytology Review 46
8.2.2 Randomisation, stratification, blinding and treatment allocation 47
8.3 Study procedures 48

8.3.1 FDG-PET/CT of head and neck ... 48
8.3.2 Confirmatory Neck US ... 52
8.3.3 Questionnaires ... 52
8.3.4 Cytology and histopathology analysis ... 53
8.3.5 Tissue genetic analysis ... 55
8.4 Withdrawal of individual subjects ... 56
8.4.1 Specific criteria for withdrawal (at the end of the study) ... 56
8.5 Replacement of individual subjects after withdrawal ... 57
8.6 Follow-up of subjects withdrawn from treatment ... 58
8.7 Premature termination of the study (interim-analysis) ... 58
8.8 Mid- to long-term follow-up ... 58
9. SAFETY REPORTING ... 60
9.1 Section 10 WMO event ... 60
9.2 AEs, SAEs and SUSARs ... 60
9.2.1 Adverse events (AEs) ... 60
9.2.2 Serious adverse events (SAEs) ... 60
9.2.3 Suspected unexpected serious adverse reactions (SUSARs) ... 61
9.3 Annual safety report ... 61
9.4 Follow-up of adverse events ... 61
9.5 Data Safety Monitoring Board (DSMB) / Safety Committee ... 62
10. STATISTICAL ANALYSIS ... 64
10.1 General remarks ... 64
10.2 Descriptive statistics ... 65
10.3 Univariate analysis ... 65
10.4 Multivariate analysis ... 68
10.5 Interim analysis ... 68
10.6 Cost-Effectiveness Analysis (CEA) ... 69
11. ETHICAL CONSIDERATIONS ... 71
11.1 Regulation statement ... 71
11.2 Recruitment and consent ... 71
11.3 Objection by minors or incapacitated subjects ... 71
11.4 Benefits and risks assessment, group relatedness ... 72
11.5 Compensation for injury ... 73
11.6 Incentives ... 73
12. ADMINISTRATIVE ASPECTS, MONITORING AND PUBLICATION ... 74
12.1 Handling and storage of data and documents ... 74
12.2 Monitoring and Quality Assurance ... 75

12.3 Amendments ... 75
12.4 Report of start of inclusion ... 76
12.5 Annual progress report ... 76
12.6 Interim Evaluation Report ... 76
12.7 End of study report ... 76
12.8 Public disclosure and publication policy ... 76
13. STRUCTURED RISK ANALYSIS ... 78
13.1 Potential issues of concern ... 78
13.1.1 FDG-PET/CT ... 78
13.1.2 Possible delay of treatment for DTC ... 80
13.2 Synthesis ... 83
14. REFERENCES ... 85
15. APPENDICES ... 89
15.1 Appendix I: Participating Centres and Site Managers ... 89
15.2 Appendix II: Signed Document of Cooperating Centres ... 90
15.3 Appendix III: The Bethesda System for Reporting Thyroid Cytopathology ... 91
15.4 Appendix IV: Performance of FDG-PET/CT in indeterminate thyroid nodules ... 92
15.5 Appendix V: American Joint Committee on Cancer (AJCC) TNM classification of differentiated thyroid cancer, 7th edition ... 94
15.5.1 Introduction ... 94
15.5.2 Primary Tumour (T) ... 94
15.5.3 Regional Lymphnodes (N) ... 95
15.5.4 Distant Metastasis ... 96
15.5.5 Anatomic Stage / Prognostic Groups ... 96
15.6 Appendix VI: Radiation Ethics Form ... 97
15.7 Appendix VII: Data Monitoring Plan ... 98
15.8 Appendix VIII: Questionnaires ... 99
15.9 Appendix IX: Patient information Letter, declaration of insurance, informed consent and travelling expenses declaration form (in Dutch) ... 100

LIST OF ABBREVIATIONS AND RELEVANT DEFINITIONS

Abbrev.	Definition:
(S)AE	(Serious) Adverse Event
^{123}I	Iodine-123, a photon emitter with half-life of 13h
^{124}I	Iodine-124, a positron emitter with half-life 4.2d
^{131}I	Iodine-131, a beta (and gamma) emitter with half-life of 8.0d
^{131}I-RIA	Radio-Iodine thyroid Ablation using Iodine-131
^{99m}Tc	Technetium-99m, a photon emitter with half-life 6.0h
$^{99m}TcO_4^-$	Technetium-99m pertechnetate
ABR	ABR form, General Assessment and Registration form, is the application form that is required for submission to the accredited Ethics Committee (In Dutch, ABR = Algemene Beoordeling en Registratie)
Acc	Accuracy (Bayesian test characteristic)
AE	Adverse Event
AR	Adverse Reaction
CA	Competent Authority
CA-IX	Carbonic Anhydrasis 9: hypoxia marker
Caspase-3	Caspase-3: apoptosis marker
CCMO	Central Committee on Research Involving Human Subjects; in Dutch: Centrale Commissie Mensgebonden Onderzoek
CD3	Cluster of Differentiation 3: Inflammation (T-lymphocyte) cell marker
CD31	Cluster of Differentiation 31: Endothelial marker, marker for vascularity
CD34	Cluster of Differentiation 34: Endothelial marker, marker for vascularity
CEA	Cost-Effectiveness Analysis
CV	Curriculum Vitae
DBC	Diagnosis Treatment Combination (in Dutch: Diagnose Behandel Combinatie)
DFS	Disease-Free Survival
DOT	DBCs aiming at transparency (in Dutch: DBC Op weg naar Transparantie)
DSMB	Data Safety Monitoring Board
DT&PL	Distress Thermometer and Problem Lists
DTC	Differentiated Thyroid Carcinoma
EORTC	European Organisation for Research and Treatment of Cancer
EQ-5D-5L	EuroQol 5 dimensions 5 levels questionnaire
EU	European Union
EudraCT	European drug regulatory affairs Clinical Trials

FDG	[^{18}F]-2-Fluoro-2-deoxy-D-glucose
FN	False Negatives (=test negative in individual with disease)
FNAC	Fine Needle Aspiration Cytology
FP	False Positives (=test positive in individual without disease)
FTC	Follicular Thyroid Carcinoma
Gal-3	Galectin-3: differentiation marker.
GCP	Good Clinical Practice
GDPR	General Data Protection Regulation (in Dutch: Algemene Verordening Gegevensbescherming (AVG))
GLUT	Membrane-bound Sodium-independent Glucose Transporters
HIF-1α	Hypoxia Inducible Factor – 1 alpha: hypoxia marker
HK	Hexokinase
HRQoL	Health Related Quality of Life
IB	Investigator's Brochure
IC	Informed Consent
ICER	Incremental cost-effectiveness ratio
iMCQ	Medical Consumption Questionnaire
IMP	Investigational Medicinal Product
IMPD	Investigational Medicinal Product Dossier
iNMB	Incremental Net Monetary Benefit
iPCQ	Productivity Cost Questionnaire
Ki67	Marker of mitosis (=MIB)
LR-	Negative Likelihood Ratio (Bayesian test characteristic)
LR+	Positive Likelihood Ratio (Bayesian test characteristic)
MC	Medical Centre
METC	Medical research ethics committee (METC); in Dutch: medisch ethische toetsing commissie (METC)
MIB	Marker of mitosis (=Ki67)
mRNA	Messenger Ribonucleic Acid
N/A	Not Applicable
NaI	Sodium Iodine transporter
NNT	Number Needed to Treat
NPV	Negative Predictive Value (Bayesian test characteristic)
OR	Odds Ratio (Bayesian test characteristic)
OS	Overall Survival
PALGA	the nationwide network and registry of histo- and cytopathology in The Netherlands

PET +ve	FDG-PET/CT positive
PET -ve	FDG-PET/CT negative
PET/CT	Positron Emission Tomography / Computed Tomography
PFS	Progression-Free Survival
PI	Principal Investigator
PPV	Positive Predictive Value (Bayesian test characteristic)
Prev	Prevalence
PTC	Papillary Thyroid Carcinoma
RCT	Randomised Controlled Trial
Se	Sensitivity (Bayesian test characteristic)
SF-36	Short Form Health Questionnaire
SOP	Standard Operating Procedure
Sp	Specificity (Bayesian test characteristic)
SPC	Summary of Product Characteristics (in Dutch: officiële productinfomatie IB1-tekst)
Sponsor	The sponsor is the party that commissions the organisation or performance of the research, for example a pharmaceutical company, academic hospital, scientific organisation or investigator. A party that provides funding for a study but does not commission it is not regarded as the sponsor, but referred to as a subsidising party.
STN	Solitary Thyroid Nodule
SUSAR	Suspected Unexpected Serious Adverse Reaction
SUV	Standardised Uptake Value. Parameter expressing the amount of FDG-uptake with respect to patient size (body weight, body surface area or lean body mass) and administered activity. A SUV of 2 implies concentration of FDG twice the amount as can be expected from even distribution throughout the body.
Tg	Thyroglobulin: differentiation marker for thyroid cells
TLG	Total Lesion Glycolysis: area under the volume-SUV histogram
TN	True Negatives (=test negative in individual without disease)
TP	True Positives (=test positive in individual with disease)
US	UltraSonography
VAS	Visual-Analogue Scale
WGBO	Law on Medical Treatment Agreement (in Dutch: Wet op de Geneeskundige BehandelingsOvereenkomst)
WMO	Medical Research Involving Human Subjects Act (in Dutch: Wet Medisch-wetenschappelijk Onderzoek met Mensen

LIST OF FIGURES AND TABLES

Figures

Figure 1: Flow-chart of the trial 34
Figure 2: Relation between study size (N), study-arm size sizes (n_1 and n_2) and effect size. Empirical relation: N=16.33935*ES$^{-1.86986}$, R^2=.999443). Expected ES=73.1%-40.3%=32.8% 40
Figure 3: Position of the normal thyroid. 49
Figure 4: Topograms showing the major landmarks: the meatus acusticus externus (*MAE*), the fossa jugularis sternalis (*FJS*) and the lower margin of the arcus aortae (*LAA*). 50
Figure 5: Example of a FDG-PET/CT positive thyroid nodule in the isthmus, which proved to be a 10 mm differentiated papillary thyroid carcinoma (pT1a cN0M0) 51
Figure 6: Anatomy and subdivision of the normal thyroid gland 54
Figure 7: Cumulative Binomial Distribution for a sample size of 22 (interim-analysis) or 44 (end-of-study evaluation) for an individual patient probability of a false-negative FDG-PET/CT of 0.05. In case that more than 4/22 patients (18.2%) at time of the interim analysis or 6/44 (13.6%) patients at time of the end-of-study evaluation have a false-positive FDG-PET/CT (in the control study arm), the false-negative probability is larger than the maximum accepted value of 0.05. 57
Figure 8: Results from meta-analysis on test characteristics of FDG-PET/CT in indeterminate thyroid noudles [4]. CI: confidence interval; FE: fixed-effects; FN: false negative; FNAB: fine-needle aspiration biopsy; FP: false positive; LR-: negative likelihood ratio; LR+: positive likelihood ratio; NPV: negative predictive value; PPV: positive predictive value; RE: random-effects; TN: true negative; TP: true positive. 92
Figure 9: Test characteristics of FDG-PET for detection of malignancy in FNAC-indeterminate thyroid nodules based on definite size (histopathology). Cut-offs, based on the TNM-classification (7th revision) are displayed. Unpublished data from [4]. Acc: accuracy; NPV: negative predictive value; PET-: fraction of PET-negative nodules; PPV: positive predictive value; Se: sensitivity; Sp: Specificiy. For lesion-diameter ≥15mm, sensitivity is 1005 (92-100%), specificity is 47% (38-56%). 93
Figure 10: Lymphnode compartments separated into levels and sublevels. 95

Tables

Table 1: Participating hospitals and their estimated number of eligible patients.................... 33
Table 2: Summary of Protocol Investigations/Procedures. FNAC: fine-needle aspiration cytology; fT4: free thyroxine; FDG-PET/CT: Positron Emission Tomography using FDG of head, neck and upper thorax; RIA: radio-iodine ablation using ^{131}I; TSH: thyroid stimulating hormone; US: ultrasonography; [1]only on indication; [2]only in FDG-PET/CT negative patients in arm A; [3]only in FDG-PET/CT positive patients in arm A, [4]Additional tests such as gene-expression classification, mutational marker panel, US elastography are allowed in the mid- to longterm follow-up at the instigation of the treating physician (but are not reimbursed by the study funds). [*]Part of study protocol, reimbursed by study funds.. 35
Table 3: Estimated eligible patients and finally included number of patients per participating centre(Appendix II: Signed Document of Cooperating Centres, §15.2, p88)......................... 41
Table 4: Stratification during study including expected fractions (based on [4] and [57]). AUS/FLUS: Atypia of Undetermined Significance or Follicular Lesion of Undetermined Significance; Rep: repeatedly; SFN: (Suspicious for) Follicular Neoplasm........................... 48
Table 5: Handling of descriptive statistics. *parameters are estimated on the logtransformed variable and back-transformed. 95%-CI: 95%-confidence interval using normal distribution (mean±1.9600 x standard deviation). IQR: interquartile range (p_{25} and p_{50})......................... 65
Table 6: General Requirements for selection of Statistical tests used for univariate analysis (comparison) 66
Table 7: Parameters describing association between variables. 66
Table 8: Comparison between parameters describe association 67
Table 9: Definition of true positive (TP), true negative (TN), false-positive (FP) and false-negative (FN). 67
Table 10: Models for assessment of independency in multivariate modelling. 68
Table 11: Risk classification according to the ICRP in relation with social benefit. *with the exeption of experimental-therapeutic applications, the dose needs to be below the threshold for deterministic effects. 79
Table 12: Risk classification in relation to the probability and severity of potential injury. 84
Table 13: The Bethesda System for Reporting Thyroid Cytopathology. The orange shaded categories are included in this study. 91
Table 14: T(NM) stage of differentiated thyroid carcinoma, 7th edition. 94
Table 15: (T)N(M) stage of differentiated thyroid carcinoma, 7th edition. 95
Table 16: (TN)M stage of differentiated thyroid carcinoma, 7th edition. 96
Table 17: Anatomic staging of differentiated thyroid carcinoma 96

SUMMARY

Rationale: Only about ¼ of patients with thyroid nodules with indeterminate cytology are proven to suffer from a malignancy at diagnostic hemithyroidectomy. Therefore ~¾ is operated upon unbeneficially. Recent studies using FDG-PET/CT have suggested that it can decrease the fraction of unbeneficial procedures from ~73% to ~40%. Thereby the direct costs per patient, the number of hospitalization and average sick leave days might decrease and the experienced HRQoL might increase. A study will be undertaken to show the additional value of FDG-PET/CT after indeterminate cytology with respect to unbeneficial procedures, costs and utilities. **Main objective**: To determine the impact of FDG-PET/CT on decreasing the fraction of patients with cytologically indeterminate thyroid nodules undergoing unbeneficial patient management. **Study design:** A prospective, multicentre, randomised, stratified controlled blinded trial with an experimental study-arm (FDG-PET/CT-driven) and a control study-arm (diagnostic hemithyroidectomy, independent of FDG-PET/CT-result). **Study population:** Adult patients with a cytologically indeterminate thyroid nodule, without exclusion criteria, in 15 (university and regional) hospitals distributed over the Netherlands. **Intervention**: One single FDG-PET/low-dose non-contrast enhanced CT of the head and neck is performed in all patients. Patient management depends on allocation and results of this FDG-PET/CT. **Main study parameters/endpoints:** The number of unbeneficial interventions, i.e. surgery for benign disease or watchful-waiting for malignancy. For secondary objectives: complication rate, consequences of incidental PET-findings, number of hospitalisation and sick leave days, volumes of healthcare consumed, experienced HRQoL, genetic, cytological and (immuno)histopathological features of the nodules. **Sample size calculation/data analysis:** Based on above-mentioned estimated reduction in unbeneficial interventions from ~73% to ~40%, at least 90 patients with nodules>10 mm need to be analysed (2:1 allocation, $\alpha=0.05$, power=0.90, one-tailed Fisher's exact test). After correction for nodule size and data-attrition, 132 patients need to be included in total. Intention-to-treat analysis will be performed. Incremental Net Monetary Benefit based on the total direct costs per patient and the gain in HRQoL-adjusted survival years are computed. Cytological, histological and genetic parameters and their correlation to FDG-avidity will be described. **Nature and extent of the burden and risks associated with participation, benefit and group relatedness:** All patients undergo one FDG-PET/CT scan of head/neck (effective dose: <3.5 mSv) and are asked to fill in 6 questionnaires at 4 time points. FDG-PET/CT negative patients in the experimental arm will undergo a single confirmatory US (±FNAC). An interim/posterior analysis of the control subjects is performed to ensure oncological safety. In case of an unexpected high false-negative ratio in this control arm, all patients will be advised to undergo surgery.

1. INTRODUCTION AND RATIONALE

1.1 Introduction and problem description

Differentiated thyroid carcinoma (DTC) is rare (incidence: ~13-31/million patient years [1]). Although the prognosis is favourable, its morbidity is high. As most patient present with a palpable thyroid nodule (TN), with a lifetime prevalence of 3-8%, and the chance of cancer in any such a nodule is less than 5%, screening for cancer is warranted once a nodule is established. The prevalence of TNs increases with age. Nowadays, due to the increasing use of ultrasound (US) and other imaging techniques more and more asymptomatic TNs are discovered, the majority of which have no clinical relevance. As initial instrument, (US guided) fine-needle aspiration cytology (FNAC) is recommended. Aspirates are classified in six diagnostic categories according to the Bethesda System for Reporting Thyroid Cytopathology (Appendix III: The Bethesda System for Reporting Thyroid Cytopathology, §15.3, p91) [2, 3]. In about 75% of patients, this will lead to a definite diagnosis and treatment: either benign (Bethesda category II), suspicious (V) or definite malignant (VI). However in the remaining cases, repetitive FNAC cannot determine whether the lesion is benign or malignant, due to cellular atypia (III), follicular neoplasia (V) or repetitive nondiagnostic or unsatisfactory specimens (I). According to current practice, in all these patients diagnostic surgery is performed to reach a final histological diagnosis [4-6]. In about 69-88% these patients, the nodule is benign, and surgery was therefore futile. Especially in case the nodule was asymptomatic, the surgery was unbeneficial. Apart from symptoms and cosmetic complaints of the neck scar, about up to one third of these patients need lifelong daily thyroid hormone suppletion. Furthermore, although rare, surgical complications may be severe (haemorrhage, infection, permanent hoarseness) [7-9]. Finally, surgery, hospitalisation and sick-leave are costly. In most malignant thyroid nodules (~20-25% of operated patients), secondary surgery with adjuvant treatment including radio-active I-131 thyroid remnant ablation, has to be performed. Only in case of subcentimetre, indolent, unifocal papillary microcarcinoma (UPM), additional treatment is unnecessary.

1.2 FDG-PET/CT

The proposed study capitalizes on one of the assets of positron emission tomography using the radiofluorinated glucose analogue FDG (FDG-PET/CT): this technique cannot only visualize but can also quantify FDG-uptake and thus is able to distinguish metabolically (glycolytic) highly active tissue from less active tissue. It offers an opportunity for non-invasive, in vivo tissue characterization and could play a role in further characterisation of lesions. As many factors can influence the extent of FDG-uptake, the underlying mechanics of FDG-

accumulation in tissue, are still a matter of debate. This is addressed in this proposal as a secondary objective. A variety of mechanisms have been proposed for accelerated glucose use in tissue: tumour cells predominantly rely on anaerobic (lactate producing) glycolysis instead of mitochondrial oxidative phosphorylation, even in the presence of oxygen (Warburg effect). To support the necessary high rate of glycolysis, cells need an increased transport of glucose in the cells, facilitated by upregulation of sodium-dependent membrane-bound glucose transporters (GLUTs). Glucose transport activity can be regulated by alterations in the expression of GLUT and by post-translational mechanisms, including transporter translocation to plasma membranes. Increased concentration of the first enzyme in the glycolytic pathway (hexokinase, HK) with decreased rates of the gluconeogenesis enzyme glucose-6-phosphatase are considered to accelerate glucose phosphorylation and thus enhanced FDG-trapping. FDG-uptake has been correlated with tumour doubling time and proliferation rates, and in thyroid carcinoma FDG-uptake is correlated with tumour aggressiveness and dedifferentiation. Yet, it is currently unknown exactly which underlying tissue gene alterations are related to FDG-uptake in benign or malignant thyroid lesions. Finally, the presence of (FDG-avid) inflammatory cells might be confounding, since these may have a major impact on FDG-uptake. At present it is still poorly elucidated which of these and other factors contribute to FDG-uptake in thyroid lesions.

The limited spatial resolution of a PET-scanner, may lead to underestimation of the true FDG-uptake in smaller lesions, generally smaller than 2-3 times the resolution which is about ~3 mm full width at half maximum (FWHM) in state-of-art scanners and ~5 mm FWHM in previous-generation scanners. As most palpable thyroid nodules are larger than 10 mm and both the risk of malignancy in these nodules is much smaller and the clinical relevance of UPMs are limited, we will focus primarily on lesions larger than 10 mm. As previously mentioned, thyroid US is able to detect even smaller nodules.

FDG-PET/CT is an established and cost-effective procedure for a number of indications. FDG-PET/CT is non-invasive and has no known adverse events related to the administration of FDG. The effective patient dose of a *head and neck* FDG-PET/CT for a healthy 70 kg adult is less than 3.5 mSv, much lower than a diagnostic CT of the head and neck. A PET/CT-scanner is widely available and accessible for all patients in the Netherlands.

1.3 Role of FDG-PET/CT in FNAC-indeterminate thyroid nodules

In 2006, our group published a prospective study on the results of FDG-PET in 44 patients with FNAC-indeterminate thyroid nodules. We described a positive and negative predictive value for malignancy of 32% and 100%, respectively (prevalence: 14%) [10]. As our findings were supported by several other studies [11-15], all with similar negative predictive values but

varying positive predictive values, we performed a systematic review and meta-analysis of the literature [4]. The prevalence of benign disease, histologically determined in all these patients after resection, was 74%. In all these patients, FDG-PET/CT was performed prior to surgery on previous generation PET/CT-scanners. Only 3 out of 83 patients with a negative FDG-PET and 55 out of 142 patients with a positive scan showed a malignancy at diagnostic surgery (negative and positive predictive value: 96% and 39%, respectively. The sensitivity of FDG-PET/CT was strongly related to the size of the TN, due to limited spatial resolution of the PET(/CT) scanners used. Due to the limited specificity of the FDG-PET/CT for malignancy, the positive predictive value was found to be poor (Appendix IV: Performance of FDG-PET/CT in indeterminate thyroid nodules, §15.4, p92). Very recently, these data were confirmed by a prospective series of 46 patents with FNAC (non-oncocytic) follicular lesions (Bethesda category III and IV) larger than 20 mm (DTC prevalence: 28%) with a negative and positive predictive value of 61% and 94%, respectively [16]. The authors state that FDG-PET/CT could reduce the number of diagnostic (hemi)thyroidectomies by 13-25%.
Based on the high sensitivity of FDG-PET/CT to detect malignancy in FNAC-indeterminate thyroid nodules, we postulate that incorporation of this imaging modality in the diagnostic work-up of these nodules could decrease the prevalence of benign nodules at the time of diagnostic hemithyroidectomy from 74% to 39%. Due to the limited specificity of FDG-PET/CT, still 61% of operated patients will be diagnosed with a benign disorder (i.e. patients with false-positive FDG-PET/CT), being 74% prior to the inclusion of FDG-PET. Although this seems a moderate improvement, the number of hemithyroidectomies for FNAC-indeterminate nodules is reduced by 37%. We therefore consider this modality potentially valuable tool in this category of patients.
We have described a false-negative ratio of FDG-PET/CT in ~1.3% of all patients, which is within the range of the a priori risk of DTC in any thyroid nodule (<5%). Sensitivity is highly dependent of nodule size due to the earlier mentioned effect of limited spatial resolution of a PET-scanner (5-8 mm FWHM on PET/CT-scanners from the meta-analysis, 3-4 mm for current time-of-flight PET/CT-scanners). This false-negative ratio could delay treatment for thyroid malignancy, but the consequences are considered to be minimal, due to the relative indolent course of this disease. Furthermore, there is limited impact on survival upon the transition from localised (stage I) to regional (stage II) disease (5-year overall survival for both groups is 100%), all with good (curative) treatment options [5].

1.4 Anticipated cost-effectiveness

We developed an 8-state Markov decision model [17]. Based on literature, reimbursement schedules of diagnosis/treatment combinations and expert panel opinion, we attributed

distributions to the transition probabilities, costs and utility scores (HRQoL). Analysis of the model was performed by probabilistic sensitivity analysis for hypothetical adult patients with FNAC-indeterminate TNs over a duration of 5 years, either with or without full implementation of FDG-PET/CT. Means and confidence intervals of discounted costs and QALYs were determined. Efficiency of FDG-PET/CT was presented by the incremental net monetary benefit (iNMB) in €, based on a willingness to pay of 80,000 € /QALY. We performed one-way sensitivity analysis over a wide range for all parameters as described in international literature. We used the results from our meta-analysis for estimations of the FDG-PET/CT sensitivity and specificity, which is based on previous-generation PET-scanners.

1.4.1 Estimated (direct) Costs

The direct treatment costs are mainly driven by the expenses for surgery and hospitalization. When these costs are adjusted to the current (2013) price level using Customer Price Indexation (CPI), these would be € 4,419, € 6,238, € 6,618 and € 2,479 for hemithyroidectomy, total thyroidectomy, completion thyroidectomy and radioiodine I-131 thyroid remnant ablation, respectively. Additional costs are made for out-patient visits, lowest in case of a benign node or UPM found during hemithyroidectomy (first year: € 1,080, none thereafter) and highest in case permanent complications (first year: € 5,282, year 2-5: € 899/year). In case of FDG-PET/CT is used, additional costs for the scan (€ 1,002) and, in case of a non-avid node, follow-up without surgery (first year: € 488, year 2-5: € 314/year) are included.
Modifying current practice with routine 100% adaptation of FDG-PET/CT resulted in 46% fewer surgeries for benign nodules. Compared to surgery in all patients, the fraction of untreated cancers was 1.3%, similar as reported in literature. Over 5 years, mean discounted cost estimates were € 8,804 (95%-confidence interval: € 8,774- € 8,835) for current practice and € 7,983 (95%-confidence interval: € 7,941- € 8,025) with the routine use of FDG-PET/CT.

1.4.2 Estimated Utility

Utility is expressed in Quality-adjusted Life Years (QALYs), a measure of disease burden, including both the quantity and the quality of the life lived.
As the life expectancy (quantity) in the patients under investigation is close to the normal population, we do not expect to find a different survival ratio between both study arms. As the median patient age at diagnosis (of DTC) is about 49 years, the remaining expected life years in the Netherlands is 30.9 for males and 34.5 for females, including many productivity years.
However, based on published data on a similar diagnostic test, we expect that prevention of unbeneficial surgery might increase the HRQoL (quality) in the patients under investigation,

thereby increasing the QALYs in the experimental arm (4.50 to 4.57 QALY over the first 5 years) [18]. This is in concordance with a recent systematic review in DTC survivors: HRQoL was only diminished shortly after surgery, but returns to preoperative levels when time since surgery increased. The overall HRQoL in DTC survivors is similar or slightly worse compared with the normative population [19].

Using HRQoL estimates in the Markov decision model, we have found that current practice and FDG-PET/CT indeed produced no relevant difference in QALYs; they were 4.516 and 4.552 QALY for both 5-year scenarios, respectively.

1.4.3 Cost-Effectiveness and sensitivity analysis

Implementation of the proposed FDG-PET/CT strategy has the potential to save cost (€ 822) and increase HRQoL (0.036 QALY) compared to the present alternative meaning that FDG-PET/CT strategy was the dominant and consequently the efficient alternative. The iNMB is estimated to be € 3,684 (95%-confidence interval: € 3,278 - € 4,094). Also compared to the, in the USA routinely used gene expression classifier test on cytological material, FDG-PET/CT would save costs (€ 1,358) without detrimental effects on the HRQoL (4.556 vs 4.552 QALYs per 5-years for gene expression classifier and FDG-PET/CT, respectively).

One-way sensitivity analysis for the ranges of transition probabilities tested, showed that in all cases FDG-PET/CT was the dominant alternative. Most influential parameters were fraction of permanent complication due to hemithyroidectomy (range tested: 1-26%), FDG-PET/CT specificity (range tested: 35-70%) and the yearly fraction of patients that are observed after a negative FDG-PET/CT and undergo surgery for a (new) suspicion of malignancy (range tested: 0-5%).

The most influential parameter (under assumptions of independency) was found to be the utility attributed to surveillance after a negative FDG-PET/CT scan. At the minimum evaluated value (0.90), a worse quality of life was found for FDG-PET/CT-driven treatment versus thyroid surgery in all patients (mean incremental utility: -0.10 QALY) leading to a mean iNMB of € -7,418. At a value for the utility attributed to watchful surveillance of 0.953, the mean iNMB equals € 0. For comparison, the utility attributed to the healthstate after uncomplicated hemithyroidectomy was set at 0.99.

Other parameters that proved influential in affecting cost-effectiveness included the utility of surveillance and permanent complications after hemithyroidectomy, the probability of hemithyroidectomy-induced (transient and permanent) complications, the probability of performing hemithyroidectomy as primary method for thyroid surgery and surgical mortality as well as the costs of a hemithyroidectomy procedure. Finally, specificity and sensitivity of FDG-

PET/CT influenced the model outcome, but all these parameters did not lead to a negative iNMB for the ranges tested.

1.5 Concerns and shortcomings

Recently, Deandreis et al. [20] described 55 patients scheduled for surgery of FNAC-indeterminate thyroid nodules. Of the nodules, 61% was benign and 18% definitely malignant. The remaining 21% was of uncertain malignant potential. They described a positive and negative predictive value of 57% and 81% respectively for definite and uncertain malignant TNs. They reported 5 FDG-PET/CT false negative lesions (3 tumours of uncertain malignant potential, 2 papillary carcinomas of 10 and 20mm) and they explain the discordance of their findings compared to other publications, by suggesting that it may be related to the definition of “indeterminate cytology” itself or to the heterogeneity of the pathology itself (small and/or well-differentiated tumours show limited FDG-uptake). There is an exceptionally large fraction of lesions with uncertain malignant potential and it can be debated whether these lesions should have been qualified as benign or malignant. The authors chose the latter, but a negative predictive value of 92% can be computed when these lesions of uncertain malignant potential are considered benign, which is much more concordant with previous reports in literature. Twenty-five of 55 scans (45%) were FDG-PET/CT negative, comparable to our meta-analysis (37%).

False-positivity, or a poor positive predictive value, may also be a concern, as these patients still undergo unbeneficial surgery. The only independent predictive factor for FDG-uptake was cellular atypia (present in both benign and malignant nodules). Current literature mainly focuses on FDG-uptake in known thyroid carcinoma [21-24] or (in vitro) in thyroid cells [25], therefore the limited specificity of FDG-PET/CT for (FNAC-indeterminate) thyroid nodules is still poorly understood. Whether previously described diagnostic tests (e.g. immunohistochemistry, gene expression classification) show complementary or additional value needs to be determined.

To our knowledge no studies have been performed to investigate the effect of a wait-and-see policy in patients with a very low probability of malignancy in cytological indeterminate nodules. The effects on HRQoL and costs are only modelled and not prospectively evaluated.

1.6 Underlying molecular biology and phenotypic observations

In thyroid carcinoma, increased glucose metabolism seems to be restricted to more aggressive and high-grade tumours, whereas tumours with favourable prognosis demonstrate no significant tracer uptake. In formalin-fixed and paraffin-embedded tissue samples of 45 patients with thyroid carcinoma (20 papillary, 20 follicular and 5 anaplastic) it was found that

GLUT-1 expression as closely related to the grade of malignancy in thyroid neoplasms, but GLUT-2-5 were not [21]. Hooft et al. [22] compared the in vivo FDG-uptake with biomarkers in 19 patients with recurrent DTC and found strong association with FDG-uptake and HK-I expression. They could not demonstrate significant correlation between FDG-uptake and expression of GLUT-1, HK-2 and -3, CD68 (macrophage marker), CD31/CD34 (vascular epithelial marker), VEGF-165 (vascular endothelial growth factor, a major angiogenetic factor in cancer), HIF-1α (hypoxia-inducible factor), Tg (thyroglobulin, specific for differentiated thyroid tissue), Cyclin A, Ki67 and p53 (proliferation markers). Another study examined 54 patients with papillary thyroid cancer by FDG-PET/CT and correlated the FDG-uptake level (maximal standardised uptake value, SUV) and found a relation between maximal SUV and expression of GLUT-3 and -4 and tumour size but failed to demonstrate a correlation between maximal SUV and GLUT-1 expression, patient age, sex, extrathyroidal extension and lymphnode metastases [24]. Interestingly (for imaging and treatment), Barollo et al. [26] describe the relation between BRAF pV600E mutation in 50 patients with primary and recurrent papillary thyroid cancer and the uptake of FDG- and [I-131] uptake. They describe that only 6% of patients with a BRAF pV600E-positive recurrence could concentrate I-131, the majority could concentrate FDG. However, papillary lesions are most of the time not indeterminate as confirmed recently [27]. Finally, Deichen et al. [25] performed an in vitro study of the relation between FDG-uptake and TSH levels in benign human thyroid cells and concluded that TSH significantly increased FDG-uptake in vitro in a time- and concentration-dependent manner. In the earlier quoted study by Deandreis et al.[20] on the relation between FDG-avidity and histological and ultrasonographic patterns, they describe only a relation between FDG-avidity and cellular atypia, presence of Hürthle cells, capsular infiltration, differentiation grade and microcalcifications, but not with inflammatory infiltration, necrosis, mitosis, vascular emboli, presence of tumour capsule, echogenicity, US-homogeneity, US-regularity of margins, solid or cystic textural features, hypervascularisation or hypoechoic halo.

1.7 Alternative diagnostic tests

Other diagnostic tests are currently under investigation, and include the analysis of molecular biomarkers on cytology, or conventional or molecular imaging.

A recent publication from our group provided a complete and extensive overview of all additional molecular and imaging diagnostics for indeterminate thyroid nodules in a preoperative clinical setting, including considerations regarding cost-effectiveness, availability, and feasibility of combining techniques [28].

Endorsing the 2015 ATA guidelines, our review demonstrated that various molecular tests have great diagnostic potential through their high diagnostic accuracy. Over the past decade, a number of molecular biomarker panels have taken an important place in the research field of pre-operative diagnostics for cytologically indeterminate thyroid nodules. The most frequently investigated of these tests is a commercialized gene-expression classifier, which showed has 83%-100% sensitivity and 10%-52% specificity [29-32]. This test is currently not available outside the USA and is very expensive (~$3500 per sample). Another 7-gene molecular marker panel showed 18%-69% sensitivity and 86%-99% specificity [33-37]. According to the previously described economic analysis, approaches using either of these two tests are less cost-effective than an FDG-PET/CT-driven approach [17].
In recent years, the 7-gene molecular marker panel has evolved. Now, it uses next-generation sequencing and also includes additional gene mutations, fusions, and translocations, copy number variations and a microRNA gene expression panel. Sensitivity and specificity of this test range between 90%-94% and 82%-93%, respectively, but clinical validation studies of the newest version of the test are still ongoing [38-40]. This test is also currently unavailable outside the USA and very expensive (~$3800 per sample) [28].
Immunocytochemistry to demonstrate protein-expression on cytology is less complicated to perform, less expensive and more widely available [41]. However, its diagnostic performance seems inferior to gene mutation and gene expression analysis and there is no global consensus on the methodology for this technique. Galectin-3, HBME-1 and CK-19 expression are most regularly investigated, but the number of original studies is limited. Presence of any of these markers suggests papillary thyroid carcinoma, although their sensitivities and specificities fall short to reliably exclude or confirm malignancy [28].

As a conventional imaging test, US elastosonography shows potential in the pre-operative diagnostic work-up of indeterminate thyroid nodules. Highly dependent on the assessment method (i.e. qualitative or semi-quantitative assessment, chosen cut-off values), it demonstrated 47%-97% sensitivity and up to 100% specificity [42-45]. US elastosonography is inexpensive and widely available. Nonetheless, varying methodology between the two dozen available studies, limit appropriate validation of its diagnostic accuracy until today [28].

Finally, ^{99m}Tc-MIBI scintigraphy is another available molecular imaging technique, which is mostly popular in Southern Europe. ^{99m}Tc-MIBI scintigraphy has lower costs (~€119-€500 per scan) and higher availability than FDG-PET/CT. Its diagnostic accuracy is also presumed lower: sensitivity and specificity range between 56-79% and 52%-96%, respectively [28, 43, 46, 47]. The number of clinical validation studies is limited. One of the limitations of this

technique is that thyroid nodules with Hürthle cell cytology often have a false-positive test result [28].

Original studies investigating a combination of techniques, especially a combination of molecular biomarkers and molecular imaging, are rare [28, 43, 46]. Yet, it is highly likely that the highest diagnostic accuracy can be achieved by combining multiple pre-operative tests in a step-wise manner. Investigating this, including the consequences for cost-effectiveness, is one of the secondary objectives of the current study.

1.8 National relevance

About 4,123 FNAC examinations of the thyroid gland were performed in the Netherlands in 2003 and numbers are increasing with ~4.5% per year [48]. After selection for indeterminate cytology (11-24%) and correction for duplicates or indications other than thyroid nodules, we expect that approximately 1,000 patients per year nationwide will fulfil the eligibility criteria of our study. This corresponds with estimates from recent communication with PALGA (the nationwide network and registry of histo- and cytopathology in The Netherlands), July 2012.
Based on these numbers, full implementation of the proposed strategy could prevent unbeneficial surgery for benign disease in ~342 patients per year at the expense of a delay of final treatment for DTC in ~13 patients annually. This would lead to a nationwide gain in about 36 QALYs per year and a maximal direct cost-saving of ~ € 807,000. These numbers could further increase as the reimbursement rate of FDG-PET/CT declines and one accounts for the economic benefit of immaterial costs, decrease in loss of productivity and other indirect patient-oriented costs as well.

1.9 Relevance for practice, implementability & duplications

Although the diagnostic test-characteristics of FDG-PET/CT for this indication are well-investigated, currently no prospective and randomised studies have been performed, investigating the influence of implementation on efficacy, cost-effectiveness and national budget. Its introduction in clinical practice has not taken place, most probably due to lack of prospective evidence.
By prospectively demonstrating efficacy, cost-effectiveness and implementability, we aim at formalizing the application of FDG-PET/CT in national and international guidelines, such as those developed by the Comprehensive Cancer Centre Netherlands (IKNL) [5] and the American Thyroid Association Guidelines on Thyroid Nodules [6].

The proposed strategy will not lead to diversions of budgets from intramural to extramural care. However, minor shifts in referral patterns between hospitals may occur as not all hospitals provide FDG-PET/CT.
At present, according to multiple (inter)national trial-databases no similar studies have been or are currently being performed (ClinicalTrials.gov, Dutch Trial Registry, metaRegister of Controlled Trials, WHO ICTRP Search Portal and CEA Registry, accessed on 02/22/2019).

1.10 Laboratory Infrastructure and available techniques

1.10.1 FDG-PET/CT

Central review of FDG-PET/CT scans is performed at the Radboudumc. The department of radiology and nuclear medicine of the Radboudumc has ample experience with oncological nuclear medicine and thyroid metabolism. Moreover, the staff participates in and leads multiple (inter)national multicentre trials and the Radboudumc is a secondary referral centre for the region. Therefore, the physicians are used to assess FDG-PET/CTs from different scanners and to take part in multiple trials.

1.10.2 Cyto- and histopathology

Central review of all cytopathological and histopathological tissue is performed at the Radboudumc. For cases requiring specific additional expertise (e.g. noninvasive follicular thyroid neoplasm with papillary-like nuclear features), the specific expertise of the dept. of pathology of the LUMC may be sought. Both departments of pathology have a long tradition as a referral supraregional centre for thyroid pathology. Currently many studies are being undertaken involving both the departments of nuclear medicine and pathology.
The departments of radiology and nuclear medicine and the department of pathology have collaborated to develop and implement the current trial in the past years.

1.10.3 Genetic tissue analysis

To be able to perform tissue molecular analysis using the desired state-of-the-art techniques, the molecular analysis on cytology will be performed at the department of pathology at the Leiden University Medical Centre (LUMC), using next generation sequencing (also see §8.3.5). If appropriate (see §8.3.5), any tissue molecular analysis on histology will be performed at the LUMC. These techniques are not available at the Radboudumc.

1.11 Concluding remarks

In this series of studies we compiled current knowledge on efficacy, cost- effectiveness and the magnitude of problem burden. FDG-PET/CT in FNAC-indeterminate thyroid nodules holds promise to prevent futile surgery, to be cost-effective and to improve HRQoL. A better understanding of the biological mechanisms and biomarkers involved in glucose metabolism and tumorigenesis can help to explain the metabolism and malignant potential of thyroid nodules and its reflection on FDG-PET/CT as described earlier. Moreover FDG-PET/CT can help to accurately guide management decision in a non-invasive, more efficient, cost-effective way, improving the quality of life of patients and declining the number of unbeneficial (and potentially harmful) surgical diagnostic procedures.

2. OBJECTIVES

2.1 Primary (Main) Objective & Hypothesis

Objective:

Can the implementation of FDG-PET/CT of the head/neck region in the work-up of adult patients with thyroid nodules with indeterminate FNAC (Bethesda category: III and IV) decrease the number of patients undergoing unbeneficial treatment, i.e. surgery for benign disease or watchful-waiting for malignant disease, focussing on lesions >10mm?

Remarks:

We will focus on the lesions larger than 10mm, as T1a lesions ("microcarcinomas") cause technical challenges due to the finite resolution of a PET-scanner and previous publications, forming the basis of our numerical hypothesis, have only limitedly included these T1a lesions. We will ***not*** *exclude these patients however, but include them to be able to describe FDG-PET/CT performance in all patients (see SO1a).*

Some studies include repetitive non-diagnostic FNACs (Bethesda category I) but these are not defined as 'indeterminate' and are a heterogeneous group and are therefore not included.

Hypothesis:

Full implementation of FDG-PET/CT can decrease the number of patients undergoing unbeneficial treatment to ~40%. Currently ~73% of patients with a cytologically indeterminate thyroid nodule is unbeneficially operated for benign nodes.

2.2 Secondary Objectives

2.2.1 Secondary Objective 1: Treatment Outcome

- SO1a: To determine the difference in unbeneficial treatment between FDG-PET/CT driven and routine surgery in this population including thyroid lesions of all sizes (i.e. both ≤10 mm and >10mm).
- SO1b: To determine the effect of incorporation of FDG-PET/CT on the complication-ratio.
- SO1c: To determine the false-negative rate of FDG-PET/CT in this population (i.e. in how many patients with malignancy will watchful-waiting be performed) based on both 12-month and (retrospective) 5-year follow-up.
- SO1d: To determine the influence of lesion size, pathological classification and patient characteristics on the diagnostic accuracy (subgroup analysis) of FDG-PET/CT.
- SO1e: To determine whether incorporation of FDG-PET/CT of the head and neck leads to overdiagnosis in non-thyroidal incidental findings (whole-group analysis).
- SO1f: To determine the short-term overall and disease free survival in both study arms.
- SO1g: To determine which factors hamper implementation of this modality for this indication (structured interviews).
- SO1h: To determine the fraction of patients that cannot be reassured by a negative PET-scan (experimental arm only) despite careful selection of patients (implementability).

2.2.2 Secondary Objective 2: Reported Health Related Quality of Life

- SO2a: To determine the impact on the experienced HRQoL between the group with and without FDG-PET/CT according to 4 different questionnaires at 4 timepoints during the first 12 months after FDG-PET/CT.
- SO2b: To determine whether patients in the experimental arm with negative PET-findings have a different HRQoL than those who receive surgery independent of the FDG-PET/CT results.

2.2.3 Secondary Objective 3: Costs and Cost-Effectiveness

- SO3a: To determine the effect of incorporation of FDG-PET/CT on the mean direct costs (=volume of care multiplied by activity-based costs) per patient during the first 12 months after FDG-PET/CT.
- SO3b: To determine the effect of incorporation of FDG-PET/CT on the average length of hospital stay for treatment of (complications of) thyroid lesions?
- SO3c: To determine the total number of sick leave days for the first three months in the patients (recall, supported by 2 questionnaires)? Do these differ between both study arms?
- SO3d: To determine the incremental Net Monetary Benefit of incorporation of FDG-PET/CT with respect to QALYs (based on EQ-5D-5L index and overall survival) saved including sensitivity analysis.
- SO3e: To determine the incremental Net Monetary Benefit of incorporation of FDG-PET/CT with respect to decrease in unbeneficial treatment. Sensitivity analysis will be performed. A mere description will be given as there is no “accepted” value for this kind of analysis.

2.2.4 Secondary Objective 4: Cyto-, Histopathological and Tumorgenetic markers

- SO4a: Are there potential protein- or gene-expression profiles, capable of determining the nature of the FNAC-indeterminate nodes (cytology).
- SO4b: What is the interaction/correlation between the parameters mentioned in SO4a and the results of the FDG-PET/CT scan and the final diagnosis?
    - Can these tissue molecular biomarkers help in selecting the patients that benefit most from FDG-PET, or vice versa?
    - Can higher pre-operative diagnostic accuracy be achieved by combining FDG-PET and molecular biomarkers?
    - Are molecular biomarkers related to false-positive or false-negative FDG-PET/CT results?

3. STUDY DESIGN

A partially-blinded, prospective, multi-centre, block-randomised controlled trial (RCT) is designed. After obtaining written informed consent, patients are registered centrally, FNAC is reviewed centrally and thereafter patients are randomised to either the experimental study-arm (arm A: FDG-PET/CT driven management decisions) or a control study-arm (arm B: surgery regardless FDG-PET/CT result) using a 2:1 allocation ratio. The latter is performed both for methodological purposes (the treatment of the subjects in the control study arm is well investigated and described before) and to satisfy the assumption that patient prefer the experimental study-arm. The result of randomization is blinded to the treating physicians and central FDG-PET/CT review committee. Allocation is adaptively stratified using the minimisation method on the centre of inclusion and the most prognostic features of DTC known at this stage of patient work-up: (US) lesion size (0-10 mm/11-20 mm/21-40 mm/40+ mm), patient sex, patient age (<45yr, ≥45yr) and Bethesda classification according to central review committee (atypia of unknown significance/follicular lesion of unknown significance (III) or (suspicion) for a follicular neoplasia (IV)).

Based on the study arm allocation and PET result, treating physicians will either be told to cancel scheduled diagnostic thyroid surgery (allocation to the experimental arm with a negative FDG-PET/CT at central review or ~32/132 patients) or to proceed with surgery (all other cases or 100/132 patients). In case surgery is cancelled the patients need to be followed-up by watchful waiting and a confirmatory ultrasonography (including FNAC) after 12 months. Patient allocation will not be disclosed to prevent study withdrawal, however in case surgery is cancelled unblinding is unavoidable (~25% of all included patients).

The Gold Standard is histology, revised by a (central) dedicated pathologist panel, obtained from surgery or 12-months follow-up, including US (+FNAC). All patients will be followed for at least 12 months after FDG-PET/CT to ensure all direct costs and complications are noted. Five years after inclusion of the last patient, long-term follow-up will be determined based on patient file review and contact with the general practitioner (flowchart Figure 1, p34 and

Table 2, p35). To ensure study results are representative for clinical use and to improve inclusion-rates, this study will be performed in both university and regional hospitals (Table 1, p33, §15.1, Appendix I: Participating Centres and Site Managers, p89 and §15.2, Appendix II: Signed Document of Cooperating Centres, p90).

Centre:	Estimated Eligible patients
Amsterdam MC, Amsterdam	~20-30
Erasmus MC, Rotterdam	~24
Leiden University MC, Leiden	~15-25
Maastricht University MC, Maastricht	~15
RadboudUMC, Nijmegen	~20-25
University MC Groningen, Groningen	~20-25
University MC Utrecht, Utrecht	~12-24
Vrije Universiteit MC, Amsterdam	~13-19
Meander MC, Amersfoort	~25
Hagaziekenhuis, Den Haag	~10-15
OLVG Lucas Andreas, Amsterdam	~10-15
Rijnstate, Arnhem	~10-15
St. Antonius, Nieuwegein	~10-15
Isala, Zwolle	~10
Reinier de Graaf Gasthuis, Delft	~10
**Total:**	**~254**

Table 1: Participating hospitals and their estimated number of eligible patients

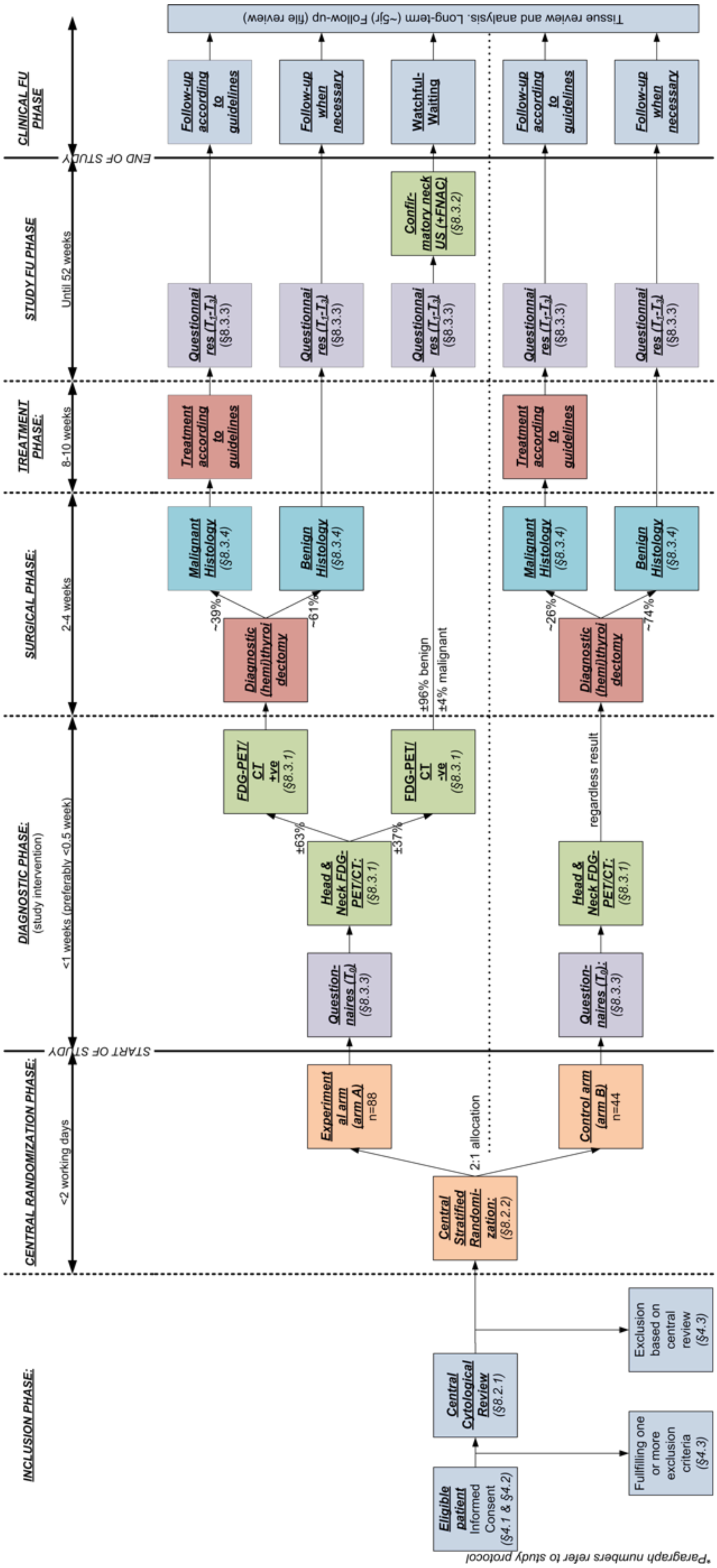

Figure 1: Flow-chart of the trial

**Procedure:**	**Before inclusion**	**During Investigation**		**Follow-up**				
		Arm A.	*Arm B.*	*direct*	*3mo*	*6mo*	*12mo*	*1-5yr*
History:								
Medical History	√						√[2]	(√)
Pregnancy status[1]	√							
Physical Examination:								
Complete clinical examination including palpation of thyroid gland and cervical lymphnodes	√						√[2]	(√)
Additional investigations:								
Laboratory (incl. TSH/fT4)	√							(√)
US of thyroid and cervical lymphnodes	√						√[*,2]	(√)
FNAC	√						√[*,1,2]	(√)
Central Cytology Review	√*							
Thyroid scintigraphy	√[1]							(√)
Blinded H&N FDG-PET/CT		√*	√*					
Additional test[4]	-	-	-	-	-	-	-	(√)
Treatment:								
Diagnostic Surgery		√[3]	√					
Cryopreservation of sperm		√[1,3]	√[1]					
Therapeutic Surgery + RIA		√[1,3]	√[1]					
Histopathology		√[3]	√					
Other:								
Sick leave recall					√*			
HRQoL		√*	√*	√*	√*	√*	√*	
Tissue Analysis		√*	√*					
Genetic Analysis		√*	√*					

Table 2: Summary of Protocol Investigations/Procedures. FNAC: fine-needle aspiration cytology; fT4: free thyroxine; FDG-PET/CT: Positron Emission Tomography using FDG of head, neck and upper thorax; RIA: radio-iodine ablation using ^{131}I; TSH: thyroid stimulating hormone; US: ultrasonography; [1]only on indication; [2]only in FDG-PET/CT negative patients in arm A; [3]only in FDG-PET/CT positive patients in arm A, [4]Additional tests such as gene-expression classification, mutational marker panel, US elastography are allowed in the mid- to longterm follow-up at the instigation of the treating physician (but are not reimbursed by the study funds). [*]Part of study protocol, reimbursed by study funds.

4. STUDY POPULATION

4.1 Population (base)

All adult patients that undergo cytological evaluation of a thyroid nodule in one of the hospitals recruiting patients for this multicentre trial are potentially eligible. Feasibility taking into account the prevalence of the disorder under investigation is described in §4.5, p40.

4.2 Inclusion criteria

In order to be eligible for participation, a subject must meet ALL of the following criteria:

- Documented history of a solitary thyroid nodule or a dominant nodule within multinodular disease, with (*US-guided)* FNAC performed by a dedicated radiologist or experienced endocrinologist or pathologist, demonstrating an indeterminate cytological examination (i.e. Bethesda category III or IV, §15.3, p91) according to the local pathologist and confirmed after central review (§8.2.1, p46);
  - If a core needle biopsy was performed instead of a FNAC, the patient is eligible if the biopsy procedure was felt to be minimally disruptive to the nodule architecture, based on a review by the principal investigator or nuclear medicine investigator.
- Scheduled for surgical excision (preferably) within 2 months of the inclusion date;
- Age ≥ 18 years;
  - This disease is rare in children and therefore the study will be limited to adults.
- Euthyroid state with a serum TSH or a free T4 level within the institutional upper and lower limits of normal, measured within 2 months of registration. In case of a suppressed TSH: a negative ^{123}I, ^{131}I or $^{99m}TcO_4^-$ scintigraphy must be available (“cold nodule”);
  - Mild deviations from the institutional normal limits may be considered acceptable if the patient has achieved a clinically euthyroid state with medication at a stable dose for >3 months, and the TSH is considered to be at target by the patient's treating physician. In patients with hyperthyroidism requiring treatment, this euthyroid state may be achieved with administration of a thionamide prior to FDG-PET/CT exam.
  - Patients with active (according to treating physician) hyperthyroid inflammatory conditions such as thyroiditis (e.g. m. Graves, Hashimoto, deQuervain, etc.) and toxic multinodular goitre often exhibit increased glucose uptake resulting in diffuse uptake of FDG which may obscure visualization of a thyroid tumour. These patients are not eligible for participation due to an expected higher false-negative rate.
- In patients with multinodular disease and a dominant nodule, the nuclear medicine physician responsible for FDG-PET/CT scan interpretation must determine whether the nodule is likely to be discriminated on FDG-PET/CT imaging prior to enrolment;
- Willing to participate in all aspects of the study;

  - confirmed by a signed and dated written informed consent obtained from the patient (or the patient's legally acceptable representative) prior to study participation.

4.3 Exclusion criteria

A subject who meets ANY of the following criteria will be excluded from participation:

High a priori probability of malignancy:

- FNAC Bethesda category V or VI (§15.3, p91) during local reading or central review (§8.2.1, p46);
- Prior radiation exposure / radiotherapy to the thyroid;
- Prior neck surgery or radiation that in the opinion of the PI has disrupted tissue architecture of the thyroid;
- New **unexplained** hoarseness, change of voice, stridor or paralysis of a vocal cord;
  - In case a benign reason has been found (e.g. vocal cord oedema), the patient is eligible;
- Initial diagnosis of the thyroid nodule through the coincidental discovery of a FDG-avid thyroid incidentaloma on PET-scan;
- New cervical lymphadenopathy highly suspicious for malignancy;
  - In case malignancy is excluded, patient is eligible;
- Previous treatment for thyroid carcinoma or current diagnosis of any other malignancy that is known to metastasize to the thyroid [49];
- Known metastases of thyroid carcinoma;
- Known genetic predisposition for thyroid carcinoma:
  - Familiar Non-Medullary Thyroid Cancer (NMTC)
  - Familiar Papillary Thyroid Cancer (FPTC), assumed if ≥2 primary or 1 primary and ≥3 secondary criteria are met:
    - Primary criteria:
      - PTC in 2 or more 1st degree relatives
      - Multinodular hyperplasia in 3 or more 1st or 2nd degree relatives
    - Secondary criteria:
      - Age <33 year;
      - Multifocal or bilateral tumour
      - T4-tumours
      - Metastases (N1, M1).
  - Familiar Adenomatoid Polyposis Coli syndrome (FAP, Gardner syndrome, APC-gene mutations on chromosome 5q21)

- Morbus Cowden (PTEN mutation on chromosome 10q23.3)
- PTC / nodular thyroid hyperplasia / papillary renal tumours (linked to locus 1q21).

Proven benign disease or insufficient material for a cytological diagnosis:

- FNAC Bethesda category I or II (§15.3, p91) during local reading or central review (§8.2.1, p46)

Performance of additional diagnostic test(s) prior to inclusion (or during the course of the trial), (potentially) affecting patient management:

- Any additional diagnostic test performed prior to inclusion, provided that this test is not yet part of routine clinical care (e.g. molecular testing or mutation analysis of cytological material) and that its results might affect patient management or the patient's/referring physician's willingness to participate in the study (§8.2.1, p46).

Inability to undergo randomization:

- Any patient that will receive thyroid surgery for other reasons (e.g. mechanical or cosmetic complaints).

Inability to undergo treatment:

- Inability to undergo surgery in the opinion of the surgeon / anaesthetist.

Contra-indications for FDG-PET/CT:

- Patient has evidence of infection localised to the neck in the 14 days prior to the FDG-PET/CT scan;
- Inability to tolerate lying supine for the duration of an FDG-PET/CT exam (~10-15min);
- Poorly regulated diabetes mellitus (see next item);
- Hyperglycaemia at time of FDG injection prior to PET/CT (fasting serum glucose >200mg/dL [>11.1 mmol/L]);
  - The use of short-acting insulines within 4 hours of the PET scan is not allowed
- If female and fertile: signs and symptoms of pregnancy or a positive pregnancy test / breast-feeding; A formal negative pregnancy test is not obligatory
- (severe) claustrophobia. Low dose benzodiazepines are allowed

General contra-indications:

- Inability to give informed consent;
- Severe psychiatric disorder;

4.4 Sample size calculation

Based on the main objective, it is expected that FDG-PET/CT can reduce unjust treatment from ~73.1% (=136/186 benign lesions >10mm) to ~40.3% (=75/186 false-positive and false-negative lesions >10mm, §1.3, p19). Using G*Power version 3.1.9.2. (and validated using JMP from SAS version 9.0.1, STATA version 12.0SE and PASS version 2011), we computed using an α error probability of 0.025 (=0.05/2, Bonferroni correction for interim analysis) a power of 0.80 and an allocation ratio of N_A:N_B=2:1, we need to include at least 90 patients in total (one-tailed test) to establish this difference (60+30 patients in arm A and B respectively).

Since around 82.7% (=186/225, §1.3, p19) fulfil the size criterion, at least 111 patients (74+37 patients in arm A and B respectively) need to be included. Accounting for additional 15% data loss at the final end-point, 132 patients should be included (88+44 patients in arm A and B respectively) (Figure 2, p40).

This total number of patients could at best (assuming no data loss) discern a reduction of 26.6% unjust treatment in the whole population with an actual α error probability of 0.016 and a power of 0.80. This is based on a reduction from 74.2% (prevalence of benign disease in all 225 patients) to 47.6%. In the experimental arm (88 patients), where the number of false-negative PET is estimated to be ~1.3% (3/225), this number of patients should establish a false-negative rate of maximal 7.5% (actual α error probability of 0.0066 and a power of 0.80).

All analyses will be performed according to the intention-to-treat principle, meaning that analysis is based on the initial study-arm allocation and not on the treatment eventually received (e.g. a FDG-PET/CT negative patient in the experimental arm does not refrain from surgery and therefore actually is treated according to the control study arm).

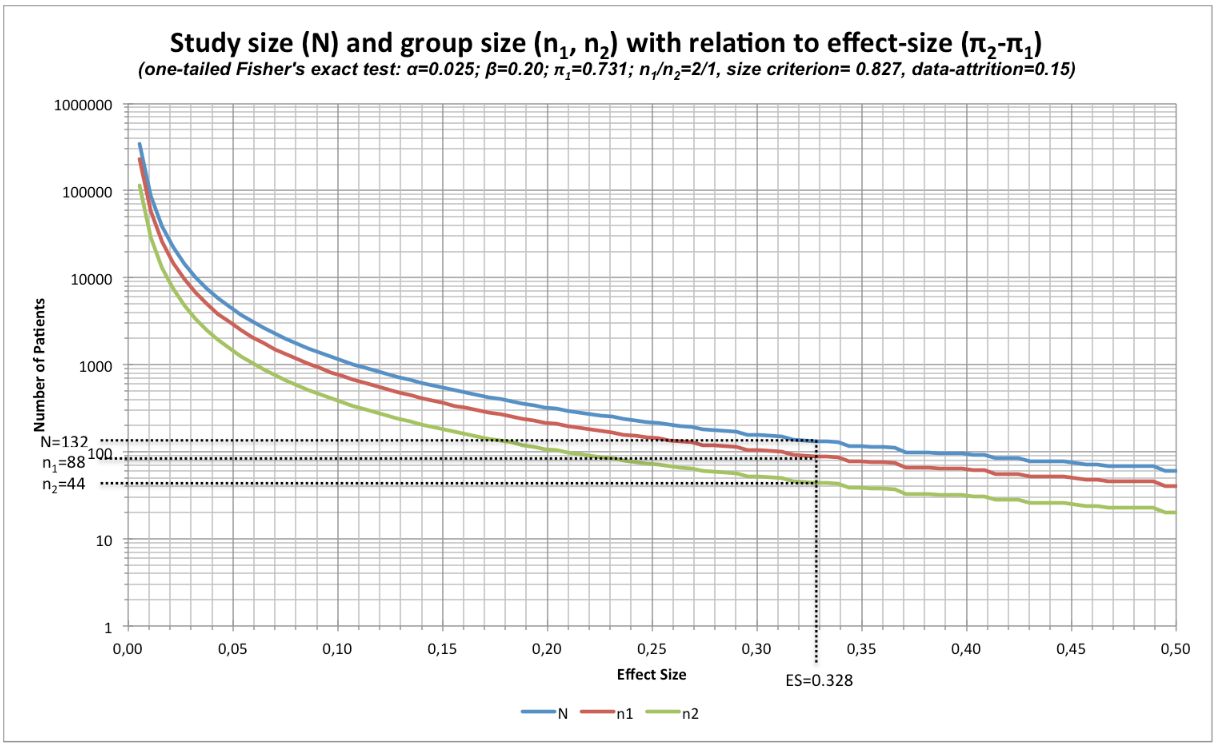

Figure 2: Relation between study size (N), study-arm size sizes (n_1 and n_2) and effect size. Empirical relation: $N=16.33935*ES^{-1.86986}$, $R^2=.999443$). Expected ES=73.1%-40.3%=32.8%

4.5 Feasibility

We expect to include 57 patients per year in all 9 centres (Appendix II: Signed Document of Cooperating Centres, §15.2, p90). This number is based on approximately 160-200 eligible patients per year in these centres (Table 3, p41) and a very conservative inclusion ratio of only 33% (also accounting for patients excluded based on secondary central review of cytology). Based on PALGA-data, around 750-1,000 patients per year are being confronted with a FNAC-indeterminate thyroid nodule, with these 9 centres we roughly expect to cover ~20% of all FNAC-indeterminate patients in the Netherlands.

As we target at a sample size of 132 patients, starting the inclusion of the first patients in June 2015, entering patients in the trial would take approximately 2.5 years (last patient included November 2017). As the patient follow-up is determined to be 12 months, the last data should then be collected November 2018. There are then 6 months remaining for unscheduled procrastination, data-analysis and reporting. Long-term follow-up will be performed no sooner than begin 2023.

With respect to randomization, based on clinical experience, we believe that patients would prefer not to undergo unbeneficial surgery as there is sufficient evidence that limited retardment of definite surgery in malignant disease has little clinical consequences. Therefore we used an allocation ratio of 2:1 of the experimental versus the control arm.

Addendum (20-02-2019):

Currently, we have included all 132 patients in all 15 centres. The final patient was included in November 2018. Patient recruitment and inclusion is now completed. In addition to the estimated number of eligible patients, Table 3 also shows the final number of included patients per centre. We discussed this observed one-year delay in patient inclusion with KWF, who generously granted us a budget-neutral prolongation of the trial until 01-11-2021. As the patient follow-up is determined to be 12 months, the last data should be collected in November 2019. This leaves two years for data analysis and manuscript preparations. Long-term follow-up will be performed no sooner than 2024.

Centre:	Est. eligible patients/yr:	Included nr. Patients:
Amsterdam MC, Amsterdam	~20-30	11
Erasmus MC, Rotterdam	~24	7
Leiden University MC, Leiden	~15-25	19
Maastricht University MC, Maastricht	~15	2
RadboudUMC, Nijmegen	~20-25	13
University MC Groningen, Groningen	~20-25	14
University MC Utrecht Utrecht	~12-24	5
Vrije Universiteit MC, Amsterdam	~13-19	23
Meander MC, Amersfoort	~25	18
HagaZiekenhuis, Den Haag	~10-15	4
OLVG Lucas Andreas	~10-15	6
Rijnstate, Arnhem	~10-15	2
St. Antonius, Nieuwegein	~10-15	3
Isala, Zwolle	~10	5
Reinier de Graaf Gasthuis, Delft	~10	0
**TOTAL:**	**~254**	**132**

Table 3: Estimated eligible patients and finally included number of patients per participating centre(Appendix II: Signed Document of Cooperating Centres, §15.2, p90).

5. TREATMENT OF SUBJECTS

This study is not an observational study as the (invasive) diagnostic route is dependent on an extra test performed (i.e. FDG-PET/CT).

5.1 Investigational product/treatment

An FDG-PET/CT is a widely available, routinely used diagnostic modality. As elaborated further in §8.3.1, p48, it requires limited preparation, administration of FDG does not result in any adverse events and the risks resulting from the radiation dose (~5 mSv for a whole-body investigation, 3.3-3.5 mSv for an average weighted adult head/neck FDG-PET/LDCT) are limited (see §15.6: Appendix VI: Radiation Ethics Form, p97). All involved centres have their own PET-CT/scanner.

All patients were scheduled for diagnostic thyroid surgery by an experienced surgeon prior to inclusion to this trial. In case of either a positive FDG-PET/CT in the experimental arm or inclusion in the control arm, scheduled surgery will be performed. In case of a negative FDG-PET/CT in the experimental arm, surgery will be cancelled and follow-up will be performed.

5.2 Use of co-intervention

As elaborated further, co-interventions include questionnaires (§8.3.3) and confirmatory ultrasounds of the neck after 12 months in a subset of patients (§8.3.2).

5.3 Escape medication

Not applicable.

6. INVESTIGATIONAL PRODUCT

6.1 Name and description of investigational product(s)

FDG-PET/CT is a routinely used diagnostic modality. It is used for many oncological and non-oncological purposes, including DTC. We do not investigate a new or non-authorised form. Therefore we do not consider FDG-PET/CT an 'investigational product'.

6.2 Summary of findings from non-clinical studies

Not applicable.

6.3 Summary of findings from clinical studies

See §1.3, p19 and our published meta-analysis [4].

6.4 Summary of known and potential risks and benefits

See chapter 13, p78 for a structured risk analysis including all study details.

6.5 Description and justification of route of administration and dosage

Not applicable.

6.6 Dosages, dosage modifications and method of administration

FDG-doses will be given according to the EANM guidelines and are dependent on (age), bodyweight, PET-scanner sensitivity (including 2D/3D acquisition) and scan-speed (minutes per bed position, overlap-percentage), §8.3.1, p48 [50]. Every institution can use its own optimised dosing and acquisition protocol.

6.7 Preparation and labelling of Investigational Medicinal Product

FDG is commercially available throughout multiple vendors in the Netherlands (e.g. BV Cyclotron, VU, Amsterdam; IBA-molecular; GE Healthcare).

6.8 Drug accountability

Not applicable.

7. NON-INVESTIGATIONAL PRODUCT

No non-investigational medicinal product is used in the study: no challenge agents nor products used to assess end-points in the trial are used. No medicinal product such as food products or a chemical compounds or stable isotopes or other products are used.

7.1 Name and description of non-investigational product(s)

Not applicable.

7.2 Summary of findings from non-clinical studies

Not applicable.

7.3 Summary of findings from clinical studies

Not applicable.

7.4 Summary of known and potential risks and benefits

Not applicable.

7.5 Description and justification of route of administration and dosage

Not applicable.

7.6 Dosages, dosage modifications and method of administration

Not applicable.

7.7 Preparation and labelling of Non Investigational Medicinal Product

Not applicable.

7.8 Drug accountability

Not applicable.

8. METHODS

8.1 Study parameters/endpoints

8.1.1 Main study parameter/endpoint

The fraction of unbeneficial treatment (i.e. surgery in benign disease plus watchful waiting in malignant disease) in both study arms based on intention-to-treat.

8.1.2 Secondary study parameters/endpoints

Secondary treatment outcome parameters (SO1, §2.2.1, p30):

- Complication fraction
  - Complications include: bleeding (requiring intervention or prolonging admission), infection (requiring intervention or prolonging admission), re-operation, ICU-admission, (temporary) hoarseness, (temporary) thyroid hormone suppletion or calcium/vitamin D suppletion.
- FDG-PET/CT diagnostic accuracy,
  - True-positive
  - True-negative
  - False-positive
  - False-negative
- Fraction of extra diagnostics due to coincidental FDG-PET/CT-findings,
- (Overall Survival and Disease Free Survival determined when last study-related follow-up of last included patient has been completed),
- Description of factors hampering implementation,
- As this study is performed intention-to-treat: the fraction and reasons of patients in the experimental study arm that still want a hemithyroidectomy despite a negative FDG-PET/CT scan.

Secondary HRQoL outcome parameters (SO2, §2.2.2, p30):

- Personal assessment in all domains of the EQ-5D-5L, the RAND-36 v2.0 (validated translation of the SF36-II), the ThyPRO and the Distress Thermometer (without the problem list) (including temporal relation),
- From the utility (EQ-5D-5L index) and overall survival, the number of QALYs are determined.

<u>Secondary costs and cost-effectiveness parameters (SO3, §2.2.3, p31):</u>

- Volumes of care including hospitalization days,
- iNMB (using QALYs saved as effect) including sensitivity analysis,
- iNMB (using unbeneficial treatment prevented as effect),
- Results of the iPCQ and iMCQ domains, including sick leave days.

<u>Secondary tissue (nodule) parameters (SO4, §2.2.4, p31):</u>

Performed on all (benign and malignant) resected lesions:

- US features (size, border, halo, calcifications, etc.)
- FDG-PET/CT features (SUV_{max}, SUV_{peak}, SUV_{mean}, metabolic volume, TLG)
- Cytological morphological features (Bethesda criteria, atypia, etc.)
- Histological morphological features (diagnosis, invasiveness, stage, atypia, etc.)
- Cytological and histological immunohistochemical features ((semi)quantitative: e.g. GLUT, HK, CA-IX, HIF-1α, Ki67/MIB, caspase-3, galectin-3, Tg, CD3, CD31/34)
- Cytological (tumour)genetic features and biomarkers: genetic profiling for gene alterations (mutations, fusions, rearrangements), gene expression, miRNA expression, and copy-number variations using next-generation sequencing and/or multigene classifier.

8.1.3 Other study parameters

Parameters such as age, sex, relevant history, medication, thyroid history, physical examination, laboratory results (thyrotropin, thyroid hormone levels, serum calcium, parathyroid hormone, glucose, thyroglobulin) and results of additional investigations (extra ultrasonography, FNAC cytology, investigations for coincidental PET-findings, etc.). These will be retrieved by examination of the patient files.

8.2 Pre-inclusion study procedures

8.2.1 Central Cytology Review

It is known that cytological classification of thyroid nodules is highly dependent on both observer and volume (experience), especially when they are classified as being indeterminate. The largest series reported, concluded that secondary cytological review can decrease the indeterminate rate from 38% to 28% [51]. Based on these results, we opt to homogenize our patients by central revision of cytological material by (consensus of) two experienced pathologists based on the Bethesda criteria (Appendix III: The Bethesda System for Reporting Thyroid Cytopathology, §15.3, p91). Based on their final decision, a patient will continue for randomisation in the trial or will be (secondarily) excluded. Besides that, a patient will be

excluded from participation in the trial if any additional diagnostic test (such as for mutation analysis or other additional tests that are not yet part of generally accepted, routine clinical care) were performed on the cytological material prior to inclusion and/or randomization of the patient (during local reading of cytology), as these test results might affect patient management and the patient's/referring physician's willingness to participate in the trial. Furthermore, as all tissue needs to be collected in a tissue-bank for immunohistochemical and genetic analysis, special requirements are needed for tissue collection and storage. These procedures will be described in SOPs (Standard Operating Procedures). As inclusion is based on the cytological result, all tissue from patients in whom a FNAC is performed for a (new) nodule should be collected (and stored) according these requirements, until the point that exclusion is certain (at latest after the central cytology review).

8.2.2 Randomisation, stratification, blinding and treatment allocation

The prognosis of thyroid carcinoma is mainly determined by Metastasis, patient Age, Completeness of resection, local Invasion, and tumour Size (MACIS [52]). Apart from these properties, sensitivity of FDG-PET/CT is mainly determined by nodule size and specificity is mainly deteriorated by follicular origin of the nodule. Only age, nodule size (on ultrasonography), cytological subtype and sex are known at the time of inclusion (Table 4, p48). Recently it was described that lesions larger than 40 mm (T3) carry a higher risk of malignancy compared to smaller lesions (odds ratio: 2.10, 95%-CI: 1.26-3.50) and that men have a higher risk of malignancy than women (odds ratio: 1.51, 95%-CI: 1.20-1.83) [53].

We will use a minimisation strategy [54] to stratify for centre (site) of inclusion, US size, Bethesda subtype, Age and Sex only, meaning that in case patients are homogenously distributed between both treatment arms over all these four factors, 2:1 randomisation will take place, in all other cases, patients will be allocated to the arm in which this type of patient is underrepresented.

Patients will be randomised in a 2:1 fashion to the experimental and control study arm. Patients, treating physicians, central pathologists and nuclear medicine physicians will be blinded for allocation, however in case of an FDG-PET/CT negative nodule in the experimental study arm, patient and treating physician are unblinded as surgery needs to be cancelled. In all other cases, patients proceed to surgery, but patients and treating physicians are kept blinded with respect allocation of the patient (either FDG-PET/CT positive nodule in the experimental study arm or inclusion in the control study arm regardless of PET-result).

Factor:	Level 1:	Level 2:	Level 3:	Level 4:
**Ultrasonographic Nodule Size:**	T1a (0-10mm) (13%)	T1b (11-20mm) (37%)	T2(21-40mm) (37%)	≥T3 (>40mm) (13%)
**Bethesda Subtype**	III (AUS/FLUS) (67%)	IV (SFN) (33%)		
**Age**	<45yr (40%)	≥45 yr (60%)		
**Sex**	Female (80%)	Male (20%)		

Table 4: Stratification during study including expected fractions (based on [4] and [51]). AUS/FLUS: Atypia of Undetermined Significance or Follicular Lesion of Undetermined Significance; Rep: repeatedly; SFN: (Suspicious for) Follicular Neoplasm.

8.3 Study procedures

8.3.1 FDG-PET/CT of head and neck

A single FDG-PET/CT of the head and neck without a contrast-enhanced CT is performed in all patients in the local centre according to the EANM guidelines [50], independent of allocation results. The scanned area ranges from meatus acusticus externus to the fossa jugularis sternalis (or down to the lower margin of the arcus aortae in case of retrosternal goitre), with arms down and preferably using a head rest (Figure 3, p49). Only a section of the body is scanned as the possibility that a single (PET-positive) lesion outside the thyroid bed is the only evidence of DTC is extremely rare and decreasing the scan range will decrease the effective dose of the investigation. Most of these findings are supposed to be coincidental findings unrelated to DTC and harbour a very low a posterior probability to be of serious consequences, could be considered as “screening a healthy population using FDG-PET” which is highly controversial and probably will lead to increase use of diagnostics and expenditure of public funds and could demotivate physicians in including patients in such a trial. E.g. findings of a positive lesion in the colon would certainly be unrelated to DTC, might lead to endoscopic examination which are costly, could lead to complications for the patient, have a low probability to lead to a serious medical condition requiring therapy and would finally demotivate the physician to include more patients.

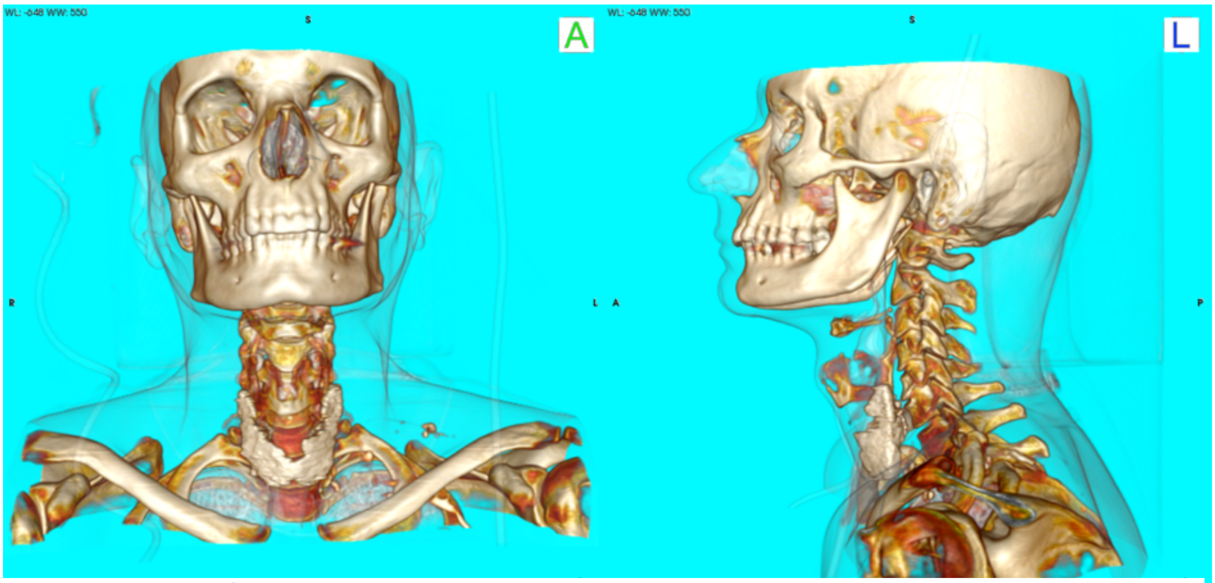

Figure 3: Position of the normal thyroid.

As an example guideline, in short: patients are asked to refrain from eating and drinking for 6 hours, only water and medications are allowed. In insulin-dependent diabetics, insulin levels should be as low as possible, while maintaining normoglycemia (serum fasting glucose <200 mg/dL [<11.1 mmol/L]). No (short-acting) insulin may be injected in the 4 hours before FDG-infusion to reach normoglycemia.

At the department of nuclear medicine, patients are prehydrated using 500 mL of tap water orally. An i.v. cannula is placed in a peripheral vein and about 3.45 MBq/ kg (<25% bed overlap) or 1.725 MBq/ kg (50% bed overlap) FDG (based on 3D acquisition, 4 min/bedpost) is injected intravenously after which the cannula can be removed. This administered FDG-dose may be adapted to local standards as long as these do not violate the current EANM guidelines [50, 55]. Adaptation to this those can be performed incorporating factors such as scanner sensitivity, scan speed and bed overlap.

The patient is incubated alone in a supine position for 60 minutes (+/- 5 minutes) in a warmed (thermoneutral) environment and asked to refrain from muscle activity (no talking, etc.). In case of severe claustrophobia, the use of a benzodiazepine (e.g. oxazepam 10 mg) is allowed.

After incubation, the patient is positioned in the scanner using a head rest to prevent motion mismatch between PET and CT. After a scout view (Figure 4, p50) and a low-dose, non-contrast enhanced CT, PET-acquisition of the head and neck is performed for at least 2 min per bed position. Two bed positions are usually enough to cover the range from the meatus acusticus externus to the fossa jugularis sternalis (or down to the lower margin of the arcus aortae in case of retrosternal goitre).

After the PET-acquisition, the scan is complete and the patient can go home without any further restrictions. The total examination takes around 1.5 hours. Images will be reconstructed using the departments own protocol, including corrections for isotope decay, attenuation, scatter,

dead time and are cross-calibrated to the dose-calibrator of the FDG-dose and in conjunction with the EANM guidelines [50, 55]. The reconstructed FDG-PET/CT images must not be reported and are digitally pseudonymised and submitted for central reading. The results of this examination will be withheld from the patients and treating physicians. Patients are eligible for compensation of the travelling expenses.

FDG-PET/CT is an established (and for a number of other indications proven cost-effective) procedure for multiple oncological and non-oncological issues, covered by the Basic Insurance Package. FDG-PET/CT is a relative non-invasive procedure with only minimal risks attached to it (see §13.1.1: FDG-PET/CT, p78). Administration of FDG does not result in any adverse events. An FDG-PET/CT scan is widely available in almost all hospitals in the Netherlands and can usually be performed within 3 working days. A standard operating procedure (SOP) will be available.

The use of different acquisition protocols (for different departments and different PET-scanners) causes heterogeneity in image quality. This probably deteriorates the power of this study, but at the same time will improve applicability of the results for clinical practice. The central PET-reading committee of this study has experience with multiple models of PET-scanners.

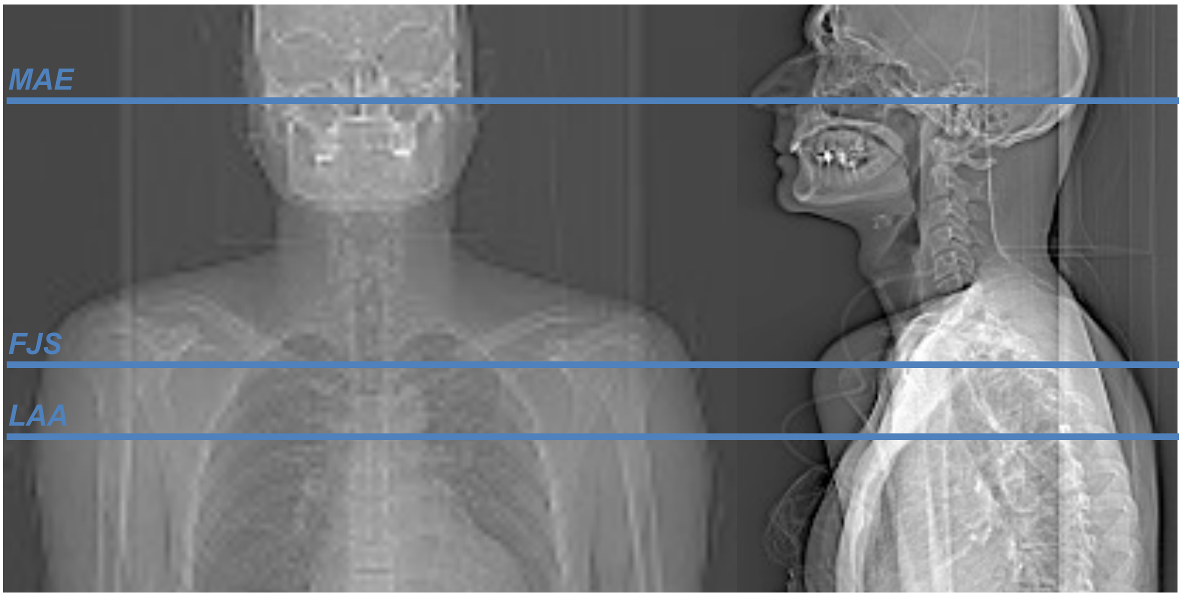

Figure 4: Topograms showing the major landmarks: the meatus acusticus externus (*MAE*), the fossa jugularis sternalis (*FJS*) and the lower margin of the arcus aortae (*LAA*).

Definition of positive and negative FDG-PET/CT:

A positive FDG-PET/CT is defined as focal FDG-accumulation at the site of the FNAC-indeterminate thyroid nodule higher than (thyroid) background. All other cases are considered FDG-PET/CT negative. For example, see Figure 5, p51. In case of diffuse thyroid FDG-uptake (thyroiditis), the same definition is used, but the higher background should be noted.

The treating physician should receive written notification of the consequences of this result (surgery or not) within one week of the FDG-PET/CT. No disclosure of PET-result of the thyroid nodule (positive or negative) or the study arm allocation should be given.

Contralateral incidentalomas:

In case of an extra FDG-PET/CT-positive thyroid lesion *contralateral* to the thyroid nodule under investigation, it is considered an incidentaloma and worked-up accordingly (~35% harbour malignancy [56]) before a *ipsilateral* hemithyroidectomy is performed. This is not necessary in case total thyroidectomy is scheduled.

Relevant coincidental findings:

In case of clinically relevant coincidental findings (e.g. suspicion of a head & neck carcinoma, oesophageal carcinoma, lung carcinoma or (FDG-avid) cervical or mediastinal lymphadenopathy) this should be reported to the treating physician in writing. Cervical lymphadenopathy without focal thyroid uptake needs further work-up (e.g. US+FNAC, MRI).

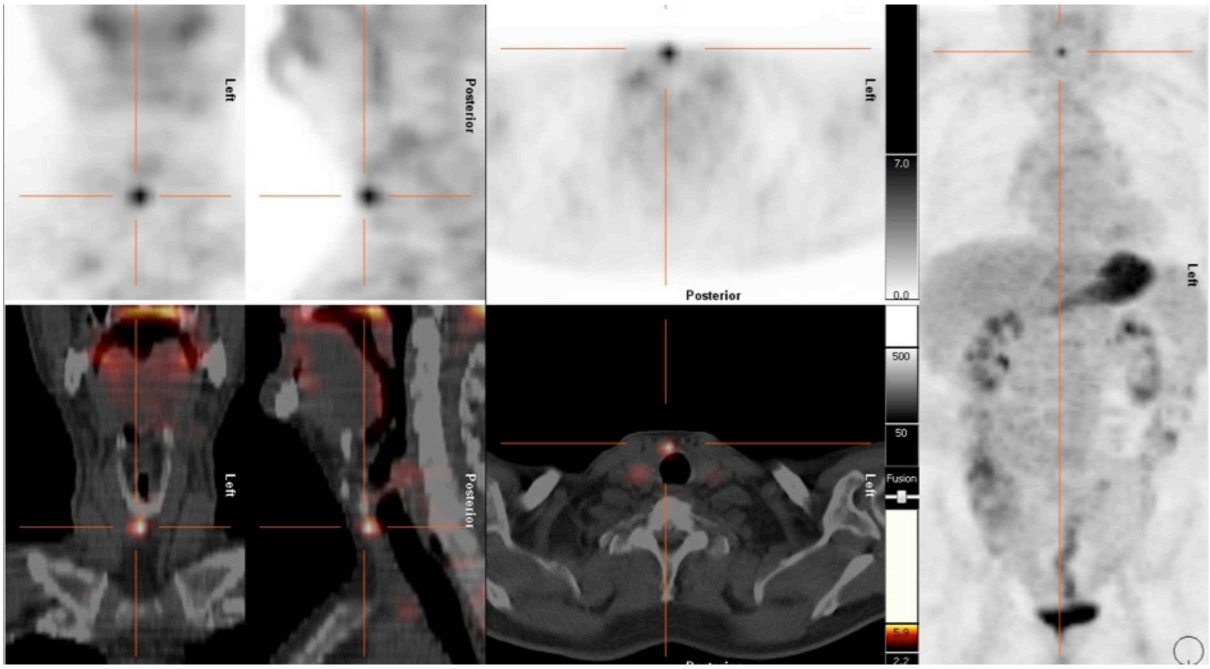

Figure 5: Example of a FDG-PET/CT positive thyroid nodule in the isthmus, which proved to be a 10 mm differentiated papillary thyroid carcinoma (pT1a cN0M0)

8.3.2 Confirmatory Neck US

A confirmatory US of the neck is performed locally by a dedicated head and neck radiologist in all FDG-PET-negative patients in the experimental arm, 12 months after inclusion. No preparations are needed. An ultrasound is performed in supine position after application of conducting gel. If nodule size is stable (i.e., no more than a 50% change in volume or <20% increase in at least two nodule dimensions in solid nodules or in the solid portion of mixed cystic–solid nodules), the interval before the next follow-up clinical examination or US may be longer, e.g., every 3–5 years. If there is evidence for nodule growth either by palpation or on US (more than a 50% change in volume or a 20% increase in at least two nodule dimensions with a minimal increase of 2 mm in solid nodules or in the solid portion of mixed cystic–solid nodules), the FNAC should preferably be repeated (on discretion of the physician performing the US), preferably under US guidance: the neck will be cleaned with alcohol and a fine-needle will be inserted into the lesion, causing no more pain than a regular venapunction. Other indication for FNAC are also on discretion of the physician performing the US, but should take into account prior US results. The patient is treated further according to the guidelines [5, 6]. Afterwards the patient can go home without any further restriction. The total examination takes around 0.5 hours. The results of the confirmatory US are known by the treating physician, PI and the patient is informed (i.e. no blinding). Patients are eligible for compensation of the travelling expenses.

8.3.3 Questionnaires

All study patients will be asked to fill in 6 self-administered questionnaires in their native language at baseline, after 3 months (or directly after surgery+^{131}I-RIA), after 6 months and after 12 months. Together these 6 questionnaires take about 30-45 min to fill in:

- RAND-36 V2.0 (SF36-II): The RAND-36 V2.0 is a validated translation in Dutch of the Short Form health survey (SF36-II) comprising 36 questions yielding a 9-scale profile of function health, wellbeing and psychometrically based physical and mental health [57]. It takes approximately 5 minutes to complete. As the SF36-II was not originally designed for use in economic evaluations or to determine QALYs, the SF-6D can be created by applying a scoring method, focusing on seven of the eight health domains of the SF36-II [58] (Appendix VII: , §15.7 p98).
- EQ-5D-5L: EuroQol 5-dimensional 5-level questionnaire: indexing health status, 2 pages including a single visual analogue scale (VAS) [59]. It takes approximately 5 minutes to complete (Appendix VII: , §15.7 p98)..

- iPCQ: Productivity Costs Questionnaire, 12 questions on health and losses of productivity in paid and unpaid labour [60]. It takes approximately 5 minutes to complete (Appendix VII: , §15.7 p98).
- iMCQ: Medical Consumption Questionnaire, indexing direct costs of self-reported health care consumption through 35 short questions. It takes a maximum of 20 minutes to complete [61].
- ThyPRO: Thyroid Patient-Reported Outcome, a disease specific 13-scale 84-item questionnaire designed for benign thyroid disease, but being investigated for use in DTC. [62] It takes approximately 14 minutes to complete (Appendix VII: , §15.7 p98).
- DT&PL: Distress Thermometer: a 11-point VAS including a 35-item problem list [63]. Based on expert opinion (by dr. R. Netea-Maier, endocrinologist at Radboudumc), we will only use the VAS, since the 35-item problem list mostly overlaps with the content of the other questionnaires. The entire DT&PL takes approximately 5-10 minutes to complete. We estimate that the VAS itself will take a maximum of two minutes to complete. (Appendix VII: , §15.7 p98).

Questionnaires are returned to the PI either by mail or email.

8.3.4 Cytology and histopathology analysis

In the study, cytopathology and histopathology specimens are collected. After cytopathology is collected from individual patients during FNA (prior to study inclusion), cytopathology slides are reviewed at the Radboudumc by two experienced thyroid pathologists to ensure appropriate inclusion in the study. If the first and second pathologist disagree, a third dedicated pathologist will be consulted. Consensus upon classification as either Bethesda category III or IV has to be reached before inclusion of a patient into the study (§8.2.1, p46). Cytopathology samples are returned and stored in local tissue banks at the centre where the FNA was initially performed. Before start of the study, a consensus meeting was held with the dedicated pathologists from the different centres to minimise interobserver variation.

After surgery, thyroidectomy specimens are being stored at the hospital where the surgery has been performed and will be collected at the end of the study. After surgery in all appropriate study patients is completed, histopathology specimens will be reviewed at the Radboudumc by the same experienced thyroid pathologists but an expert of the LUMC may be consulted for specific cases. Characteristics of the lesion like type of lesion, size, cell type, inflammation and vascularity will be scored (see §8.1.2, p45) in adherence to the World Health Organization guidelines [64].

Next, cytological and histopathological tissue characteristics will be analysed in relation to FDG-PET/CT findings in order to determine whether specific tissue characteristics can explain false positive or negative FDG-PET/CT results - like for instance high vascularity or cell type.

Definition of malignant and benign thyroid nodules:

A nodule is considered malignant, in case malignancy is diagnosed at the location of the nodule. All other cases are considered benign nodules.

Following advanced insights and in accordance with WHO guidelines, pre-malignant entities such as non-invasive follicular thyroid neoplasm with papillary-like nuclear features (NIFTP), follicular tumor of uncertain malignant potential (FT-UMP) and well-differentiated tumor of uncertain malignant potential (WDT-UMP) are distinguished. These are strictly considered benign though potentially precancerous lesions, for which thyroid lobectomy is justified [65, 66].

As such, a FDG-PET/CT scan is considered true positive if the nodule that was FDG-avid harbors any type of histopathology that warrants surgery, including malignancy, NIFTP, FT-UMP and WDT-UMP. If such a nodule is non-FDG-avid, it will be considered false-negative.

In case a DTC microcarcinoma (T1a or diameter ≤10mm) is found within a **much larger** nodule, this is considered a coincidental finding. The patient will be treated for DTC (in the special case of a papillary unifocal microcarcinoma, according to the guidelines one can suffice with a therapeutic hemithyroidectomy without completing thyroidectomy and ^{131}I-RIA [5]). In case this much larger nodule was positive on the FDG-PET/CT, it is considered false-positive; in case the nodule was negative on the FDG-PET/CT, it is considered true-negative.

Lesion-by-lesion qualification of thyroid nodules:

Five locations are considered (left/right lobe with upper/lower pole and central: isthmus/lobus pyramidalis: Figure 6, p54). Qualification of the PET result to be true or false is based on anatomically matched locations (e.g. an FDG-PET/CT positive lesion at the location of a malignant nodule is a true-positive result). In case in doubt (e.g. a node centrally in the left or right lobe), consensus reading by pathologist, surgeon and nuclear medicine physician will be performed.

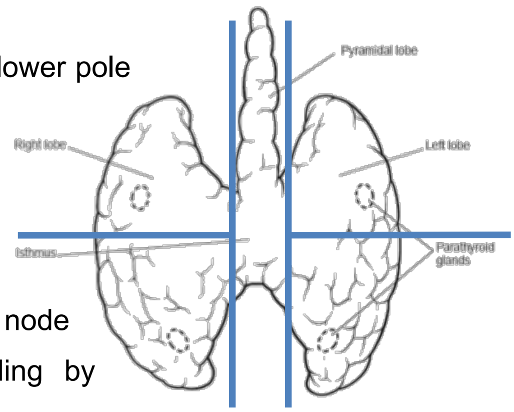

Figure 6: Anatomy and subdivision of the normal thyroid gland

Multifocality:

In case the patient receives surgery for a FDG-negative nodule (only in the control arm) but this nodule is found to contain malignant tissue, the patient is considered false-negative.

If, however, in the same patient a second malignant nodule is found in the contralateral thyroid lobe at completion thyroidectomy, which was positive on PET ("true-positive"), the patient is considered false-negative and thus the treatment following the PET-scan unbeneficial.

The reason for this is that it was not the FDG-PET/CT-scan that proved the indication for diagnostic surgery, as the lesion under investigation was considered negative, even though the patient is diagnosed correctly with DTC at surgery. In other words: if the patient would have been included in the experimental study arm, the false-negative lesion would have led to a follow-up policy and the contralateral malignancy would never have been diagnosed by a hemithyroidectomy of the site of the false-positive lesion (unless the work-up for a contralateral incidentaloma would have let to a true-positive result).

As stated before, PET-incidentalomas must be worked-up before surgery unless total thyroidectomy is planned as the initial procedure. Abovementioned rare case could therefore only occur if the contralateral PET-incidentaloma was found benign/unsuspicious at work-up or initial total thyroidectomy was planned.

8.3.5 Tissue genetic analysis

All cytology specimens will be genetically profiled using various molecular biomarkers. These tissue markers will include point mutations (e.g. BRAF and RAS point mutations), gene fusions and gene rearrangements (e.g. RET/PTC and PAX8/PPARγ rearrangements), gene and miRNA expression profiling, and copy-number variations, and possibly immunocyto- and immunohistochemical markers.

Testing techniques for molecular tissue biomarkers are rapidly developing globally. Nonetheless, as mentioned in a previous paragraph, these techniques are not yet available at Radboudumc (§1.10 Laboratory infrastructure and available techniques).

We will perform molecular diagnostics at the Leiden University Medical Centre (LUMC) (at the pathology lab of prof. dr. J. Morreau) using state of the art next generation sequencing techniques and biomarker panels. For this purpose, cytology slides from all study participants will be sent to the LUMC. Informed consent was obtained from the study participants for these analyses.

Molecular testing on cytology requires the extraction of ±20ng of DNA/RNA from the existing cytology slides. To extract sufficient nucleotides, part of the cytological material will be scraped of one or two cytology slides from each patient. Prior to DNA/RNA extraction, all cytology slides

will be scanned and digitally stored; these images will be made available to and saved at the hospital where the FNA was performed, in order to enable any desired morphological review of cytology in the future. This method is also regularly used in a clinical setting. Destruction of (parts of) cytology slides disables any further staining, immunocytochemistry or other cytology testing in the future. However, as cytology review has already been completed at that point in the current study and the individual patient's treatment has been determined and executed (i.e. most patients will have undergone surgery), this will have no consequences for individual patient management.
If appropriate, some cytology and histology samples may additionally be selected for further molecular testing at LUMC. This may e.g. concern Hürthle cell lesions and any copy number variations detected.

Obtained genetic profiles will be matched to the histopathological diagnosis and characteristics, to (pre-operative) clinical characteristics and to the results of the FDG-PET/CT scan, in order to evaluate underlying associations. Moreover, we would like to evaluate potential additional (molecular) tests that could enhance FDG-PET/CTs specificity (and sensitivity).

Results of the tissue genetic analysis on cytology are not used to determine patient management, but solely for investigative/explorative purposes. However, if the result of the gene expression classifier or any additional genetic testing would show a high or definitive risk of malignancy, in a patient that did not undergo diagnostic surgery (i.e. the patient was randomized to the experimental study arm with a negative FDG-PET/CT (possible false-negative) and was advised watchful-waiting instead of surgery, or the patient chose not to undergo surgery for other reasons), the patients' treating physician will be informed of the genetic results and advised to undertake appropriate action (i.e. further diagnostics or advise to undergo surgery).

8.4 Withdrawal of individual subjects

Subjects can leave the study at any time for any reason if they wish to do so without any consequences. The investigator can decide to withdraw a subject from the study for urgent medical reasons.

8.4.1 Specific criteria for withdrawal (at the end of the study)

The false-negative FDG-PET/CT ratio is expected to be around 1.3%, but we accept a false-negative ratio of up to 5% (i.e. the a priori risk of malignancy in any thyroid nodule). After

inclusion and surgery of all 44 patients in the control study-arm, final analysis will be performed. In case 6 or more of all these 44 patients (π_1*=13.6%) in the control study-arm have a false-negative FDG-PET/CT, the treating physicians of all PET-negative patients from the experimental study arm will be advised to proceed to diagnostic (hemi)thyroidectomy. This number is based on the cumulative binomial distribution, by solving:

$$\sum_{n=X}^{n=N} \binom{N}{X} \cdot p^X \cdot (1-p)^{N-X} \leq \alpha$$

With N=44, p=0.05 and α=0.025 (Bonferroni-adjusted), resulting in X≥6. Single-sided testing was used. Figure 7, p57.

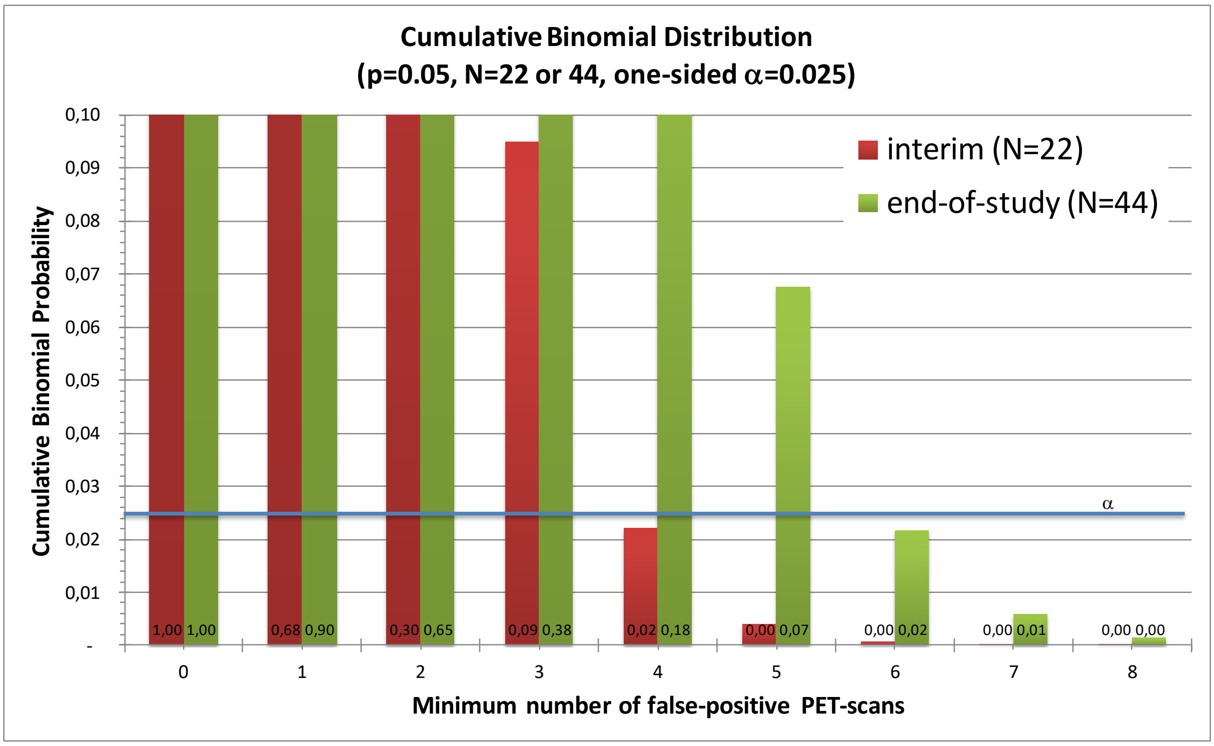

Figure 7: Cumulative Binomial Distribution for a sample size of 22 (interim-analysis) or 44 (end-of-study evaluation) for an individual patient probability of a false-negative FDG-PET/CT of 0.05. In case that more than 4/22 patients (18.2%) at time of the interim analysis or 6/44 (13.6%) patients at time of the end-of-study evaluation have a false-positive FDG-PET/CT (in the control study arm), the false-negative probability is larger than the maximum accepted value of 0.05.

8.5 Replacement of individual subjects after withdrawal

In case of secondary exclusion (e.g. based on central cytology revision), new patients can be entered into the study to a maximum of 15% (20 patients). This limit is set to the same value as the amount of maximum data-attrition for budgetary reasons.

Once a PET-scan has been performed, replacement is not possible. Sample size adjustments have been made for data-attrition (e.g. a patient is lost during follow-up). In case a patient cannot adhere to the protocol during any phase of the study (e.g. is considered inoperable or wishes thyroid surgery despite a negative PET-scan in the experimental arm), the reason is

noted, but patients do not switch study arm (i.e. intention-to-treat analysis and not per-protocol analysis).

8.6 Follow-up of subjects withdrawn from treatment

All patient files will be examined 12 months and ~5 years after inclusion (§8.8, p58)

8.7 Premature termination of the study (interim-analysis)

The false-negative FDG-PET/CT ratio is expected to be around 1.3%, but we accept a false-negative ratio of up to 5% (i.e. the a priori risk of malignancy in any thyroid nodule). After inclusion and surgery of 22 (50%) patients in the control study-arm, an interim analysis will be performed by the safety committee (§9.5: Data Safety Monitoring Board (DSMB) / Safety Committee, p62). In case 4 or more of these first 22 patients (π_1*=18.2%) in the control study-arm have a false-negative FDG-PET/CT, the study will be terminated preliminarily and the physicians of all PET-negative patients in the experimental study arm will be informed to advise them to undergo diagnostic (hemi)thyroidectomy. This number is based on the cumulative binomial distribution, by solving:

$$\sum_{n=X}^{n=N} \binom{N}{X} \cdot p^X \cdot (1-p)^{N-X} \leq \alpha$$

With N=22, p=0.05 and α=0.025 (Bonferroni-adjusted), resulting in X≥4. Single-sided testing was used. Figure 7, p57.

8.8 Mid- to long-term follow-up

The duration of short-term follow-up since FDG-PET/CT for this study is set at 12 months, as it is expected that most treatment procedures have taken place within this period and most complications have been resolved. Primary and secondary outcome measures are therefore collected during this one-year period.

In the ideal case that no false-negative FDG-PET/CT-scans occur, it is safe to assume that recurrences and other mid- to long-term sequelae would occur in same fractions in both study arms. However, a one-year follow-up period could be considered too short to conclude a false-negative-fraction for FDG-PET/CT as a delayed diagnosis of malignancy might be made over a year after initial FNAC as DTC usually follows an indolent growth. Also, the costs and utilities (health related quality of life) attributed to delayed treatment would not be completely incorporated in the study most likely leading to an underestimation of false-negative rates, an underestimation of costs, and an overestimation of utility in de experimental study arm due to censoring.

Therefore, five year after the inclusion of the last patient a retrospective study will be conducted using patient files completed with contact with the general practitioners of these patients to investigate the mid- to long-term effects of our intervention.

All treating physicians are aware that about 3.6% of all negative FDG-PET/CT scans are false-negative (1.3% of all patients included in the study). The short-term follow-up will distinguish a portion of these patients with thyroid malignancy, but probably not all. Therefore, the treating physicians are advised to follow-up the nodules for at least 5 years like “benign nodules” in the ATA-guidelines [6], recommendation 14:

(a) It is recommended that all benign thyroid nodules be followed with serial US examinations 6–18 months after the initial FNAB. If nodule size is stable (i.e., no more than a 50% change in volume or <20% increase in at least two nodule dimensions in solid nodules or in the solid portion of mixed cystic–solid nodules), the interval before the next follow-up clinical examination or US may be longer, e.g., every 3–5 years.

(b) If there is evidence for nodule growth either by palpation or sonographically (more than a 50% change in volume or a 20% increase in at least two nodule dimensions with a minimal increase of 2mm in solid nodules or in the solid portion of mixed cystic–solid nodules), the FNA should be repeated, preferably with US guidance.

The malignancy rate after a reassuring confirmatory US in these patients is less than 3.6%, which is similar to Bethesda cat. II (benign, malignancy risk 0-3%) or Bethesda cat. I (nondiagnostic or unsatisfactory, malignancy risk 1-4%).

Unlike with the US and FNA during the inclusion phase, with the (first) confirmatory US 12 months after a negative FDG-PET/CT (in the experimental study arm) the treating physician is free in using additional diagnostic tests such as a gene-expression classifier, mutational marker panel, US elastography, etc. Apart from this confirmatory US, all other investigations are outside the scope of this project.

9. SAFETY REPORTING

9.1 Section 10 WMO event

In accordance to section 10, subsection 1, of the WMO, the investigator will inform the subjects and the reviewing accredited METC if anything occurs, on the basis of which it appears that the disadvantages of participation may be significantly greater than was foreseen in the research proposal. The study will be suspended pending further review by the accredited METC, except insofar as suspension would jeopardise the subjects' health. The investigator will take care that all subjects are kept informed.

9.2 AEs, SAEs and SUSARs

9.2.1 Adverse events (AEs)

Adverse events are defined as any undesirable experience occurring to a subject during the study, whether or not considered related to the experimental intervention (e.g. an AE can also be related to a diagnostic procedure or to an already existing condition). All adverse events reported spontaneously by the subject or observed by the investigator or his staff will be recorded. All AEs are registered on electronic case report forms.

9.2.2 Serious adverse events (SAEs)

A serious adverse event is any untoward medical occurrence or effect that at any dose:

- results in death;
- is life threatening (at the time of the event);
- requires hospitalisation or prolongation of existing inpatients' hospitalisation;
- results in persistent or significant disability or incapacity;
- is a congenital anomaly or birth defect;

Any other important medical event that may not result in death, be life threatening, or require hospitalization, may be considered a serious adverse experience when, based upon appropriate medical judgement, the event may jeopardize the subject or may require an intervention to prevent one of the outcomes listed above.

In case of a SAE, the investigator of the participating centre is responsible for handling and reporting the SAE. All SAEs should be reported to the Study Coordinator within 24h hours of the event. The Study Coordinator will register the SAE and will report the SAEs through the web portal *ToetsingOnline* to the accredited METC that approved the protocol, within 15 days after the sponsor has first knowledge of the serious adverse reactions.

SAEs that result in death or are life threatening should be reported expedited. The expedited reporting will occur not later than 7 days after the responsible investigator has first knowledge of the adverse reaction. This is for a preliminary report with another 8 days for completion of the report.

Exception

A single exception is made to the notification obligation of SAEs: no notification of a SAE is made in case of prolongation of an existing inpatients' hospitalisation due to a postoperative hypocalcaemia, as (mild) hypocalcaemia is a fairly common and expected complication of thyroid surgery. It is usually easily corrected with medication and does not cause persistent damage to the patient. However, in case of severe, possibly life-threatening hypocalcaemia, requiring admission to the ICU or other profound or invasive interventions, a SAE notification will be made as stated above.

9.2.3 Suspected unexpected serious adverse reactions (SUSARs)

As no investigational medicinal product is used this chapter is not applicable.

9.3 Annual safety report

As no investigational medicinal product is used this chapter is not applicable.

9.4 Follow-up of adverse events

All AEs will be followed until they have abated, or until a stable situation has been reached. Depending on the event, follow up may require additional tests or medical procedures as indicated, and/or referral to the general physician or a medical specialist.

SAEs need to be reported till end of study within the Netherlands, as defined in the protocol

9.5 Data Safety Monitoring Board (DSMB) / Safety Committee

As this study comprises a clinical studies in non-critical indications where patients are treated for a relatively short time and the drugs/procedures under investigation are well characterised and known for not harming patients, no DSMB is needed according to the EMEA guidelines [67]. Furthermore, the risk attached to the investigation is considered negligible (see §13: STRUCTURED RISK ANALYSIS, p78).

A Safety Committee however will be established to perform interim analyses on the safety data. This committee is an independent committee and consists of:

- a statistician (Dr. A.R.T. Donders).
- a surgeon (Dr. J.J. Bonenkamp),
- an interventional radiologist (Prof. dr. J.J. Fütterer)

The Safety Committee will meet at least yearly during the course of the study, and will consider results of data-monitoring:

- recruitment figures and losses to follow-up presence and completion of research file
- informed consent (1-10%)
- adherence to in- and exclusion criteria (first 3 participants per centre, than 1-10%)
- Source Data Validation: 1-10% based on predefined variables including primary end-point, clearly related to safety and validity of the project
- missed (1-10%) and reported (S)AEs
- validation of which instructions are given to patients
- validation of availability of study procedures including SOPs
- verification of collection, labelling and storage of biological samples
- suggestion of additional data analyses
- Advise on protocol modifications suggested by investigators or sponsors (eg to inclusion criteria, trial endpoints, or sample size)
- Monitor planned sample size assumptions
- Monitor continuing appropriateness of patient information
- Monitor compliance with previous Safety Committee recommendations
- Considering the ethical implications of any recommendations made by the Safety Committee

Furthermore they will perform an independent interim-analysis (§8.7, p58) and a safety end-of-study analysis (§8.4.1, p56).

This report will be sent to the Study Coordinator, who will send this report to the CCMO including the planned action.
A more detailed description of the expected role and responsibilities of this Safety Committee is described in the charter.

Addendum 20-02-2019:
An interim analysis was performed on 17-04-2018. Results were reported to the Safety Committee, who approved continuation of the trial according to protocol (v1.4 at the time) with no adjustments.

10. STATISTICAL ANALYSIS

10.1 General remarks

All (statistical and economical) analyses will be supported by epidemiologists and biostatisticians from the LUMC.

All continuous data (e.g. age, FDG-PET/CT standardised uptake level (SUV), volumes of care, hospitalisation and sick leave days, Tg-level, questionnaire scores, etc.) will be assessed for (log)normality.

All categorical data (e.g. sex, FDG-PET/CT positive or negative, malignant or benign, histology, etc.) are treated as either ordinal or nominal.

One year is defined as 365.242199 days. Age, overall survival, disease-free-survival are computed with respect to the date of the (last) US guided FNAC that led to study enrolment. Survival is computed, using the Kaplan-Meier method, taking into account censored data (censored to the date of last follow-up or death (for all but overall survival)). For comparison of Kaplan-Meier estimates, the logrank statistic will be used (as the Breslow and Tarone-Ware tests are better used when the data does not fulfil the proportional hazards assumption; they are both weighted versions of the logrank test giving extra weight to events happening earlier on. The Tarone-Ware has slightly less extreme weightings. Both should be avoided in case of a high level of censoring or unequal censoring between groups).

All prices will be calculated based on national price levels (in Euro, €), indexed to the year of first patient inclusion (2015). Price-index-numbers will be used to account for price changes during the years of patient inclusion and follow-up. In case of costs distributed over several years, net present values are computed using a constant 4% discount rate and effects using a constant 1.5% discount rate, which is a common method in the Netherlands (other countries usually use uniform discounting). For all computations, the methods for cost-research of Hakkaart-vanRoijen et al.[68] will be used.

Missing values will not be completed by any means (e.g. average). In case of missing values, the fraction missing values will be stated. Multivariate analysis might therefore only be performed on a subset of patients (without missing values in all independent parameters), however due to the nature of the important independent parameters, we expect that this “subset” of patients will be over 95% of the study population.

All data will be presented quantitatively. All analysis will be performed according to the intention to treat. In case of study-arm cross-over (e.g. a PET-negative patient in the experimental study-arm who is operated upon due to high clinical suspicion of missed malignancy), this will be described.

10.2 Descriptive statistics

Variables are described using the parameters for central tendency and spread as displayed in Table 5, p65.

Scale:	Distribution:	Central Tendency:	Spread:
**Continuous**	Normal	Mean	95%-CI or full range
	Lognormal	Mean*	95%-CI*or full range*
	Not-normal	Median (= p_{50})	IQR or full range
**Categorical**	Binomial	Percentage	Exact Clopper-Pearson (β-distribution)

Table 5: Handling of descriptive statistics. *parameters are estimated on the logtransformed variable and back-transformed. 95%-CI: 95%-confidence interval using normal distribution (mean±1.9600 x standard deviation). IQR: interquartile range (p_{25} and p_{50}).

10.3 Univariate analysis

All comparisons will be made using the general requirements for relating research questions as stated in Table 6, p66.

Characteristics of (independent) study-arms will be compared using either t-tests ((log)normal) or Wilcoxon rank-sum tests for continuous variables (e.g. age, SUV, hospitalisation days, volumes of care used, questionnaire scores). For categorical data either Fisher exact tests or χ^2 tests will be used (e.g. sex, unbeneficial patient management).

With respect to the primary research question, fractions unbeneficial patient management therefore will be compared using Fisher exact tests or χ^2 tests.

Relations between variables will be univariately described by the parameters from Table 7, p66. These parameters are compared using the tests from Table 8, p67.

Scale:	Groups:	Dependency:	Group Size:	Distribution:	Test:
**Continuous (and categorical ordinal)**	1	n/a	n≥30	(Normal)	t-test
			n<30	Normal	
				Not normal but transformable	Transform than t-test
				Not normal, not transformable	Sign test
	2	Independent	n≥30	(Normal)	t-test
			n<30	Normal, equal variances	
				Normal, non-equal variances, equal n's	
				Normal, non-equal variances, non-equal n's, transformable	Transform than t-test
				Normal, non-equal variances, non-equal n's, not transformable	Wilcoxon rank-sum test (= Mann-Whitney U test)
				Not normal, transformable	Transform than t-test
				Not normal, not transformable	Wilcoxon rank-sum test (= Mann-Whitney U test)
		Dependent	n≥30	(Normal)	Paired t-test
			n<30	Normal	
				Not normal, transformable	
				Not normal, not transformable	Wilcoxon signed ranks
	≥3	Independent	n/a	Normal, 1 factor	One-Way ANOVA
				Normal, ≥2 factors	Two-Way or other ANOVA
				Not normal	Kruskal-Wallis for 1 Factor (+Dunn's posthoc test)
		Dependent	n/a	Normal	Repeaed Measures ANOVA
				Not normal	Friedman ANOVA
**Categorical (Nominal)**	1		np≥5 and n(1-p)≥5		z-approximation
	2	Independent	Expected cell frequencies <5		Fisher's Exact Test
			Expected cell frequencies ≥5		X^2 or z-approximation
		Dependent			McNemar or κ
	≥3	Independent	Expected cell frequencies <5		Collapse categories for X^2
			Expected cell frequencies ≥5		X^2
		Dependent			Cochran's Q

Table 6: General Requirements for selection of Statistical tests used for univariate analysis (comparison)

Scale:	Predicting each other	Parameter:
**Nominal**		Relative Risk
**Ordinal**		Spearman's ρ squared
**Numerical**	yes	Regression
	no	Pearson's Correlation R squared (log)normal<br>Spearman's ρ squared (not-normal)

Table 7: Parameters describing association between variables.

Statistic:	Number		
**Correlation Coefficient (R)**	1	R=0?	t-test
		R=value	Fisher's z Transformation
	2	Independent	
		Dependent	
**Regression Coefficients**	1		t-test
	2		

Table 8: Comparison between parameters describe association

Se, Sp, PPV and NPV will be computed using the traditional formulae, using the definition for true/false positive as described in Table 9, p67 , §8.3, p48:

$$Sensitivity: Se = \frac{TP}{TP+FN}, Specificity: Sp = \frac{TN}{TN+FP}$$

$$Accuracy: Acc = \frac{TP+TN}{TP+TN+FP+FN}, Prevalence: Prev = \frac{TP+FN}{TP+TN+FP+FN}$$

$$Positive\ Predictive\ Value: PPV = \frac{TP}{TP+FP}, Negative\ Predictive\ Value: NPV = \frac{TN}{TN+FN}$$

$$Positive\ Likelihood\ Ratio: LR+= \frac{Se}{1-Sp}, Negative\ Likelihood\ Ratio: LR-= \frac{1-Se}{Sp}$$

$$Diagnostic\ Odds\ Ratio: dOR = \frac{LR+}{LR-}, SE\big(LOG(dOR)\big) = \sqrt{\frac{1}{TP}+\frac{1}{TN}+\frac{1}{FP}+\frac{1}{FN}}$$ [69]

	Disease +ve	Disease –ve
**Test +ve**	TP	FP
**Test –ve**	FN	TN

Table 9: Definition of true positive (TP), true negative (TN), false-positive (FP) and false-negative (FN).

Threshold effects of FDG-uptake in de nodules will be assessed using receiver-operating characteristic (ROC) curves and quantified using the area under the curve (AUC), as Se and Sp are inversely related. The standard error (and thus the 95%-CI) of the AUC can be computed either under a bi-negative exponential assumption (used only when the number of positive actual states is equal to the number of negative actual states) or using a non-parametric assumption (used in all other cases).

10.4 Multivariate analysis

Where appropriate, multivariate analysis will be performed to show the independent contributions of various factors to a single event or outcome. By this means, factors are explored contributing to FDG-PET/CT false negativity, FDG-PET/CT uptake (SUV), (overall and disease-free) free survival, etc. Multivariate modelling will be performed based on the characteristics of the individual data, candidate variables must show univariate significant association. Iterative forward (1 to many) and backward (many to 1) models will be evaluated based on thresholds for in- and exclusion. See Table 10, p68.

Scale of Independent:	Scale of Dependent:	Observations:	Confounding Independent:	Model:
**Nominal**	Nominal	Censored		Kaplan-Meier
		Not censored	Yes	Mantel-Haenszel
			No	Log-Linear
	Numerical	Censored		Cox Proportional Hazard
		Not censored		ANOVA
**Nominal & Numerical**	Nominal	2-values dependent (binary)		Logistic Regression
		≥3 values dependent		Classification methods
	Numerical	Censored		Cox Proportional Hazard
		Not censored	Yes	ANCOVA
			No	Multiple Regression

Table 10: Models for assessment of independency in multivariate modelling.

10.5 Interim analysis

This has already been described in §8.7, p58.

10.6 Cost-Effectiveness Analysis (CEA)

General considerations:

This part of the study evaluates the potential efficiency of incorporating FDG-PET/CT in the diagnostic work-up versus usual practice in adult patients with FNAC-indeterminate thyroid nodules from a societal perspective. The economic evaluation is based on the general principles of a cost-effectiveness analysis and is performed alongside the randomised controlled clinical trial. Primary outcome measures for the economic evaluation, considering the 12 months follow-up period, are costs (direct and indirect) and quality adjusted life years (QALY). The incremental cost-effectiveness ratio (ICER) “cost per QALY gained” will be computed and uncertainty will be determined using the bootstrap method or Fieller method. A cost-effectiveness acceptability curve will be derived that is able to evaluate efficiency by using different thresholds (Willingness to Pay) for a QALY. The impact of uncertainty surrounding deterministic parameters (e.g. prices) on the ICER will be explored using one-way sensitivity analysis on the range of extremes. Secondary ICERs such as cost per unbeneficial patient management decrease will be presented and analysed as above. Furthermore, incremental Net Monetary Benefit (iNMB) will be computed based on a Willingness-to-Pay threshold of €80,000/QALY, as stated before.

Cost-analysis:

The cost-analysis exists of two main parts. First, on patient level, volumes of care will be measured prospectively using questionnaires filled-in by patients, completed by case record forms and patient files (supported by patient-based diaries). Per study-arm (experimental and control) full cost prices will be determined using activity-based costing (obviously) excluding the costs of the FDG-PET/CT in the control study arm. Cost items included are e.g.: surgical costs, costs of ^{131}I-RIA, admission costs, costs for complications (secondary surgery, ICU etc.), out-patient clinic visits, use of relevant medications (calcium, vitamin D, 69iothyronine69, 69iothyronine etc.), blood tests ((para)thyroid function tests) and additional investigations (US, CT, biopsy, histopathology, etc.). Productivity losses for patients (sick leave) will be estimated using the iPCQ. The friction cost-method will be applied following the Dutch guidelines [68]. Also travel time to an out-patient clinic and related expenses will be considered using the iMCQ. The second part of the cost-analysis consists of determining the cost prices for each volume of consumption in order to use these for multiplying the volumes registered for each participating patient. The Dutch guidelines for cost-analysis will be used [68]. For units of care/resources where no guideline of standard prices is available, real cost prices will be determined.

Patient outcome analysis:

The effect analysis adheres to the design of a randomised controlled trial and measures at baseline (T0) and after surgery+^{131}I-RIA/2 months, 6 months and 12 months (T1-3) follow-up. To measure the quality of the health status of the patients, a validated HRQoL instrument will be used: the EQ-5D-5L. This HRQoL instrument will be completed by the patients and is available in a validated Dutch translation [70]. The EQ-5D-5L is a generic HRQoL instrument comprising five domains: mobility self-care, usual activities, pain/discomfort and anxiety/depression. The EQ-5D-5L index is obtained by applying predetermined weights to five domains. This index gives a societal-based global quantification of the patients' health status on a scale ranging from 0 (death) to 1 (perfect health). Patients will also be asked to rate their overall HRQoL on a visual analogue scale (EQ-5D-VAS) consisting of a vertical line ranging from 0 (worst imaginable health status) to 100 (best imaginable).

11. ETHICAL CONSIDERATIONS

11.1 Regulation statement

This study will be conducted according to the principles of the Declaration of Helsinki (version of the 59th WMA General Assembly, Seoul, October 2008) and in accordance with the Medical Research Involving Human Subjects Act (WMO), the European Union (EU) General Data Protection Regulation (GDPR), the National Thyroid Cancer guideline [5] and the local (hospital) guidelines.

11.2 Recruitment and consent

Clinicians (especially endocrinologists) of participating hospitals will check in- and exclusion criteria when they have a patient with indeterminate FNAC for a thyroid nodule. They will inform the patient on the study. Patient will be permitted at least 48 hours of time to consider their decision and will be given the opportunity to discuss remaining questions about study participation with their treating physician. After this, written informed consent will be obtained. At the request of the patient, we will permit the informed consent procedure to take place through mail or email. Once written informed consent has been obtained, cytological material will be sent for central revision and thereafter patients are registered centrally.

The treating physician will hear whether the patient can be entered in the study, i.e. all inclusion criteria are met and all exclusion criteria are excluded (preferably within 2 working days from registration).

Once the patient is randomised (and has a study ID), an FDG-PET/CT of the head and neck can be planned, preferably by the treating clinical physician. The coordinating investigator will inform the department of nuclear medicine of the participating hospital on the acquisition protocol and will assure no report will follow. The images will be sent to the coordinating investigator who will review them and will inform the treating physician whether or not surgery is deemed unnecessary (§8.3.1, p48) and a confirmatory US should be planned (§8.3.2, p52). This feedback will take place within one week after the FDG-PET/CT has taken place. For the patient information letter and informed consent form see Appendix IX: Patient information Letter, declaration of insurance, informed consent and travelling expenses declaration form (in Dutch), §15.7, p98.

11.3 Objection by minors or incapacitated subjects

Minors are not included (§4.2, p36) and incapacitated adults are excluded from this study (§4.3, p37). Thyroid nodules and DTC are rare in children.

11.4 Benefits and risks assessment, group relatedness

The benefits for the individual patient is that he or she as a 2/3 probability to be included in the experimental arm in which only ~60-65% will be operated upon (instead of ~100% in the control study-arm, which is considered standard care). By lowering the amount of unnecessary invasive procedures also the number of complications and side effects are lowered.

As described elsewhere in detail (§13, p78), the risk for the individual patients are mainly based on delayed treatment for the indolent growing DTC in case of a false-negative PET-scan in the experimental study arm and the risks attached to the use of ionising radiation and of incidental PET findings.

The risks of consequences of delayed treatment for DTC are minimised by using a confirmatory US in the individual patient (§8.3.2, p52) and by performing interim- (§8.7, p58) and posthoc (§8.4.1, 56) analyses on the control study arm (in which both FDG-PET/CT and final histology is obtained).

The risk of ionising radiation (§15.6, p97) is minimised by several means: using (ultra) low-dose CT, minimising the scan range, thus minimising the amount if tissue exposed to (ultra) low-dose CT radiation and exclusion of minors that would both have increased effective dose per amount of FDG and suffer more detriment per unit of effective dose. By decreasing the scan range, the scan time is also limited, encouraging nuclear medicine departments to scan at a decreased speed (e.g. 4 min per bedposition or longer) by which means they can lower the administered FDG-dose.

The risks of additional (invasive) diagnostic procedures for incidental PET-findings is minimised by only scanning head, neck and thoracic inlet.

The burden to the patients consist of a single FDG-PET/CT (1.5h, in all patients, §8.3.1, p48), a single confirmatory US (0.5 h, in ~35-40% of the experimental arm or approximately ~25% of all patients included, §8.3.2, p52) and 4 times 6 questionnaires (120-160 min, in all patients, §8.3.3, p52). On average these 3 types of investigation therefore take in total 4-4.5 h per patient.

Travel expenses of included patients to and from the hospital will be compensated for the FDG-PET/CT and the confirmatory US (€ 0.19/km or comparable and possible additional costs for parking at the hospital, according to the locally set reimbursement rates and in consultation with the project leaders).

11.5 Compensation for injury

The sponsor/investigator has a liability insurance which is in accordance with article 7, subsection 9 of the WMO.

The sponsor (also) has an insurance which is in accordance with the legal requirements in the Netherlands (Article 7 WMO and the Measure regarding Compulsory Insurance for Clinical Research in Humans of 23th June 2003). This insurance provides cover for damage to research subjects through injury or death caused by the study.
€ 450.000,-- (i.e. four hundred and fifty thousand Euro) for death or injury for each subject who participates in the Research;
€ 3.500.000,-- (i.e. three million five hundred thousand Euro) for death or injury for all subjects who participate in the Research;
€ 5.000.000,-- (i.e. five million Euro) for the total damage incurred by the organisation for all damage disclosed by scientific research for the Sponsor as ‘verrichter’ in the meaning of said Act in each year of insurance coverage.

The insurance applies to the damage that becomes apparent during the study or within 4 years after the end of the study.

11.6 Incentives

Travel expenses of the included patients to and from the hospital will be compensated for the FDG-PET/CT and the confirmatory US (€ 0.19/km or comparable and possible additional costs for parking at the hospital, according to the locally set reimbursement rates and in consultation with the project leaders).

12. ADMINISTRATIVE ASPECTS, MONITORING AND PUBLICATION

12.1 Handling and storage of data and documents

All data will be handled confidentially and pseudonymously and is stored in a secured online database (CRF-file). Access to this database is possible via the internet but is restricted and password-protected with different levels of accessibility for different users. Any changes to this database are logged to permit traceability.

This database containing all information will be coded (pseudonymised) using the individual patient study-ID obtained directly after randomisation (the key is solely known to the coordinating investigator). This key is based on inclusion order (e.g. a number between 1 and 132) and therefore is not based on initials, birth-date, hospital or study allocation.

As patient information such as sex, birth-date, FDG-PET/CT date and FDG-PET/CT hospital are stored in a database, they could in combination possibly be traced back to the individual patient. All raw data containing patient identification (the informed consent forms, the key to the code) will be stored in a locked archive (or when digitalised: encrypted with password protection).

The coordinating investigator needs to be aware of the name of the patient: he/she must be able to contact the treating physician of the patient with respect to the consequences of the FDG-PET/CT or be able to contact the patient for submission of questionnaires/recall of sick leave days (supported by diaries). Since the coordinating investigator is an M.D. and could be regarded as member of the treating team of the individual patient, this is considered justifiable.

All raw data (baseline characteristics, histo- and cytopathologic results, ultrasound and surgical reports, questionnaires, etcetera) will be submitted to electronic Case Report Forms (eCRFs) and stored coded by the individual patient study ID. We use the environment provided by CastorEDC (www.castoredc.com) for this purpose, which is supported by the Radboudumc and free of charge as of July 2015.

All pseudonymized FDG-PET/CT files will be submitted to, archived in and retrieved for analysis from a password-protected BMIA database, which is provided by the CTMM TraIT project (http://www.ctmm-trait.nl/). This environment allows uploading of the raw image files through a secure CTP-connection; right before uploading the images' DICOM headers that contain the patients' personal data are either erased or replaced by the patient study ID automatically. It does not allow uploading of files without replacing these DICOM headers. The CTP-servers are being installed and configured in each participating centre with help of local network administrators, and tested with a 'dummy scan' prior to use for study purposes.

According to the WGBO (Law on Medical Treatment Agreement), patient data should be archived for at least 15 years. As the Code of Conduct Health Research states that data should be saved as long as "they can reasonably be expected to be relevant for research". In practice

this means up till 5 years after publication of the last data. This is 4 years (inclusion) + 5 years (long-term follow-up) + 5 years (storage term) thus also approximately 15 years.

12.2 Monitoring and Quality Assurance

Data monitoring will be performed by an independent monitor periodically. A data monitoring plan is composed (§15.7: Appendix VII: Data Monitoring Plan, p98) based on the guidelines for 'Kwaliteitsborging mensgebonden onderzoek 2.0' as proposed by the Nederlandse Federatie van Universitair Medische Centra (NFU) [71].
Apart from annual progress reports (§12.4, p76) and monitoring by the Safety Committee (§9.5, p62) no structural monitoring by an independent committee will be performed.

12.3 Amendments

Amendments are changes made to the research after a favourable opinion by the accredited METC has been given. All amendments will be notified to the METC that gave a favourable opinion.
The documentation that will be included in the submission should cover the following information:

- Covering letter, including the reasons for the amendment in one or two sentences, a brief description of the changes that are included in the amendment and the name of the documents that are modified;
- An extract of the modified documents, where applicable, showing both the previous and new wording, where applicable.
- The new version of the modified documents, where applicable, identified with updated number of version and date.

All substantial amendments will be notified to the METC and to the competent authority.

Non-substantial amendments will not be notified to the accredited METC and the competent authority, but will be recorded and filed by the sponsor. (Examples of non-substantial amendments are: typing errors and administrative changes like changes in names, telephone numbers and other contact details of involved persons mentioned in the submitted study documentation).

12.4 Report of start of inclusion

The grant supplier (Dutch Cancer Society), requires a written statement when the scientific personnel is hired. This person must be contracted within 12 months after the grant has awarded (i.e. February 21st, 2015, at latest). The METC will be notified once the first patient is included.

12.5 Annual progress report

The sponsor/investigator will submit a summary of the progress of the trial to the accredited METC once a year. Information will be provided on the date of inclusion of the first subject, numbers of subjects included and numbers of subjects that have completed the trial, serious adverse events/ serious adverse reactions, other problems, and amendments.

12.6 Interim Evaluation Report

An interim evaluation report is mandated by the grant supplier (Dutch Cancer Society), 18 months after inclusion of the first patient.

12.7 End of study report

The investigator will notify the accredited METC of the end of the study within a period of 8 weeks. The end of the study is defined as the date 1 year after the FDG-PET/CT of the last included patient.

In case the study is ended prematurely, the investigator will notify the accredited METC within 15 days, including the reasons for the premature termination.

Within one year after the end of the study, the investigator/sponsor will submit a final study report with the results of the study, including any publications/abstracts of the study, to the accredited METC.

An end-of-study report is also sent to the grant supplier (Dutch Cancer Society), 12 months after the end of the study.

12.8 Public disclosure and publication policy

Public disclosure and publication policy is derived from the European Organisation for Research and Treatment of Cancer (EORTC) Disclosure of Results and Publication Policy POL009 Version 4.02 [72] and is in accordance with the International Committee of Medical Journal Editors (Vancouver Group) - Uniform Requirements for Manuscripts Submitted to Biomedical Journals (http://www.icmje.org/) and the Revised 2010 CONSORT statement

(http://www.consort-statement.org/consort-statement). [73, 74] This trial will be prospectively registered in a public trial registry.[75]

The main trial results will be disclosed to the (scientific) public by means of scientific publications. Manuscripts for these publications will be written on the basis of the final analysis performed at the Radboudumc. Manuscripts will be offered to (major) peer-reviewed scientific journals, regardless of positive or negative trial results.

The final publications of associated translational research studies will be written by the Coordinator of the corresponding translational research study (e.g. Pathologist or partner from HTA group).

Authors of the manuscript(s) will include the Project Committee (at least: two from the dept. of nuclear medicine, one from the dept. of endocrinology, one from the dept. of endocrinological oncological surgery, one of the dept. of pathology and one of the dept. of HTA, all of the Radboudumc). The clinical research physician in charge of the trial at the Radboudumc will co-author all manuscript directly following from this trial.

From the multicentre site partners, the number of authors per site is dependent on the number of included patients. One author is permitted from a site in case it included >15% individual patients (or ≥20 included patients that were not withdrawn), two authors are permitted in case a site included >20% individual patients (or ≥27 included patients that were not withdrawn).

All other investigators who contributed patients to the study (i.e. clinicians) or contributed scientifically to the study (i.e. pathologists, collaborators from the same institutions, etc.) other than the Project Committee are acknowledged in the publication. The acknowledgement list should include the name of all participating institutions and the name of the clinicians and other scientists involved with the study at that institution. Whenever a study participant has moved from one institution to another in the course of the study, that participant is listed with the institution to which he/she was affiliated at the time of starting his/her participation to the study, with the mention “(now at (new affiliation))”. Also, sources of funding and supporting bodies will be acknowledged in the publications.

Prior to submission, all publications (papers, abstracts, presentations) including data pertaining to patients from the present trial will be submitted for review to the Radboudumc, to all co-authors.

The above rules are applicable to publications involving any individual patient registered/randomised in the trial.

13. STRUCTURED RISK ANALYSIS

13.1 Potential issues of concern

13.1.1 FDG-PET/CT

See also: §15.6: Appendix VI: Radiation Ethics Form, p97

a. Level of knowledge about mechanism of action

FDG is widely used for various oncological and non-oncological cases. Approximately 1.5-1.7 million FDG-PET-scans are performed in the US and 0.9 million in Europe, yearly. Over 3,000 FDG-PET-scans are currently performed per million population in the Benelux, so the experience is vast. FDG is handled in the body similarly as glucose. After injection in the bloodstream, FDG is rapidly taken up in the cells by the sodium dependent membrane-bound glucose transporters (GLUT). The rate of uptake is highly dependent on energy-needs of the particular tissue. There FDG will enter glycolysis like D-glucose, but after initial phosphorylation to FDG-6-phosphate, further catabolism is impossible due to the fact that the 2-oxygen is missing. Therefore, FDG-6-phosphate accumulates in the cells and especially those of high metabolic needs (brain, myocardium, liver, kidney, immune-cells, most malignant cells). As this tracer is attached to the positron emitter ^{18}F, positron emission arising from the decay of this isotope detected by the PET scanner represents the distribution of FDG in the tissue. After decay of ^{18}F to the stable ^{18}O, this "heavy oxygen" will pick up an H^+ from the environment and will become glucose-6-phosphate and is thereafter metabolised normally in the same way as ordinary glucose. No adverse effects are known for FDG, both due to the fact that it is similar to D-glucose and that it is administered in sub-nanomoll doses. The risk attached to a FDG-PET/CT-scan are therefore limited to the radiation of FDG (0.019mSv/ MBq or ~2mSv for a 70- kg adult according to the Radboudumc dose) and of the low-dose CT (~0.5-0.7mSv). The radiation attributed risk of (cancer related) death is estimated to be around 1.0-7.5 per 100,000 patients (age dependent, for adults, ICRP60) per mSv exposure (i.e. ~3-20 per 100,000 patients). According to the ICRP this corresponds to a risk class IIb: "modest" (Table 11, p79). The hospital radiation safety board provided a written report on the safety on this investigation which is included in the dossier (§15.6: Appendix VI: Radiation Ethics Form, p97).

Adult Effective Dose (mSv)	Risk-class:	Risk-significance:	Social Benefit:
**<0.1**	I	Trivial	Minimal
**0.1-1**	IIa	Minimal	Modest
**1-10**	IIb	Modest	Moderate
**>10***	III	Moderate	Substantial

Table 11: Risk classification according to the ICRP in relation with social benefit. *with the exeption of experimental-therapeutic applications, the dose needs to be below the threshold for deterministic effects.

b. Previous exposure of human beings with the test product(s) and/or products with a similar biological mechanism

As stated before, the experience in humans is vast.

c. Can the primary or secondary mechanism be induced in animals and/or in ex-vivo human cell material?

N/a.

d. Selectivity of the mechanism to target tissue in animals and/or human beings

FDG mainly accumulates in tissues with high-energy demands. Patients are asked to fast and to refrain from exercise after injection of FDG to minimize accumulation in muscle. Patients are pre-hydrated for forced diuresis to ensure rapid clearance from the blood, to lower whole-body radiation dose.

e. Analysis of potential effect

As stated before, no pharmacologic effects are expected (based on structural homology and subnanomoll dose) or are known from experience with FDG. Radiation induced risks (stochastical risk) are elaborated elsewhere (§15.6: Appendix VI: Radiation Ethics Form, p97).

f. Pharmacokinetic considerations

^{18}F effective half-life is mainly determined by physical half-life of the tracer (109.771(20) minutes) as the biological half-life of the tracer is much longer. Rapid clearance from the blood pool is ensured by prehydration. Only in case of severe (predialysis) renal failure FDG-clearance from the blood compartment is decreased.

g. Study population

Thyroid nodules are most often seen in (older) adult patients. Distribution of comorbidities is similar to the normal aging population. Due to the excellent prognosis of DTC, any (semi-)urgent disease occurring at the same time, will usually be treated first and analysis of a thyroid nodule will be postponed.

h. Interaction with other products

No interactions of FDG and other products are known. Severe hyperglycaemia and the use of insulin interferes with FDG-pharmacokinetics and therefore these are exclusion criteria for this study (§4.3, p37).

i. Predictability of effect

N/a

j. Can effects be managed?

There are no known effects to be managed.

13.1.2 Possible delay of treatment for DTC

a. Level of knowledge about mechanism of action

In contrast to the other head and neck tumours, cervical lymph node metastases are only of limited (or no) prognostic value in multivariate analysis of PTC patients. In FTC the prognostic value is not very clear, but seems to be of at least some prognostic significance. The possibility for a locoregional recurrence increases when cervical lymph nodes are present [76], but this rarely is the cause of death in patients with DTC.

There seems to be some relation between DTC tumour size (T-stage) on 10-year disease-free and overall survival ($T_{1\text{-}4}$: 85%, 73%, 60%, 56% (for 10y DFS) and 100%, 93%, 87%, 72% (for 10y OS), respectively [77]). However after correction for age, vascular invasion, extracapsular invasion and MACIS score, only TNM stage remained.

The consequences of delayed treatment for DTC are considered to be minimal, due to the relative indolent growth of this disease and the limited influence on survival of transition from localised (stage I) to regional (stage II) disease (both 5-year overall survival 100%), all with good (curative) treatment options Appendix V: American Joint Committee on Cancer (AJCC) TNM classification of differentiated thyroid cancer, 7th edition, (§15.5 p94). [1]

b. Previous exposure of human beings with the test product(s) and/or products with a similar biological mechanism

Since the clinical implementation of fine needle aspiration cytology (FNAC) to diagnose thyroid carcinoma, few patients remain misdiagnosed as the false-negative rate of FNAC for malignancy in thyroid nodules is less than 5% (Appendix IV: Performance of FDG-PET/CT in indeterminate thyroid nodules, §15.4, p92). An observational retrospective study comparing 12 cases with DTC and benign/non-diagnostic FNAC with 39 controls with DTC and

suspicious/malignant FNAC. Cases had a significant longer time from FNAC to surgery than control group (1.67±1.43 years versus 0.32±0.46 years) and FTC (obviously) occurred significantly more often in cases than controls (33% versus 2.6%). With a mean follow-up time of 3.6 years (ranging: 2 months-7.2 years) there was 100% OS in both groups and a non-significant disease-free survival (5.6±0.48 versus 6.6±0.33 years). It is therefore concluded that in patients with DTC, the result of the FNAC performed before surgery was not an independent predictor of recurrences or mortality in the first 7 years of follow-up. Thus, false negative or nondiagnostic FNAC in a patient with DTC does not seem to be a primary prognostic factor, but it may reveal other adverse prognostic factors such as longer time to therapy and higher prevalence of follicular carcinoma that may influence long-term outcomes [78].

It is impossible to determine whether FNAC is false-negative without treating patients, as the Gold standard histology by diagnostic (hemi)thyroidectomy is also the cornerstone of treatment for DTC. So even though this is a retrospective study, at risk of bias (e.g. was the DTC in the false-negative thyroids found at the site of FNAC, in other words: was the interpretation false-negative or was there sampling error?), this results support cross-sectional evidence that delay of treatment is not of proven detrimental effect for patients.

One older study [79] retrospectively studied 100 consecutive patients with histologically proven DTC who had undergone preoperative FNAC. Fourteen of these patients had cancers that were not detected by (1-3x) FNAC, three of whom developed widespread disease. A single false-negative FNA delayed surgical treatment by 28 months, sometimes despite clinical evidence suggesting malignancy. Subjects whose tumours were not detected by FNAC experienced delayed treatment, had higher rates of vascular and capsular invasion, and were more likely to have persistent disease at follow up (hazard ratio 2.28). However, they only included patients with either FNAC (suspicion of) malignancy or clinical findings suggestive of neoplasm (i.e. enlarging nodule in most cases). The nodule growth can be estimated from their data to be an average diameter doubling time of 38.5 months (21-78 months). With a mean follow-up interval to surgery of over 3 years, the average node doubled in diameter. This is, according to the ATA guidelines [6], highly suspicious for malignancy. So by selecting these "control" patients the authors only described those false-negative FNACs that remained highly suspicious for malignancy. Patients with a false-negative FNAC with nodes that do not grow were excluded from analysis. The clinical effect of delayed treatment therefore is overestimated. By using the safety measures described earlier, patients with fast-growing nodes will always be treated by surgery within 12 months (i.e. the time point for the confirmatory US).

c. Can the primary or secondary mechanism be induced in animals and/or in ex-vivo human cell material?
N/a.

d. Selectivity of the mechanism to target tissue in animals and/or human beings
N/a.

e. Analysis of potential effect
As stated above, we believe that the effect of delayed treatment for the unlikely event of a false-negative FDG-PET/CT is very limited. In case the false-negative rate (observed in the control study arm) will significantly exceed the 5% (calculated halfway through and at the end of the trial, §4.5, p40 and §8.7, p58), the study will be terminated preliminary and all patients with a negative FDG-PET/CT in the study arm will be advised to undergo surgery nonetheless.

f. Pharmacokinetic considerations
N/a.

g. Study population
See §13.1.1, p78.

h. Interaction with other products
N/a.

i. Predictability of effect
An effect on the individual is not predictable. About 3.6% of all negative FDG-PET/CTs are expected to false-negative (~1.3% of all patients: Appendix IV: Performance of FDG-PET/CT in indeterminate thyroid nodules, §15.4, p92.

j. Can effects be managed?
Yes, both due to the confirmatory US and the analyses on the control study arm we expect to be able to keep the number of patients with thyroid malignancies who will not get surgery within a year below 5% (i.e. similar to the risk of malignancy in any thyroid nodule which did not have FNAC).

13.2 Synthesis

The measures that have been taken to reduce the risk are:

- Exclusion of patients with a very high prior risk on thyroid malignancy,
- Exclude patients with potential false-negative FDG-PET/CT scans (e.g. hyperglycaemia, deregulated diabetes mellitus),
- Exclude patients with FDG-PET/CT scans which will be very difficult to interpret (e.g. multinodular goitre without dominant node, obvious infection to the neck),
- Optimised PET-acquisition with administered doses as low as reasonably achievable, limiting the investigated body area and increasing the speed of clearance of the radiopharmaceutical by forced diuresis,
- Exclusion of patient with highest amount of effective dose contributed to ionising radiation and highest amount of detriment (i.e. minors),
- Prevention of (irrelevant) incidental PET findings leading to (invasive and costly) diagnostic procedures by scanning only the head, neck and thoracic inlet,
- Centralised, double-reading of the FDG-PET/CT,
- Centralised, revision of cytological material (if definite benign or (suspicious of) malignancy, patients will be excluded from the study (i.e. not indeterminate FNAC) and treated accordingly,
- Confirmatory US in FDG-PET/CT-negative patients in the experimental study-arm,
- Interim en end-of-study analysis on the control study arm to verify that the FDG-PET/CT false negative will not exceed 5% by an independent board (safety committee §9.5: Data Safety Monitoring Board (DSMB) / Safety Committee, p62) consisting of a statistician, clinician and medical imager.
- Planned long-term follow-up to prevent an underestimation of the false-negative fraction of PET due to the indolent nature of the disease.
- In case explorative genetic testing shows mutations specific for malignancy (i.e. BRAF, TERT) but the patient did not undergo surgery (i.e. false-negative PET in the experimental arm), the treating physician will be informed of this (unexpected) finding.

Based on the recommendation of the European Science Foundation risk is classified according to Table 12, p84:

Amount of Injury: <br>Probability:	*Negligible:*	*Moderate:*	*Severe:*
Low	Negligible risk	Negligible risk	Moderate risk
Intermediate	Negligible risk	Moderate risk	High risk
High	Moderate risk	High risk	High risk

Table 12: Risk classification in relation to the probability and severity of potential injury.

As the risk for delayed treatment of differentiated thyroid carcinoma is expected to be 1-2% the probability of injury is considered low, and the extent of injury negligible to moderate and manageable, the risk is considered negligible.

As stated in §15.6: Appendix VI: Radiation Ethics Form, p97, the risk of the use of this amount of ionising radiation in this population is minor to intermediate and therefore considered negligible.

The probability of injury due to an incidental PET-finding is low and the injury following work-up of such a finding is considered negligible to moderate. Therefore, this risk is considered negligible.

Therefore, the risks of this study are negligible and acceptable for the subjects participating in the study, since:

1. Injury is unlikely to occur (<5%)
2. Potential injury is negligible to moderate in severity and is manageable
3. The potential positive effects (no unnecessary surgery and surgery-related sequelae) occur much more often and potentially outweigh the risks.

14. REFERENCES

1. Howlader, N., et al., *SEER Cancer Statistics Review, 1975-2008*, in *National Cancer Institute. Bethesda, MD*. 2011.
2. Cibas, E.S. and S.Z. Ali, *The Bethesda System for Reporting Thyroid Cytopathology.* Thyroid, 2009. **19**(11): p. 1159-65.
3. Cibas, E.S., S.Z. Ali, and N.C.I.T.F.S.o.t.S. Conference, *The Bethesda System For Reporting Thyroid Cytopathology.* Am J Clin Pathol, 2009. **132**(5): p. 658-65.
4. Vriens, D., et al., *The role of [18F]-2-fluoro-2-deoxy-d-glucose-positron emission tomography in thyroid nodules with indeterminate fine-needle aspiration biopsy: systematic review and meta-analysis of the literature.* Cancer, 2011. **117**(20): p. 4582-94.
5. Oncoline, *Evidence Based Nation-wide Guideline Thyroid Carcinoma version 1.1*. 2007, Integraal Kankercentrum Nederland IKNL.
6. Cooper, D.S., et al., *Revised American Thyroid Association management guidelines for patients with thyroid nodules and differentiated thyroid cancer (American Thyroid Association Guidelines Taskforce on Thyroid Nodules and Differentiated Thyroid Cancer).* Thyroid, 2009. **19**(11): p. 1167-214.
7. McHenry, C.R. and S.J. Slusarczyk, *Hypothyroidisim following hemithyroidectomy: incidence, risk factors, and management.* Surgery, 2000. **128**(6): p. 994-8.
8. Moon, H.G., et al., *Thyrotropin level and thyroid volume for prediction of hypothyroidism following hemithyroidectomy in an Asian patient cohort.* World J Surg, 2008. **32**(11): p. 2503-8.
9. Shaha, A.R., *TNM classification of thyroid carcinoma.* World J Surg, 2007. **31**(5): p. 879-87.
10. de Geus-Oei, L.F., et al., *18F-FDG PET reduces unnecessary hemithyroidectomies for thyroid nodules with inconclusive cytologic results.* J Nucl Med, 2006. **47**(5): p. 770-5.
11. Kresnik, E., et al., *Fluorine-18-fluorodeoxyglucose positron emission tomography in the preoperative assessment of thyroid nodules in an endemic goiter area.* Surgery, 2003. **133**(3): p. 294-9.
12. Kim, J.M., et al., *18F-fluorodeoxyglucose positron emission tomography does not predict malignancy in thyroid nodules cytologically diagnosed as follicular neoplasm.* J Clin Endocrinol Metab, 2007. **92**(5): p. 1630-4.
13. Sebastianes, F.M., et al., *Role of 18F-fluorodeoxyglucose positron emission tomography in preoperative assessment of cytologically indeterminate thyroid nodules.* J Clin Endocrinol Metab, 2007. **92**(11): p. 4485-8.
14. Hales, N.W., G.A. Krempl, and J.E. Medina, *Is there a role for fluorodeoxyglucose positron emission tomography/computed tomography in cytologically indeterminate thyroid nodules?* Am J Otolaryngol, 2008. **29**(2): p. 113-8.
15. Traugott, A.L., et al., *Exclusion of malignancy in thyroid nodules with indeterminate fine-needle aspiration cytology after negative 18F-fluorodeoxyglucose positron emission tomography: interim analysis.* World J Surg, 2010. **34**(6): p. 1247-53.
16. Munoz Perez, N., et al., *Could F-FDG-PET/CT avoid unnecessary thyroidectomies in patients with cytological diagnosis of follicular neoplasm?* Langenbecks Arch Surg, 2013.
17. Vriens, D., et al., *Cost-effectiveness of FDG-PET/CT for cytologically indeterminate thyroid nodules: a decision analytic approach.* J Clin Endocrinol Metab, 2014. **99**(9): p. 3263-74.
18. Li, H., et al., *Cost-effectiveness of a novel molecular test for cytologically indeterminate thyroid nodules.* J Clin Endocrinol Metab, 2011. **96**(11): p. E1719-26.
19. Husson, O., et al., *Health-related quality of life among thyroid cancer survivors: a systematic review.* Clin Endocrinol (Oxf), 2011. **75**(4): p. 544-54.
20. Deandreis, D., et al., *Is (18)F-fluorodeoxyglucose-PET/CT useful for the presurgical characterization of thyroid nodules with indeterminate fine needle aspiration cytology?* Thyroid, 2012. **22**(2): p. 165-72.

21. Schonberger, J., et al., *Glucose transporter 1 gene expression is related to thyroid neoplasms with an unfavorable prognosis: an immunohistochemical study.* Thyroid, 2002. **12**(9): p. 747-54.
22. Hooft, L., et al., *[18F]fluorodeoxyglucose uptake in recurrent thyroid cancer is related to hexokinase i expression in the primary tumor.* J Clin Endocrinol Metab, 2005. **90**(1): p. 328-34.
23. Kim, B.H., et al., *Relationship between biological marker expression and fluorine-18 fluorodeoxyglucose uptake in incidentally detected thyroid cancer.* Cancer Biother Radiopharm, 2010. **25**(3): p. 309-15.
24. Kaida, H., et al., *Relationship between clinicopathological factors and fluorine-18-fluorodeoxyglucose uptake in patients with papillary thyroid cancer.* Nucl Med Commun, 2011. **32**(8): p. 690-8.
25. Deichen, J.T., et al., *Influence of TSH on uptake of [18F]fluorodeoxyglucose in human thyroid cells in vitro.* Eur J Nucl Med Mol Imaging, 2004. **31**(4): p. 507-12.
26. Barollo, S., et al., *BRAF in primary and recurrent papillary thyroid cancers: the relationship with (131)I and 2-[(18)F]fluoro-2-deoxy-D-glucose uptake ability.* Eur J Endocrinol, 2010. **163**(4): p. 659-63.
27. Kleiman, D.A., et al., *Preoperative BRAF(V600E) mutation screening is unlikely to alter initial surgical treatment of patients with indeterminate thyroid nodules: A prospective case series of 960 patients.* Cancer, 2012.
28. de Koster, E.J., et al., *Diagnostic Utility of Molecular and Imaging Biomarkers in Cytological Indeterminate Thyroid Nodules.* Endocr Rev, 2018. **39**(2): p. 154-191.
29. Alexander, E.K., et al., *Preoperative diagnosis of benign thyroid nodules with indeterminate cytology.* N Engl J Med, 2012. **367**(8): p. 705-15.
30. Marti, J.L., et al., *Wide Inter-institutional Variation in Performance of a Molecular Classifier for Indeterminate Thyroid Nodules.* Ann Surg Oncol, 2015.
31. McIver, B., et al., *An independent study of a gene expression classifier (Afirma) in the evaluation of cytologically indeterminate thyroid nodules.* J Clin Endocrinol Metab, 2014. **99**(11): p. 4069-77.
32. Yang, S.E., et al., *Has Afirma gene expression classifier testing refined the indeterminate thyroid category in cytology?* Cancer Cytopathol, 2016. **124**(2): p. 100-9.
33. Eszlinger, M., et al., *Impact of molecular screening for point mutations and rearrangements in routine air-dried fine-needle aspiration samples of thyroid nodules.* Thyroid, 2014. **24**(2): p. 305-13.
34. Labourier, E., et al., *Molecular Testing for miRNA, mRNA, and DNA on Fine-Needle Aspiration Improves the Preoperative Diagnosis of Thyroid Nodules With Indeterminate Cytology.* J Clin Endocrinol Metab, 2015. **100**(7): p. 2743-50.
35. Ohori, N.P., et al., *"Colloid-rich" follicular neoplasm/suspicious for follicular neoplasm thyroid fine-needle aspiration specimens: cytologic, histologic, and molecular basis for considering an alternate view.* Cancer Cytopathol, 2013. **121**(12): p. 718-28.
36. Nikiforov, Y.E., et al., *Impact of mutational testing on the diagnosis and management of patients with cytologically indeterminate thyroid nodules: a prospective analysis of 1056 FNA samples.* J Clin Endocrinol Metab, 2011. **96**(11): p. 3390-7.
37. Yip, L., et al., *Cost impact of molecular testing for indeterminate thyroid nodule fine-needle aspiration biopsies.* J Clin Endocrinol Metab, 2012. **97**(6): p. 1905-12.
38. Steward, D.L., et al., *Performance of a Multigene Genomic Classifier in Thyroid Nodules With Indeterminate Cytology: A Prospective Blinded Multicenter Study.* JAMA Oncol, 2018.
39. Nikiforov, Y.E., et al., *Highly accurate diagnosis of cancer in thyroid nodules with follicular neoplasm/suspicious for a follicular neoplasm cytology by ThyroSeq v2 next-generation sequencing assay.* Cancer, 2014. **120**(23): p. 3627-34.
40. Nikiforov, Y.E., et al., *Impact of the Multi-Gene ThyroSeq Next-Generation Sequencing Assay on Cancer Diagnosis in Thyroid Nodules with Atypia of Undetermined*

Significance/Follicular Lesion of Undetermined Significance Cytology. Thyroid, 2015. **25**(11): p. 1217-23.
41. Kouniavsky, G. and M.A. Zeiger, *The quest for diagnostic molecular markers for thyroid nodules with indeterminate or suspicious cytology.* J Surg Oncol, 2012. **105**(5): p. 438-43.
42. Rago, T., et al., *Real-time elastosonography: useful tool for refining the presurgical diagnosis in thyroid nodules with indeterminate or nondiagnostic cytology.* J Clin Endocrinol Metab, 2010. **95**(12): p. 5274-80.
43. Piccardo, A., et al., *Thyroid nodules with indeterminate cytology: prospective comparison between 18F-FDG-PET/CT, multiparametric neck ultrasonography, 99mTc-MIBI scintigraphy and histology.* Eur J Endocrinol, 2016.
44. Mohey, N., T.A. Hassan, and S. Abdel-Baki, *Role of combined grey scale US and US tissue elastography in differentiating solid thyroid nodules.* Egyptian Journal of Radiology and Nuclear Medicine, 2013. **44**(3): p. 505-512.
45. Abdelrahman, S.F., et al., *Ultrasound elastography in the diagnostic evaluation of indeterminate thyroid nodule s.* Egyptian Journal of Radiology and Nuclear Medicine, 2015. **46**(3): p. 639-648.
46. Giovanella, L., et al., *Molecular imaging with Tc-MIBI and molecular testing for mutations in differentiating benign from malignant follicular neoplasm: a prospective comparison.* Eur J Nucl Med Mol Imaging, 2015.
47. Saggiorato, E., et al., *99mTc-MIBI Imaging in the presurgical characterization of thyroid follicular neoplasms: relationship to multidrug resistance protein expression.* J Nucl Med, 2009. **50**(11): p. 1785-93.
48. Netea-Maier, R.T., et al., *Trends in incidence and mortality of thyroid carcinoma in The Netherlands between 1989 and 2003: correlation with thyroid fine-needle aspiration cytology and thyroid surgery.* Int J Cancer, 2008. **123**(7): p. 1681-4.
49. Chung, A.Y., et al., *Metastases to the thyroid: a review of the literature from the last decade.* Thyroid, 2012. **22**(3): p. 258-68.
50. Boellaard, R., et al., *FDG PET and PET/CT: EANM procedure guidelines for tumour PET imaging: version 1.0.* Eur J Nucl Med Mol Imaging, 2010. **37**(1): p. 181-200.
51. Olson, M.T., et al., *A Tertiary Center's Experience With Second Review of 3885 Thyroid Cytopathology Specimens.* J Clin Endocrinol Metab, 2013.
52. Voutilainen, P.E., et al., *AMES, MACIS and TNM prognostic classifications in papillary thyroid carcinoma.* Anticancer Res, 2003. **23**(5b): p. 4283-8.
53. Trimboli, P., et al., *Clinical characteristics as predictors of malignancy in patients with indeterminate thyroid cytology: a meta-analysis.* Endocrine, 2014. **46**(1): p. 52-9.
54. Pocock, S.J. and R. Simon, *Sequential treatment assignment with balancing for prognostic factors in the controlled clinical trial.* Biometrics, 1975. **31**(1): p. 103-15.
55. Boellaard, R., et al., *FDG PET/CT: EANM procedure guidelines for tumour imaging: version 2.0.* Eur J Nucl Med Mol Imaging, 2015. **42**(2): p. 328-54.
56. Soelberg, K.K., et al., *Risk of malignancy in thyroid incidentalomas detected by 18F-fluorodeoxyglucose positron emission tomography: a systematic review.* Thyroid, 2012. **22**(9): p. 918-25.
57. Ware, J.E., Jr., *SF-36 health survey update.* Spine (Phila Pa 1976), 2000. **25**(24): p. 3130-9.
58. Brazier, J., J. Roberts, and M. Deverill, *The estimation of a preference-based measure of health from the SF-36.* J Health Econ, 2002. **21**(2): p. 271-92.
59. Herdman, M., et al., *Development and preliminary testing of the new five-level version of EQ-5D (EQ-5D-5L).* Qual Life Res, 2011. **20**(10): p. 1727-36.
60. van Roijen, L., et al., *Labor and health status in economic evaluation of health care. The Health and Labor Questionnaire.* Int J Technol Assess Health Care, 1996. **12**(3): p. 405-15.
61. Bouwmans, C., et al. *Handleiding iMTA Medical Cost Questionnaire (iMCQ)*. 2013; Available from: www.imta.nl.

62. Watt, T., et al., *Establishing construct validity for the thyroid-specific patient reported outcome measure (ThyPRO): an initial examination.* Qual Life Res, 2009. **18**(4): p. 483-96.
63. Tuinman, M.A., S.M. Gazendam-Donofrio, and J.E. Hoekstra-Weebers, *Screening and referral for psychosocial distress in oncologic practice: use of the Distress Thermometer.* Cancer, 2008. **113**(4): p. 870-8.
64. DeLellis, R.A., et al., *WHO classification of tumors, pathology and genetics - tumors of endocrine organs.* IARC Press, Lyon, 2004.
65. Haugen, B.R., et al., *American Thyroid Association Guidelines on the Management of Thyroid Nodules and Differentiated Thyroid Cancer Task Force Review and Recommendation on the Proposed Renaming of Encapsulated Follicular Variant Papillary Thyroid Carcinoma Without Invasion to Noninvasive Follicular Thyroid Neoplasm with Papillary-Like Nuclear Features.* Thyroid, 2017. **27**(4): p. 481-483.
66. *WHO Classification of Tumours of Endocrine Organs*. WHO Classification of Tumours, 4th edition, volume 10. 2017.
67. EMEA *EMEA Committee for Medicinal Products for Human Use (CHMP): Guideline on Data Monitoring Committees*. 2006.
68. Hakkaart-van Roijen, L., S.S. Tan, and C.A.M. Bouwmans, *Manual for Cost Research.Dutch manual, update appeared in 2004 and 2010. Published by the Dutch Government.* Available at: http://www.cvz.nl/binaries/content/documents/cvzinternet/nl/documenten/losse-publicaties/handleiding-kostenonderzoek-2010.pdf. Accessed March 2, 2012., ed. M.f.C. Research. 2010, Diemen: College voor Zorgverzekeringen.
69. Glas, A.S., et al., *The diagnostic odds ratio: a single indicator of test performance.* J Clin Epidemiol, 2003. **56**(11): p. 1129-35.
70. Lamers, L.M., et al., *[Measuring the quality of life in economic evaluations: the Dutch EQ-5D tariff].* Ned Tijdschr Geneeskd, 2005. **149**(28): p. 1574-8.
71. NFU, *Kwaliteitsborging mensgebonden onderzoek 2.0*. 2012, Nederlandse Federatie van Universitair Medische Centra.
72. Collette, L. and F. Meunier *Disclosure of Results and Publication Policy (POL009, version 4.02, 19/03/2012)*. 2012.
73. Davidoff, F., et al., *Sponsorship, authorship, and accountability.* N Engl J Med, 2001. **345**(11): p. 825-6; discussion 826-7.
74. *Uniform requirements for manuscripts submitted to biomedical journals. International Committee of Medical Journal Editors.* N Engl J Med, 1997. **336**(4): p. 309-15.
75. De Angelis, C., et al., *Clinical trial registration: a statement from the International Committee of Medical Journal Editors.* N Engl J Med, 2004. **351**(12): p. 1250-1.
76. Randolph, G., et al., *The Prognostic Significance of Nodal Metastases from Papillary Thyroid Carcinoma can be Stratified Based on the Size and Number of Metastatic Lymph Nodes, as Well as the Presence of Extranodal Extension ATA Surgical Affairs Committee's Taskforce on Thyroid Cancer Nodal Surgery.* Thyroid, 2012.
77. Gulcelik, M.A., et al., *Prognostic factors determining survival in patients with node positive differentiated thyroid cancer: a retrospective cross-sectional study.* Clin Otolaryngol, 2012. **37**(6): p. 460-7.
78. Garcia-Pascual, L., et al., *Prognostic factors and follow-up of patients with differentiated thyroid carcinoma with false negative or nondiagnostic FNAC before surgery. Comparison with a control group.* Endocrine, 2011. **40**(3): p. 423-31.
79. Yeh, M.W., et al., *False-negative fine-needle aspiration cytology results delay treatment and adversely affect outcome in patients with thyroid carcinoma.* Thyroid, 2004. **14**(3): p. 207-15.

15. APPENDICES

15.1 Appendix I: Participating Centres and Site Managers

On request of the METC, this section was removed to a separate document.

15.2 Appendix II: Signed Document of Cooperating Centres

On request of the METC, this section was removed to a separate document.

15.3 Appendix III: The Bethesda System for Reporting Thyroid Cytopathology

The Bethesda System for Reporting Thyroid Cytopathology: Recommended Diagnostic Categories [2, 3]:
I. Nondiagnostic or Unsatisfactory<br>*- Cyst fluid only*<br>*- Virtually acellular specimen*<br>*- Other (obscuring blood, clotting artifact, etc)*
II. Benign<br>*- Consistent with a benign follicular nodule (includes adenomatoid nodule, colloid nodule, etc)*<br>*- Consistent with lymphocytic (Hashimoto) thyroiditis in the proper clinical context*<br>*- Consistent with granulomatous (subacute) thyroiditis*<br>*- Other*
III. Atypia of Undetermined Significance or Follicular Lesion of Undetermined Significance
IV. Follicular Neoplasm or Suspicious for a Follicular Neoplasm<br>*- Specify if Hürthle cell (oncocytic) type*
V. Suspicious for Malignancy<br>*- Suspicious for papillary carcinoma*<br>*- Suspicious for medullary carcinoma*<br>*- Suspicious for metastatic carcinoma*<br>*- Suspicious for lymphoma*<br>*- Other*
VI. Malignant<br>*- Papillary thyroid carcinoma*<br>*- Poorly differentiated carcinoma*<br>*- Medullary thyroid carcinoma*<br>*- Undifferentiated (anaplastic) carcinoma*<br>*- Squamous cell carcinoma*<br>*- Carcinoma with mixed features (specify)*<br>*- Metastatic carcinoma*<br>*- Non-Hodgkin lymphoma*<br>*- Other*

Table 13: The Bethesda System for Reporting Thyroid Cytopathology. The orange shaded categories are included in this study.

15.4 Appendix IV: Performance of FDG-PET/CT in indeterminate thyroid nodules

Derived from Vriens *et al*. [4]

	FNAB Results, No.				FNAB Test Characteristics (95% CI), %					
Reference	TP	TN	FP	FN	Cancer Prevalence	Sensitivity	Specificity	NPV	PPV	Accuracy
Kresnik 2003[34a]	10	15	12	0	27 (13.8-44.1)	100 (69.2-100)	55.6 (35.3-74.5)	100 (78.2-100)	45.5 (24.4-67.8)	67.6 (50.2-82)
de Geus-Oei 2006[23]	6	25	13	0	13.6 (5.2-27.4)	100 (54.1-100)	65.8 (48.6-80.4)	100 (86.3-100)	31.6 (12.6-56.6)	70.5 (54.8-83.2)
Kim 2007[36a]	15	0	21	0	41.7 (25.5-59.2)	100 (78.2-100)	0 (0-16.1)	NaN	41.7 (25.5-59.2)	41.7 (25.5-59.2)
Sebastianes 2007[37]	11	12	19	0	26.2 (13.9-42)	100 (71.5-100)	38.7 (21.8-57.8)	100 (73.5-100)	36.7 (19.9-56.1)	54.8 (38.7-70.2)
Hales 2008[38]	5	3	6	1	40 (16.3-67.7)	83.3 (35.9-99.6)	33.3 (7.5-70.1)	75 (19.4-99.4)	45.5 (16.7-76.6)	53.3 (26.6-78.7)
Traugott 2010[41]	8	25	16	2	19.6 (9.8-33.1)	80 (44.4-97.5)	61 (44.5-75.8)	92.6 (75.7-99.1)	33.3 (15.6-55.3)	64.7 (50.1-77.6)
Pooled	55	80	87	3	25.8 (20.2-32)	94.8 (85.6-98.9)	47.9 (40.1-55.8)	96.4 (89.9-99.2)	38.7 (30.7-47.3)	60 (53.3-66.5)

FNAB indicates fine-needle aspiration biopsy; CI, confidence interval; TP, true-positive; TN, true-negative; FP, false-positive; FN, false-negative; NPV, negative predictive value; PPV, positive predictive value; NaN, not a number.
[a] Presented data vary from published data: Not all patients who were included in publications could be included in the current meta-analysis.

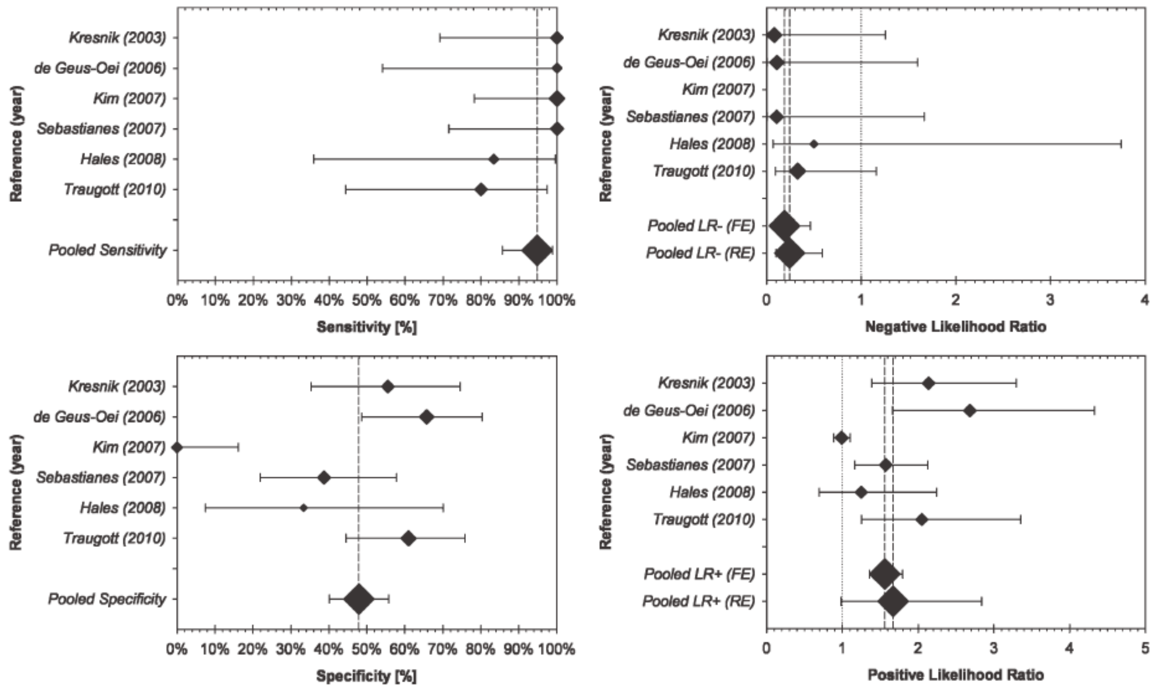

Figure 8: Results from meta-analysis on test characteristics of FDG-PET/CT in indeterminate thyroid noudles [4]. CI: confidence interval; FE: fixed-effects; FN: false negative; FNAB: fine-needle aspiration biopsy; FP: false positive; LR-: negative likelihood ratio; LR+: positive likelihood ratio; NPV: negative predictive value; PPV: positive predictive value; RE: random-effects; TN: true negative; TP: true positive.

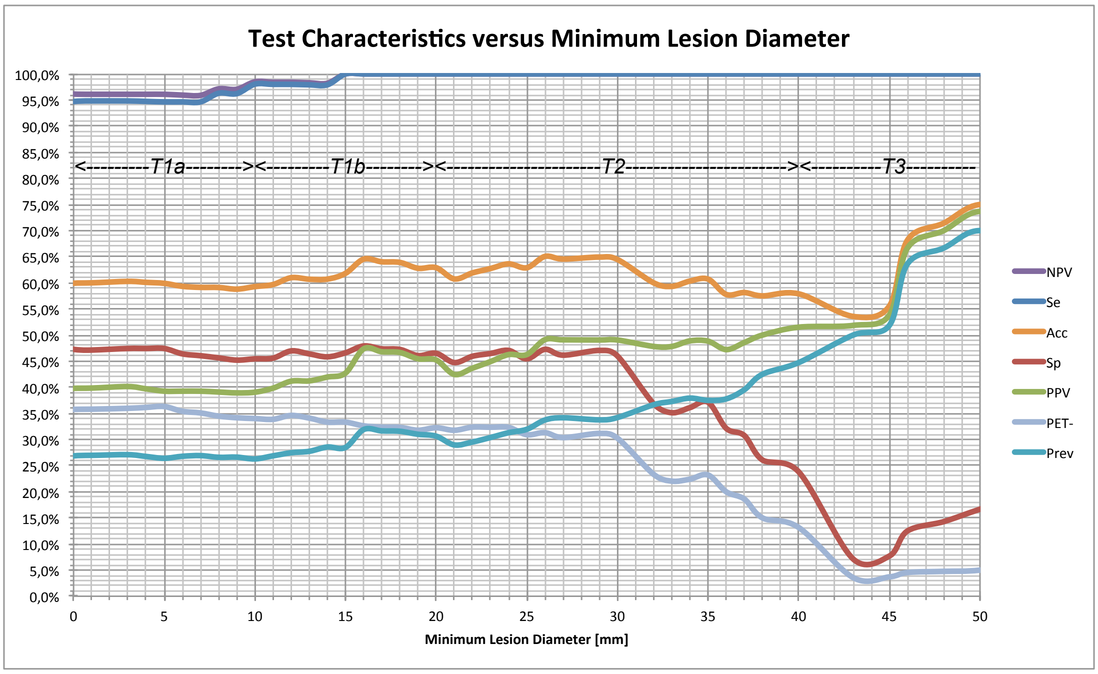

Figure 9: Test characteristics of FDG-PET for detection of malignancy in FNAC-indeterminate thyroid nodules based on definite size (histopathology). Cut-offs, based on the TNM-classification (7th revision) are displayed. Unpublished data from [4]. Acc: accuracy; NPV: negative predictive value; PET-: fraction of PET-negative nodules; PPV: positive predictive value; Se: sensitivity; Sp: Specificiy. For lesion-diameter ≥15mm, sensitivity is 1005 (92-100%), specificity is 47% (38-56%).

15.5 Appendix V: American Joint Committee on Cancer (AJCC) TNM classification of differentiated thyroid cancer, 7th edition

15.5.1 Introduction

Not well-differentiated thyroid cancer (medullary or anaplastic thyroid carcinoma) has a different TNM classification and staging. These are deliberately omitted.

15.5.2 Primary Tumour (T)

All categories may be subdivided: (s) solitary tumour and (m) multifocal tumour (the largest determines the classification).

**TX**	Primary tumour cannot be assessed.
**T0**	No evidence of primary tumour.
**T1**	Tumour ≤2 cm in greatest dimension limited to the thyroid.
**T1a**	Tumour ≤1 cm, limited to the thyroid.
**T1b**	Tumour >1 cm but ≤2 cm in greatest dimension, limited to the thyroid.
**T2**	Tumour >2 cm but ≤4 cm in greatest dimension, limited to the thyroid.
**T3**	Tumour >4 cm in greatest dimension limited to the thyroid or any tumour with minimal extrathyroid extension (e.g., extension to sternothyroid muscle or perithyroid soft tissues).
**T4a**	Moderately advanced disease.<br>Tumour of any size extending beyond the thyroid capsule to invade subcutaneous soft tissues, larynx, trachea, oesophagus, or recurrent laryngeal nerve.<br>Very advanced disease.
**T4b**	Tumour invades prevertebral fascia or encases carotid artery or mediastinal vessels.

Table 14: T(NM) stage of differentiated thyroid carcinoma, 7th edition.

15.5.3 Regional Lymphnodes (N)

Regional lymphnodes are the central compartment, lateral cervical, and upper mediastinal lymphnodes.

**NX**	Regional lymphnodes cannot be assessed.
**N0**	No regional lymphnode metastasis.
**N1**	Regional lymphnode metastasis.
**N1a**	Metastases to Level VI (pretracheal, paratracheal, and prelaryngeal/Delphian lymphnodes).
**N1b**	Metastases to unilateral, bilateral, or contralateral cervical (Levels I, II, III, IV, or V) or retropharyngeal or superior mediastinal lymphnodes (Level VII).

Table 15: (T)N(M) stage of differentiated thyroid carcinoma, 7th edition.

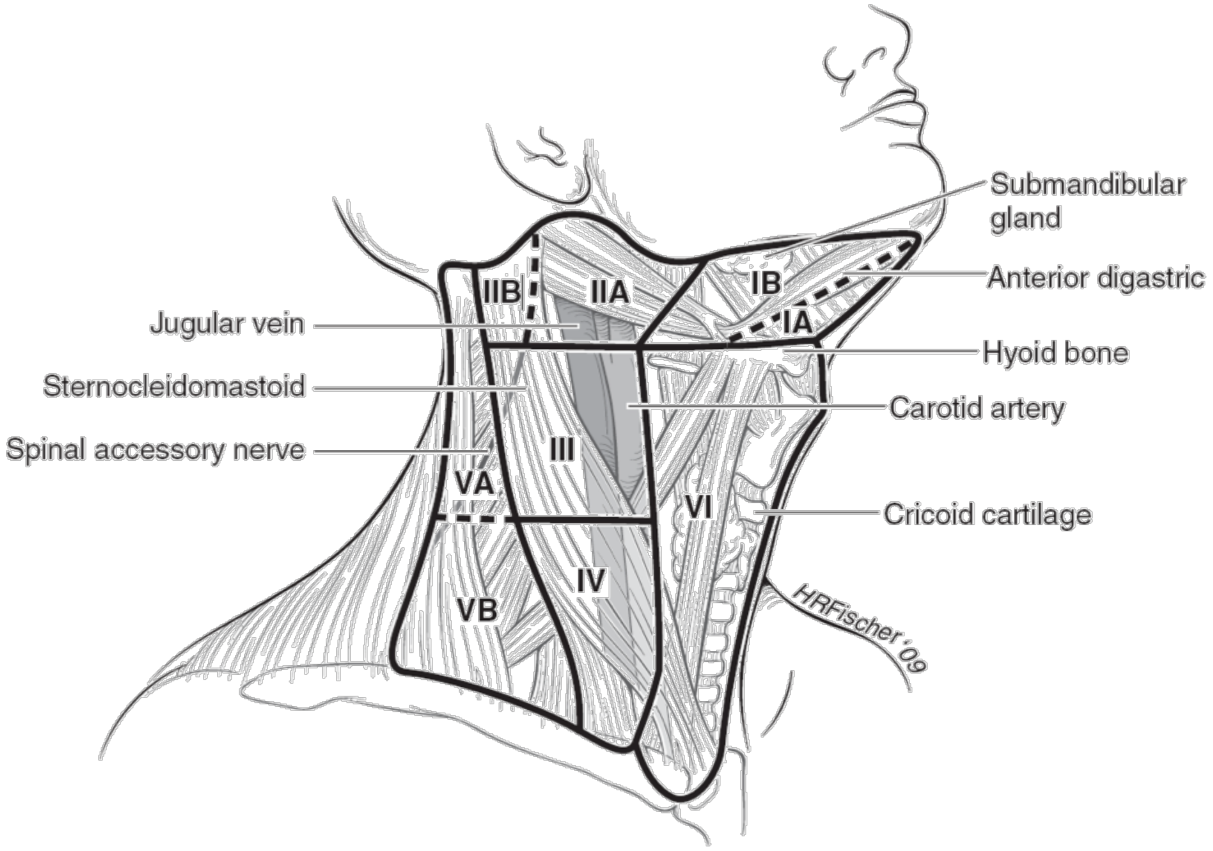

Figure 10: Lymphnode compartments separated into levels and sublevels.

Level VI contains the thyroid gland, and the adjacent nodes bordered superiorly by the hyoid bone, inferiorly by the innominate (brachiocephalic) artery, and laterally on each side by the carotid sheaths. The level II, III, and IV nodes are arrayed along the jugular veins on each side, bordered anteromedially by level VI and laterally by the posterior border of the sternocleidomastoid muscle. The level III nodes are bounded superiorly by the level of the hyoid bone, and inferiorly by the cricoid cartilage; levels II and IV are above and below level III, respectively. The level I node compartment includes the submental and submandibular nodes, above the hyoid bone, and anterior to the posterior edge of the submandibular gland. Finally, the level V nodes are in the posterior triangle, lateral to the lateral edge of the sternocleidomastoid muscle. Levels I, II, and V can be further subdivided as noted in the figure. The inferior extent of level VI is defined as the suprasternal notch. Many authors also include the pretracheal and paratracheal superior mediastinal lymphnodes above the level of the innominate artery (sometimes referred to as level VII) in central neckdissection [6].

15.5.4 Distant Metastasis

**M0**	No distant metastasis.
**M1**	Distant metastasis.

Table 16: (TN)M stage of differentiated thyroid carcinoma, 7th edition.

15.5.5 Anatomic Stage / Prognostic Groups

**Stage**	**Age < 45 years:**			**Age ≥ 45 years:**		
	**T**	**N**	**M**	**T**	**N**	**M**
**I**	Any T	Any N	M0	T1	N0	M0
**II**	Any T	Any N	M1	T2	N0	M0
**III**				T3	N0	M0
				T1-T3	N1a	M0
**IVa**				T4a	N0-N1a	M0
				T1-T3	N1b	M0
				T4a	N1b	M0
**IVb**				T4b	Any N	M0
**IVc**				Any T	Any N	M1

Table 17: Anatomic staging of differentiated thyroid carcinoma

15.6 Appendix VI: Radiation Ethics Form

On request of the METC, this section was removed to a separate document.

15.7 Appendix VII: Data Monitoring Plan

On request of the METC, this section was removed to a separate document.

15.8 Appendix VIII: Questionnaires

On request of the METC, this section was removed to a separate document.

15.9 Appendix IX: Patient information Letter, declaration of insurance, informed consent and travelling expenses declaration form (in Dutch)

On request of the METC, this section was removed to a separate document.
